# Chromosome-level genome assembly and population genomic analyses provide insights into adaptive evolution of the red turpentine beetle, *Dendroctonus valens*

**DOI:** 10.1186/s12915-022-01388-y

**Published:** 2022-08-24

**Authors:** Zhudong Liu, Longsheng Xing, Wanlong Huang, Bo Liu, Fanghao Wan, Kenneth F. Raffa, Richard W. Hofstetter, Wanqiang Qian, Jianghua Sun

**Affiliations:** 1 College of Life Science, Institute of Life Science and Green Development, Hebei University, Baoding, 071002 China; 2 State Key Laboratory of Integrated Management of Pest Insects and Rodents, Institute of Zoology, Chinese Academy of Sciences, Beijing, 1000101 China; 3 Shenzhen Branch, Guangdong Laboratory for Lingnan Modern Agriculture, Genome Analysis Laboratory of the Ministry of Agriculture and Rural Affairs, Agricultural Genomics Institute at Shenzhen, Chinese Academy of Agricultural Sciences, Shenzhen, 518120 China; 4 Novogene Bioinformatics Institute, Beijing, China; 5 Department of Entomology, University of Wisconsin, Madison, WI 53706 USA; 6 School of Forestry, Northern Arizona University, Flagstaff, AZ 86011 USA

**Keywords:** Biological invasion, Red turpentine beetle, Genomics, Population genetic structure, Selective sweep, Adaptive evolution

## Abstract

**Background:**

Biological invasions are responsible for substantial environmental and economic losses. The red turpentine beetle (RTB), *Dendroctonus valens* LeConte, is an important invasive bark beetle from North America that has caused substantial tree mortality in China. The lack of a high-quality reference genome seriously limits deciphering the extent to which genetic adaptions resulted in a secondary pest becoming so destructive in its invaded area.

**Results:**

Here, we present a 322.41 Mb chromosome-scale reference genome of RTB, of which 98% of assembled sequences are anchored onto fourteen linkage groups including the X chromosome with a N50 size of 24.36 Mb, which is significantly greater than other Coleoptera species. Repetitive sequences make up 45.22% of the genome, which is higher than four other Coleoptera species, i.e., Mountain pine beetle *Dendroctonus ponderosae*, red flour beetle *Tribolium castaneum*, blister beetle *Hycleus cichorii*, and Colorado potato beetle *Leptinotarsa decemlineata*. We identify rapidly expanded gene families and positively selected genes in RTB, which may be responsible for its rapid environmental adaptation. Population genetic structure of RTB was revealed by genome resequencing of geographic populations in native and invaded regions, suggesting substantial divergence of the North American population and illustrates the possible invasion and spread route in China. Selective sweep analysis highlighted the enhanced ability of Chinese populations in environmental adaptation.

**Conclusions:**

Overall, our high-quality reference genome represents an important resource for genomics study of invasive bark beetles, which will facilitate the functional study and decipher mechanism underlying invasion success of RTB by integrating the *Pinus tabuliformis* genome.

**Supplementary Information:**

The online version contains supplementary material available at 10.1186/s12915-022-01388-y.

## Background

With increasing international trade, biological invasion is a critical concern worldwide, threatening environmental quality and economic security, incurring losses of US$26.8 billion annually worldwide [1]. There have been some explanatory hypotheses for how ecological adaptions and mechanistic changes that cause species to succeed, spread, and outbreak in new habitats [2–4], such as the diversity resistance hypothesis [5], empty niche hypothesis [6], enemy release hypothesis [7], and symbiotic invasion hypothesis [8, 9]. However, the invasion ability of invasive species resulting from endogenous mechanisms has been less studied [10–12]. The scarcity of knowledge about the genomic basis of invasive species prevents further understanding of underlying changes in invasion ability. The rapid development and declining costs of sequencing have allowed its application to a number of invasive species, such as the Asian long-horned beetle *Anoplophora glabripennis* Motsch [13]., the fall webworm *Hyphantria cunea* Drury [14], and the codling moth *Cydia pomonella* Linnaeus [15], to better understand the invasion process.

Coleoptera (beetles) is the most species-rich order of insects with over 400,000 described species [16], and some are responsible for billions of dollars of losses annually [10, 13, 17]. Since the red flour beetle *Tribolium castaneum* Herbst (Tenebrionoidea) was first sequenced [18], the mountain pine beetle (MPB) *Dendroctonus ponderosae* Hopkins (Curculionoidea) [19], the Asian longhorned beetle *A. glabripennis* (Cerambycoidea) [13], and Colorado potato beetle *Leptinotarsa decemlineata* Say (Chrysomeloidea) [20] have been sequenced. The superfamily Curculionoidea diverged from Tenebrionoidea 236 million years ago (Mya) [21], and contains over 60,000 described species, including many destructive wood-boring pests, such as bark beetles. MPB is a primary tree-killing *Dendroctonus* bark beetle in North America and has long recorded history of major outbreaks [22]. In contrast, the red turpentine beetle (RTB), *Dendroctonus valens* LeConte, is a secondary bark beetle that does not commonly cause tree mortality in its native range in North America, but since its introduction into China in the 1980s, it has caused substantial tree death in China [23]. Keeling et al. reported a draft genome of MPB and explored P450s, glutathione S-transferase, and plant cell wall-degrading enzyme gene families important to utilizing nutrient-poor hosts as *Pinus* phloem, providing valuable information for other bark beetles [19]. However, specific genomic information on RTB is needed, and obtaining this information in both its native and introduced regions can increase our understanding of how a minor or secondary pest in its native range can have heightened significance in introduced habitats.

In its native range of North America, which extends from Nova Scotia (Canada) west to California (USA) and south to Honduras [24], RTB is a secondary pest that rarely attacks healthy pine trees and frequently attacks trees under stress, often caused by root diseases, fire, and mechanical wounding [25, 26]. However, RTB is a major tree-killing species in China and has killed over 10 million Yousong since its first outbreak in China [17, 23]. RTB mainly infests Yousong (resin pine) *Pinus tabuliformis* Carr [27].. Yousong is a native pine in China, and its genome reveals genes for terpenoid biosynthesis have significantly expanded [28]. Terpenoid metabolism plays vital roles in defending against pests and pathogens as well as adapting to environmental conditions in conifers [29], which may impose crucial pressure on RTB when it first invaded China. Previous studies showed invasive populations of RTB in China shared haplotypes with RTB populations within its native range of the Pacific Northwest (PNW) [30] and invasive populations of RTB in China had higher haplotype diversity than that in the PNW [31], suggesting some degree of genetic bottleneck in the early stages after the introduction of RTB into China followed by a relatively rapid population establishment. Behavioral characteristics of RTB were initially presumed to be similar in North America and China [27]. However, the Chinese populations of RTB have distinct adaptations, which likely have developed in response to novel conditions in its new ecosystem [17]. The most striking characteristic of Chinese populations of RTB is their ability to colonize, kill, and reproduce in apparently healthy *P. tabuliformis*, resulting in multiple outbreaks unlike its native region [17, 27]. Studies in the USA demonstrate that RTB are attracted by host volatiles released during harvesting operations [32]. However, Chinese populations of RTB use specific host monoterpenes ratios to colonize healthy hosts [33, 34]. More importantly, Chinese populations of RTB aggregate in much larger densities than that in North America [25, 26], which putatively overcomes resin pine defenses. The aggregation is regulated by aggregation pheromone frontalin and the anti-aggregation pheromone *exo*-brevicomin [35, 36].

To understand how a secondary pest in its native range can become a primary pest in a new region, we investigated the genetic basis of RTB using multiple sequencing technologies. Additionally, we target genomic variations between native and invaded populations by genome resequencing, and explored possible adaptation capacities of RTB to new environments during the course of invasion. We report high quality chromosome-level genome of RTB and describe several highlights including gene family expansion and positively selected genes that might be involved in environmental adaption, the identification of X-chromosome, the origin of RTB population that invaded China, and genes showing signals of selective sweep in Chinese population contributing to evolutionary adaption in its new habitat.

## Results

### Chromosome-scale genome assembly of RTB

**Fig. 1:**
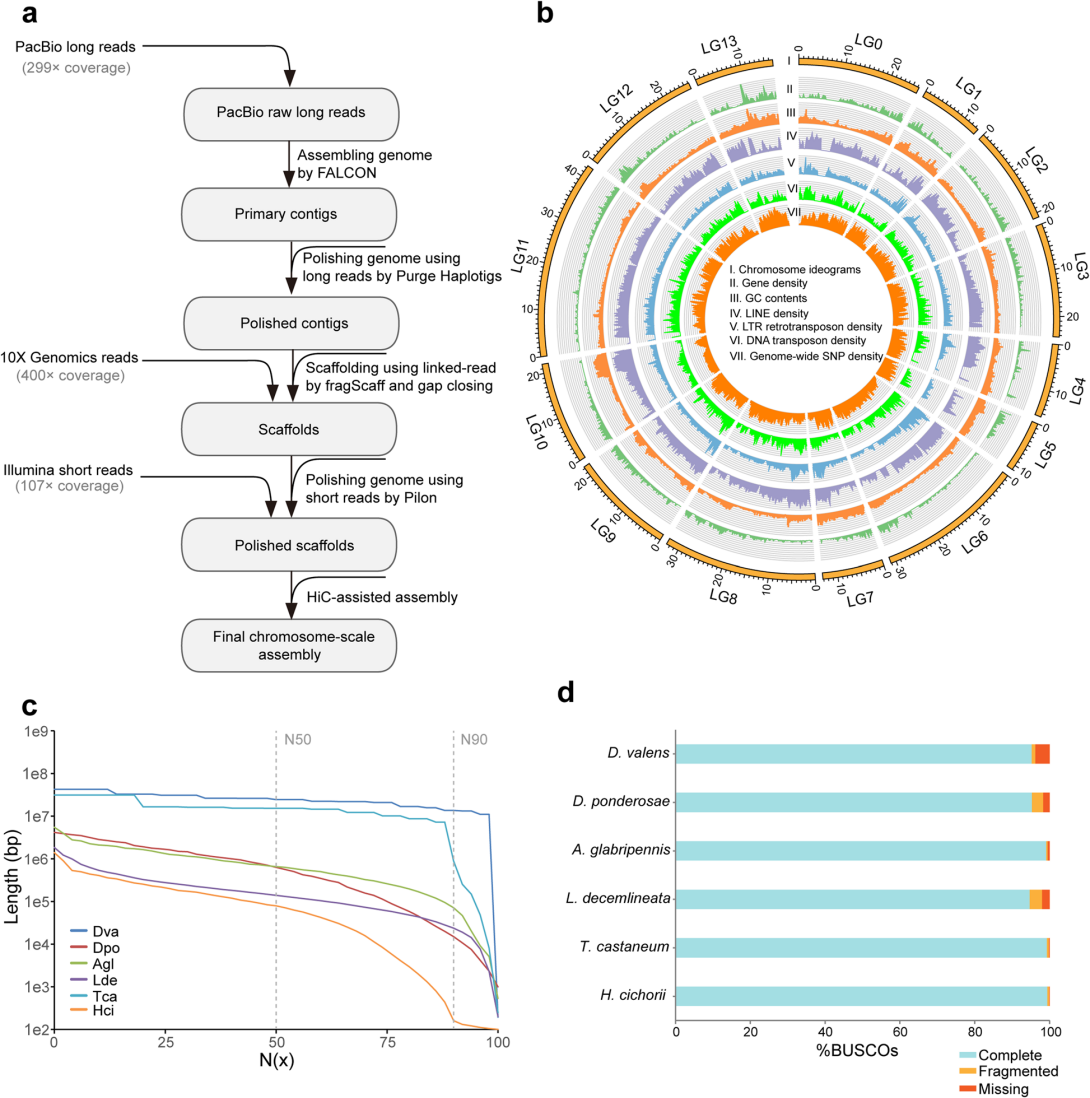
Scheme of genome assembly and genomic features of the red turpentine beetle, *Dendroctonus valens*. **a** Sequencing scheme and genome assembly strategy adopted for *D. valens* genome sequencing. A strategy combining long-read PacBio sequencing, short-read Illumina sequencing, and HiC sequencing schemes was employed for generating a high-quality and complete genome assembly for *D. valens*. **b** Circular plot representation of the characteristics of the red turpentine beetle genome. From the outer to the inner panel, these genome tracks represent the following: I. Chromosome ideograms; II. Density of protein-coding genes; III. Density of G+C content; IV. Density of long interspersed nuclear elements (LINEs); V. Density of long terminal repeats retrotransposons (LTR-RTs); VI. Density of DNA transposons; and VII. Density of genome-wide single nucleotide polymorphism (SNP) sites, respectively. **c** Line plot showing the distribution of N(x) metrics in *D. valens* and other five Coleoptera insect species. The vertical dotted lines indicate the positions of N50 and N90 size, respectively. **d** BUSCO evaluation result for genome assemblies of six Coleoptera insects

A total of 300 × single molecule real-time (SMRT) long reads, 107 × coverage Illumina paired-end reads, and 400 × 10X Genomics reads were generated by PacBio Sequel and Illumina HiSeq X Ten sequencing platform, respectively (Fig. 1a; Additional file 1: Table S1). Initially, RTB genome was assembled into 1144 contigs spanning 320.96 Mb with a contig N50 size of 985.47 kb (Table 1; Additional file 1: Table S2). Furthermore, these contigs were significantly improved to generate 923 scaffolds with a scaffold N50 size of 1.66 Mb (Table 1; Additional file 1: Table S2) using 10X Genomics data. The assembled genome size was 322.41 Mb, close to the estimated genome size of 372.97 Mb (Additional file 2: Figure S1). Finally, Hi-C data were employed for the anchoring, ordering, and orientation of these scaffolds, yielding 14 linkage groups (LG), harboring >98% of assembled sequences with a N50 size of 24.36 Mb (Table 1; Fig. 1b, c; Additional file 2: Figure S2). Benchmarking Universal Single-Copy Orthologs (BUSCOs) assessment showed that RTB genome assembly covered 96.1% of complete BUSCOs (Fig. 1d; Additional file 1: Table S3).

**Table 1:** Comparison of genome assemblies in six Coleoptera species

Features	***Dendroctonus valens***	***Dendroctonus ponderosae***	***Tribolium castaneum***	***Anoplophora glabripennis***	***Hycleus cichorii***	***Leptinotarsa decemlineata***
Genome size (Mb)	322.41	252.85	165.94	707.71	111.71	641.99
Karyotype	12+XY	11+XY	9+XY	-	-	-
Number of contigs	1144	59,583	7059	26,749	16,072	45,556
Number of scaffolds	923	8188	2149	10,473	13,823	26,908
Number of assembled chromosomes	14	NA	10	NA	NA	NA
Genome assembly quality						
Contig N50 (kb)	985.47	7.45	73.05	80.49	86.94	46.6
Scaffold N50 (Mb)	1.66	0.63	4.46	0.68	0.079	0.14
Linkage group N50 (Mb)	24.4	NA	15.3	NA	NA	NA
BUSCO genes (%)	95.2	95.3	99.3	99.1	99.4	94.7
Genomic features						
Repeat (%)	45.22	21.12	28.9	62.1	31.93	38.63
G + C (%)	36.7	28.7	35.19	33.4	32.3	35.6
Gene annotation						
Number of genes	13,751	14,342	16,590	22,035	13,813	18,644

Repeat annotation showed that repetitive elements occupied 45.22% of genome sequence. Among them, long interspersed nuclear elements (LINE) (27.93%), long terminal repeat retrotransposons (LTR-RT) (12.19%), and DNA transposons (6.81%) represented the top three most abundant repeat types, followed by tandem repeat and short interspersed nuclear elements (SINE) (Additional file 1: Table S4).

Based on automatic gene prediction method, a total of 13,751 consensus protein-coding gene models (official gene set, OGS) were identified in the genome. Furthermore, we manually annotated chemosensory, detoxification, and metabolism-related gene families that are important for RTB adaptation to its environment (Additional file 1: Table S5). Subsequently, the OGS was subjected to functional annotation against non-redundant protein database (NR), SwissProt, InterPro, Kyoto Encyclopedia of Genes and Genomes (KEGG), Gene Ontology (GO), and protein family (Pfam) database, showing that 99.6% of protein-coding genes could be functionally annotated (Additional file 1: Table S6).

### Rapidly expanded and positively selected genes promoting RTB adaptations to environmental stress

**Fig. 2:**
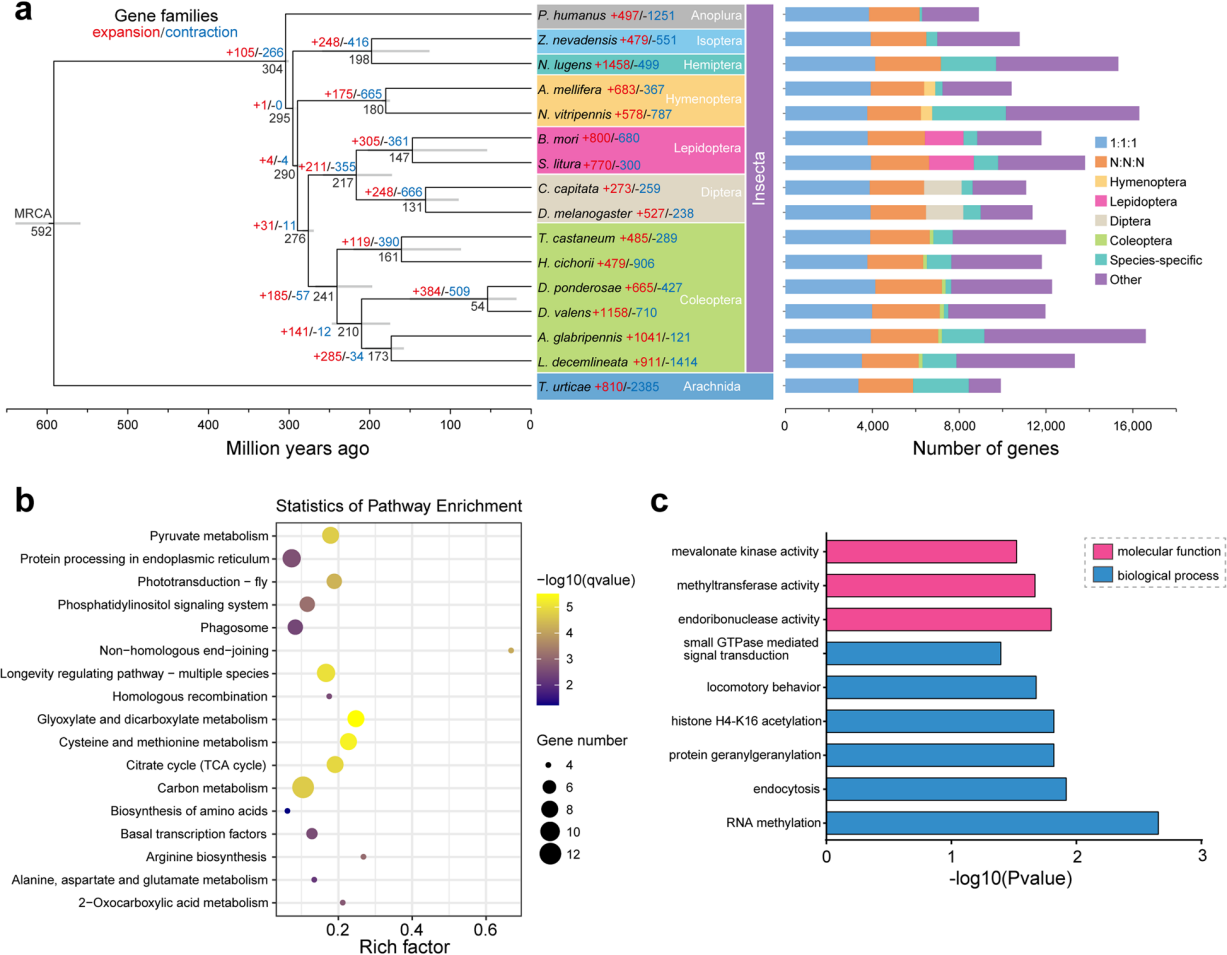
Gene family evolutionary analysis and positive selection analysis in the red turpentine beetle. **a** Phylogenetic tree and orthologs in the red turpentine beetle and 15 other arthropod species. The intra- and interspecies orthologs and paralogs were assigned using the OrthoMCL. The phylogenetic tree was inferred from 952 strict single-copy orthologous genes among 16 arthropod species using the RAxML software with the maximum likelihood method employing LG+G+I model and 500 bootstrap replications. All the nodes have at least 90% bootstrap support. The divergence time was estimated using the MCMCtree program with several calibration time points adopted from the TimeTree database (https://www.timetree.org). Numbers on internal nodes indicate divergence time, and gray bars represent error ranges. The phylogenetic tree was rooted on the two-spotted spider mite *Tetranychus urticae*. MRCA is the abbreviation of most recent common ancestor. 1:1:1 represents universal single-copy genes across these species, allowing for absence in two beetles and duplication in one species. N:N:N stands for universal multiple-copy genes across these species. The genes that were specific in Hymenoptera, Lepidoptera, Diptera, and Coleoptera were also shown in the figure in corresponding colors. Meanwhile, lineage-specific genes were also displayed for each species. Other denotes genes that do not belong to these groups in these species. Expansion and contraction analysis of gene families was performed using CAFÉ 3. The number of expanded (red) and contracted (blue) gene families are marked in the corresponding branch. **b** KEGG pathway enrichment analysis result of rapidly expanded gene families. Gene families with *P* < 0.05 were defined as rapidly evolving families. Pathway with *q*value < 0.05 was considered significantly enriched. **c** Gene ontology (GO) enrichment analysis result of positively selected genes in *D. valens*. GO categories with *P* < 0.05 were considered significantly enriched

To determine the phylogenetic relationship of RTB to other insect species, 952 strict single-copy orthologs were used to infer the maximum-likelihood species tree using RAxML. Results showed that RTB clustered with five other Coleoptera species, such as the red flour beetle (*Tribolium castaneum*), the blister beetle (*Hycleus Cichorii*), MPB (*Dendroctonus ponderosae*), the Asian longhorned beetle (*Anoplophora glabripennis*), and the Colorado potato beetle (*Leptinotarsa decemlineata*) and exhibited the closest distance to MPB (Fig. 2a). Divergence time analysis showed that Curculionoidea diverged from Tenebrionoidea about 240 Mya and RTB diverged with MPB from the most recent common ancestor approximately 40 Mya (Fig. 2a). Orthology assignment showed that a total of 7110 genes were maintained as orthologs across almost all studied species. Among them, 3989 represented 1:1:1 orthologs, and 3121 represented N: N: N orthologs. Additionally, *A. glabripennis* possessed the most species-specific genes in Coleoptera as reported previously (Fig. 2a).

To determine the change of gene family members in RTB during evolution, CAFÉ analysis was performed to identify expanded (gene gain) or contracted (gene loss) families. Results showed all 13,751 genes in RTB were assigned onto 6194 gene families in TreeFam database. The majority of the gene families (5507, 81.94~88.90%) were commonly shared by four Coleoptera species, and only a small fraction of gene families (1.04~3.04%) was unique among them (Additional file 1: Table S7, Additional file 2: Figure S3). According to the CAFÉ analysis, we found that 1158 and 710 gene families were expanded and contracted in RTB, respectively (Fig. 2a). Moreover, a total of 63 rapidly evolving gene families (*P-*value < 0.05) (34 expanded and 29 contracted families) (Additional file 1: Tables S8-9) were found in RTB. Functional enrichment showed that these rapidly expanded gene families were associated with multiple metabolism-related pathways, such as carbon metabolism, pyruvate metabolism, citrate cycle, and metabolism of multiple amino acids (Additional file 1: Table S8) (Fig. 2b). Especially, the expanded gene family in carbon metabolism included 5 carbonyl reductase genes. Besides, the rapidly expanded families were also involved in phototransduction, phosphatidylinositol signaling system, phagosome, protein processing in endoplasmic reticulum, and longevity regulating pathway (Additional file 1: Table S8) (Fig. 2b).

Further, we performed positive selection analysis on the single-copy orthologs among six Coleoptera species. A total of 193 genes were identified to undergo selection pressure in RTB, thus designated as positively selected genes (PSGs) (Additional file 1: Table S10). Gene ontology (GO) enrichment analysis (Additional file 1: Table S11) revealed that PSGs are enriched for many biological processes, including RNA methylation (*P* = 0.002), endocytosis (*P* = 0.012), protein geranylgeranylation (*P* = 0.015), histone H4-K16 acetylation (*P* = 0.015), locomotory behavior (*P* = 0.021), and small GTPase mediated signal transduction (*P* = 0.040) (Fig. 2c). Additionally, multiple molecular functions such as endoribonuclease activity (*P* = 0.016), methyltransferase activity (*P* = 0.021), and mevalonate kinase activity (*P* = 0.030) were closely associated with the PSGs in RTB (Fig. 2c).

### Chromosome fission and X chromosome identification in RTB

**Fig. 3:**
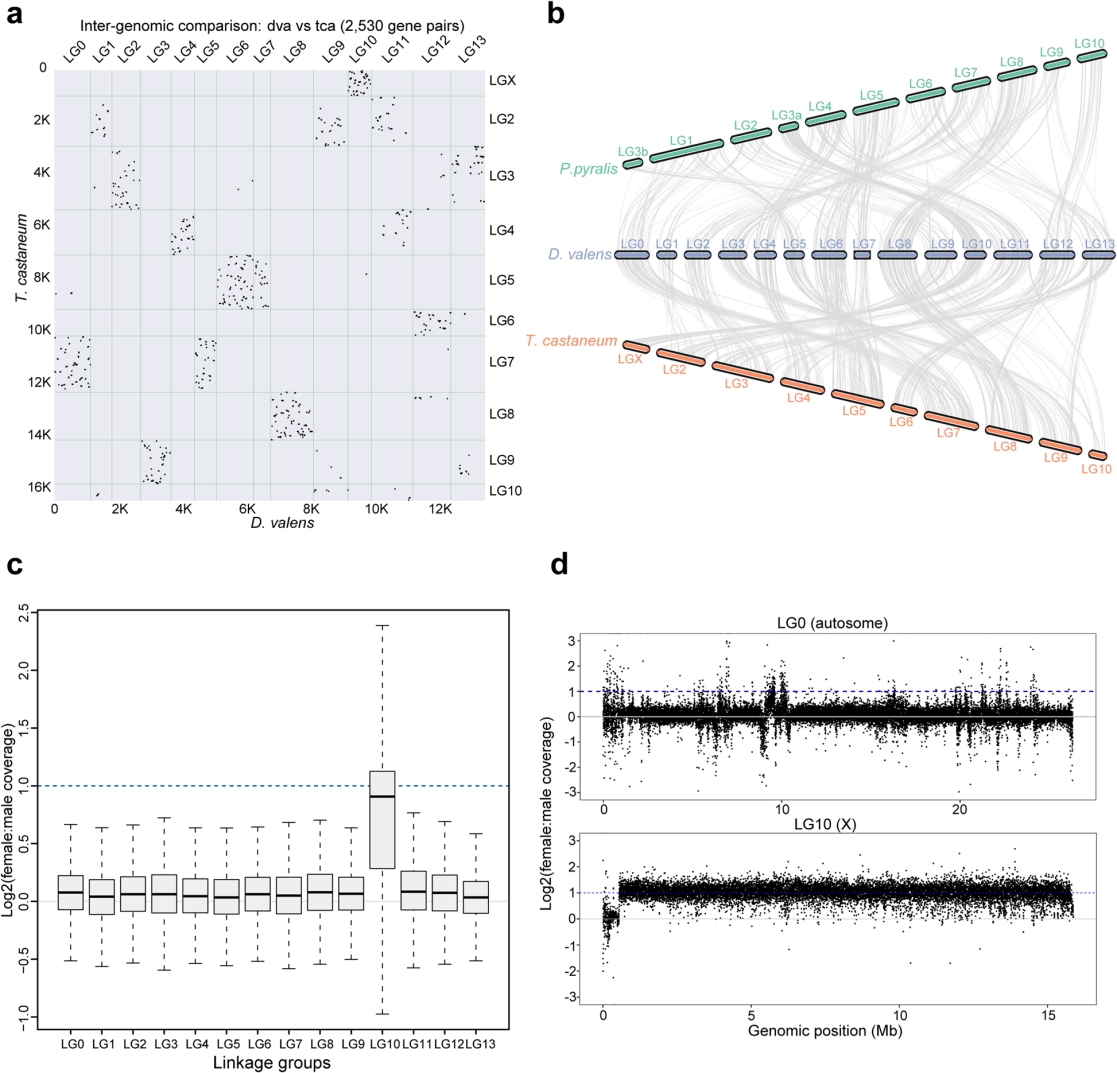
Synteny analysis across three Coleoptera insects and identification of sex chromosomes in *Dendroctonus valens*. The potential sex chromosomes were identified based on the distribution pattern of female to male (F:M) coverage ratios along 14 assembled linkage groups in resequencing individuals from Shanxi population. **a** Dot plot representation of the syntenic relationship between *D. valens* chromosome pseudo-molecules and *Tribolium castaneum* linkage groups. Notably, *D. valens* LG10 showed a strong synteny relationship with *T. castaneum* LGX, suggesting LG10 might be the putative X chromosome in *D. valens*. **b** Genome-wide synteny relationship between *D. valens* and two Coleoptera insects, *T. castaneum* and *P. pyralis*. Genome-wide synteny analysis was performed using the MCScan pipeline of JCVI utility libraries. **c** Boxplot showing the distribution of F:M coverage ratios in non-overlapping intervals of 1 kb on each chromosome in RTB. Herein, we employed the resequencing data from Shanxi population for the calculation of F:M coverage ratios. Theoretically, the female coverage for autosomes were close to 1, while the ratio for chromosome X was approximately 2. Thus, LG10 was determined as the chromosome X in *D. valens*. **d** Scatter plot showing the distribution of F:M coverage ratios in non-overlapping intervals of 1 kb along an autosome (LG0, top panel) and the putative chromosome X (LG10, bottom panel)

We performed a genome-wide synteny analysis between RTB and the Coleoptera model insect *T. castaneum*. On the whole, almost all the linkage groups of RTB showed collinearity against those of *T. castaneum* (Fig. 3a). Regarding the diversification of karyotypes in Coleoptera, we analyzed the possible chromosome fusion and fission events occurring in *D. valens*. Notably, the synteny relationship of *T. castaneum* LG5 against RTB LG6 and LG7 might indicate a fission event of chromosome occurring in *D. valens*. Similarly, LG0 and LG5 in RTB might represent another fission event.

Interestingly, the results showed that LG10 in RTB had a strong syntenic relationship with LGX in *T. castaneum* (Fig. 3a). A similar result was observed in the syntenic plot for three Coleoptera species (*D. valens*, *T. castaneum*, and *P. pyralis*) (Fig. 3b). LG10 in RTB also exhibited unique synteny with the *P. pyralis* LG3a which corresponds to chromosome X. During the review process of this manuscript, a chromosome-level genome assembly of *D. ponderosae* was reported [37]. We also performed a genome-wide synteny analysis between *D. valens* and *D. ponderosae* (Additional file 2: Figure S4). Interestingly, *D. ponderosae* showed a stronger syntenic relationship with *D. valens* than did *T. castaneum* (Additional file 2: Figure S4a), consistent with the phylogenetic distance between them. Notably, the ultra-long neo-X chromosome chr1 in MPB was formed by the fusion of four LGs of RTB (i.e., LG1, LG4, LG10, and LG11), while Dpochr9 was fused with Dpochr12 to form LG13 in RTB (Additional file 2: Figure S4b). To further determine X chromosome-related linkage groups in RTB, genome resequencing reads were employed. The female to male (F:M) coverage ratios on autosomes were close to 1.0. In contrast, the F:M coverage ratios on LG10 were twice than those for autosomes, consistent with the distribution of sex chromosome in males and females (Fig. 3c). Furthermore, the scatter plots of F:M coverage showed the same trend (Fig. 3d). Altogether, the linkage group 10 was determined as the X chromosome.

### Invasion and spread pattern of RTB revealed by population genomics analysis

To reveal the genetic variants in RTB across different geographic populations, a total of 107 individual samples were collected from the native region in North America and the invaded country China (Additional file 1: Table S12), which generated 683 Gb clean PE150 paired-end data (approximately 17.32× average sequencing depth) by Illumina HiSeq X Ten platform (Additional file 1: Table S13). High-quality single-nucleotide polymorphism (SNP) sites were identified using the Genome Analysis Toolkit (GATK) pipeline.

**Fig. 4:**
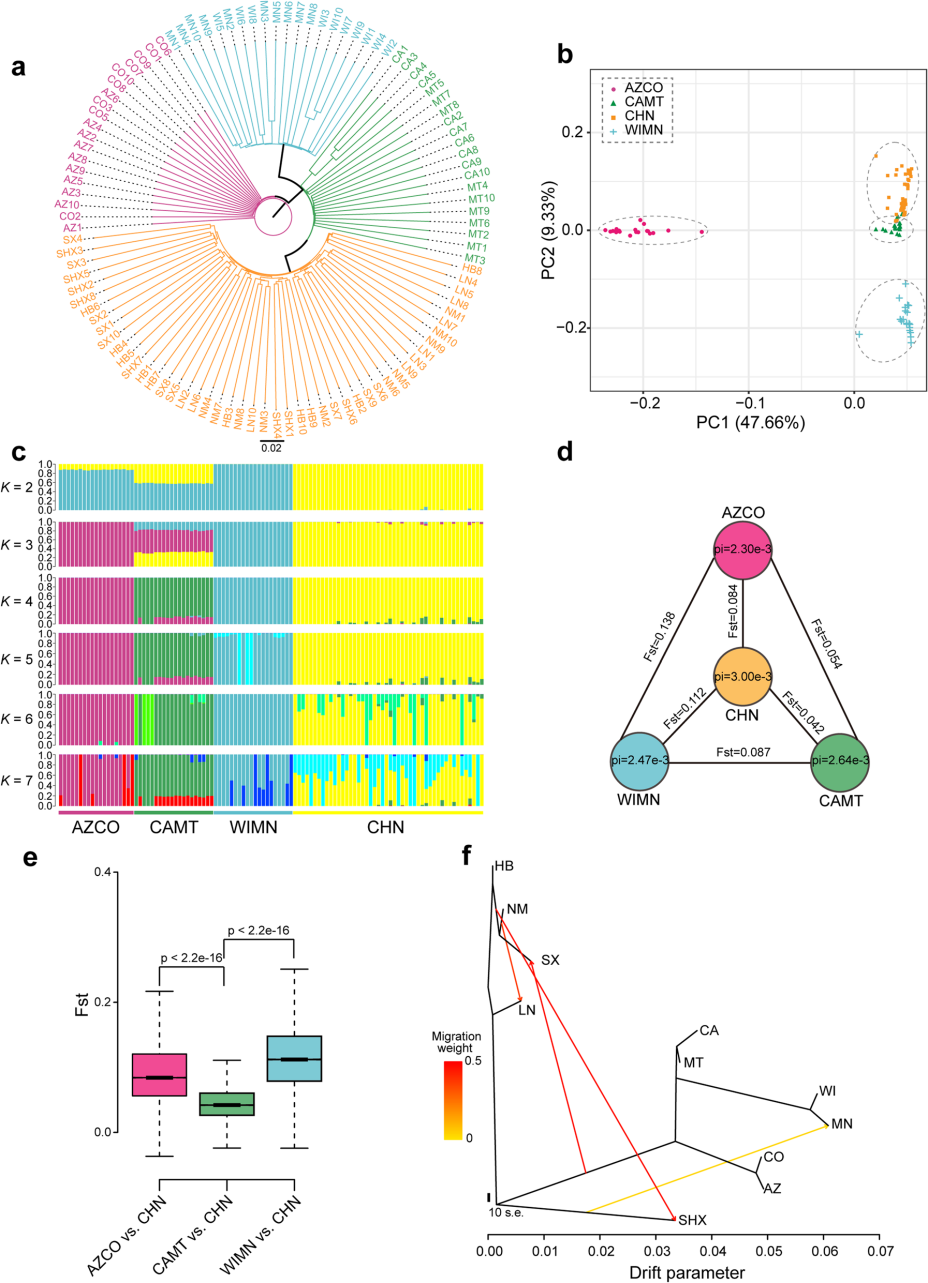
Population genomic analysis of red turpentine beetles in the original and invaded regions. **a** Neighbor-joining phylogeny of all *Dendroctonus valens* individuals constructed from genome-wide SNP data using the PHYLIP software based on distance data. Abbreviations for sampling sites are as follows: AZ, Arizona; CO, Colorado; CA, California; MT, Montana; WI, Wisconsin; MN, Minnesota; LN, Liaoning; NM, Inner Mongolia; HB, Hebei; SX, Shanxi; SHX, Shaanxi. **b** Principal component analysis (PCA) of resequencing individuals based on the first two principal components. American population was divided into three subpopulations based on top two components PC1 and PC2. PC1 could separate WIMN from China and other two American subpopulations, while the second axis could distinguish three American subpopulations. **c** Population genetic structure of individuals sampled from geographic locations in China and North America revealed by the ADMIXTURE software. The cross-validation error was the lowest when *K* = 3, suggesting three ancestral populations were best supported by the data. Notably, American population could be divided into three sub-groups based on the population structure. **d** Population genetic metrics in China and three American subpopulations. Genome-wide median pairwise fixation index (*F*_ST_) was calculated for three contrast groups between China and American subpopulations, and genome-wide median nucleotide diversity (Pi) was computed for each population. **e** Genome-wide distribution of *F*_*ST*_ in three contrast groups between China population and three American subpopulations. Notably, CAMT vs. CHN group displayed the smallest variation among three contrast groups. Wilcoxon rank sum test was used for determination of statistical significance between groups. **f** Gene flow analysis across different geographical subpopulations in native and invaded regions. The possible gene flow events across geographic subpopulations were inferred using TreeMix when assuming the occurrence of four gene flow events

The 60 RTB samples in North America were grouped into three distinct clusters (AZCO, WIMN, and CAMT) based on the phylogeny reconstructed from SNP data, principal component analysis (PCA) and population structure analysis (Fig. 4a–c). The results showed AZCO and WIMN represent two pure subpopulations, while the CAMT subpopulation was highly hybridized (*K* = 3; Fig. 4c), representing the admixture of three ancestral populations. In contrast, 47 RTB from China were clustered together (Fig. 4a–c) and have low nucleotide diversity (Pi = 3.0e^−3^; Fig. 4d). Only slight levels of admixture with AZCO and WIMN ancestral populations were observed in part of the China population. By contrast, the CAMT population was close to the China population (Fig. 4b), and approximately one third of the composition of population in CAMT population was derived from the China population (*K* = 3; Fig. 4c).

To further determine the genetic distance between the China population and the American subpopulations, nucleotide diversity (Pi) and pairwise fixation index (*F*_ST_) was computed for four populations and three contrast groups, respectively. Notably, WIMN vs. China (CHN) group showed the highest *F*_ST_ value (median: 0.112), followed by AZCO vs. CHN group (median 0.084), and CAMT vs. CHN had the lowest *F*_ST_ value (median 0.042) (Fig. 4d). Additionally, *F*_ST_ values of CAMT vs. CHN were significantly lower than those of the other two groups (Wilcoxon rank sum test, *P* < 2.2e^−16^) (Fig. 4e), suggesting the smallest differentiation between CHN and CAMT populations. This coincides with the assumption that RTB that invaded China might be derived from the Pacific Northwest of the USA as reported in Cognato et al. [30]. TreeMix analysis was conducted to reveal possible gene flow events among geographic populations. Interestingly, a gene flow event was observed from the ancestry of American populations to Shanxi (SX) population with a high migration weight when assuming four gene flow events (Fig. 4f; Additional file 2: Figure S5), further supporting the hypothesis of invasion route of RTB [30].

### Strong selection contributing to new environmental adaptation of RTB in China population

**Fig. 5:**
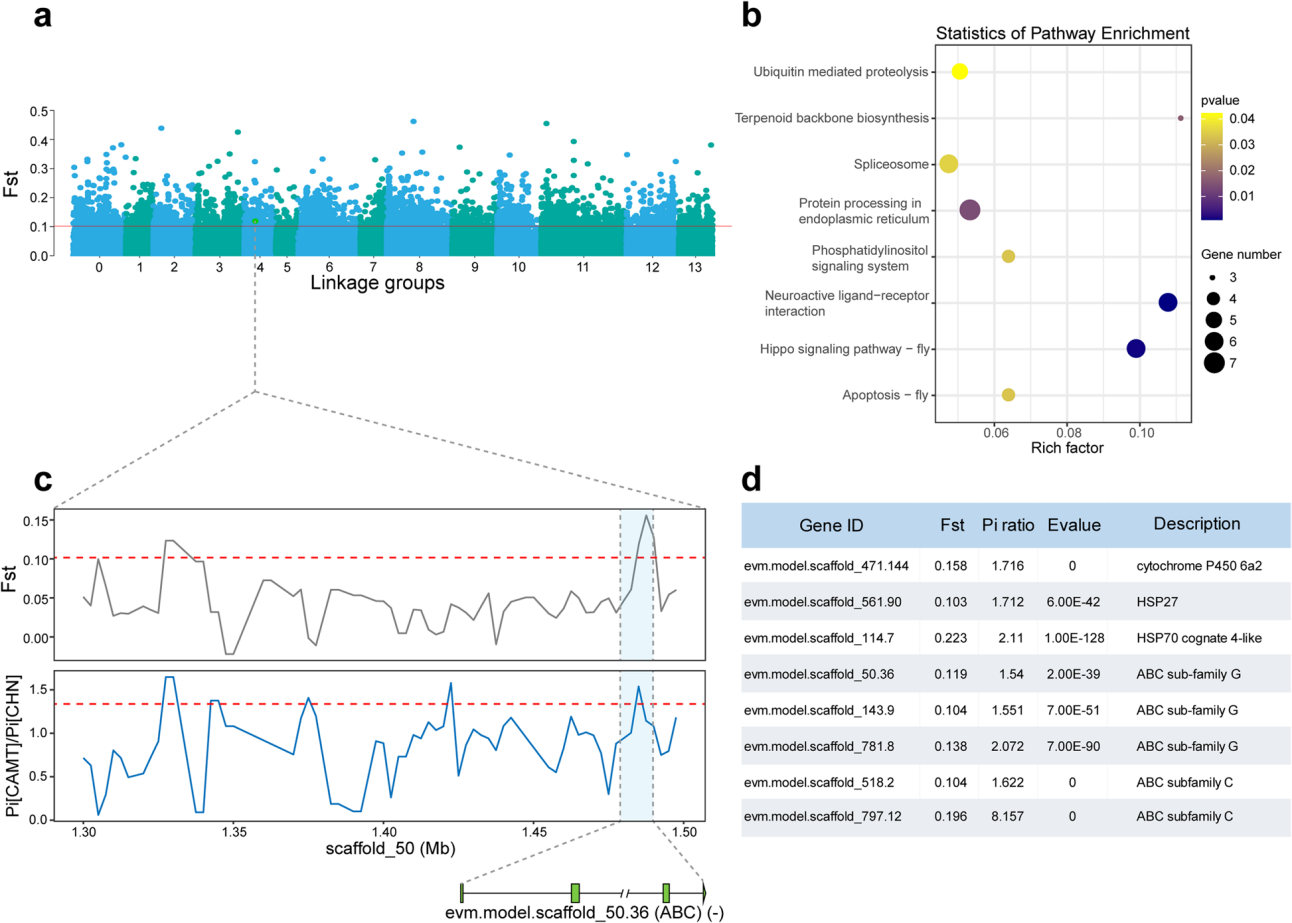
Genome-wide scan signals of selective sweeps in red turpentine beetle (RTB) populations. **a** Manhattan plot showing the distribution of *F*_ST_ across fourteen linkage groups of *D. valens* in CAMT vs. CHN group. Genomic regions that showed strong signal of positive selection were determined as the outliers. The red horizontal line indicates the cutoff of top 95% quantile in the genome-wide scale. Due to the significant divergence in American populations, selective sweep analyses were separately performed for three contrast groups, including AZCO vs. CHN, CAMT vs. CHN, and WIMN vs. CHN. The genomic regions corresponding to windows with *F*_ST_ values and Pi ratios located in top 5% quantile of the whole-genome metrics were considered as positively selected regions. **b** KEGG pathway enrichment analysis of protein-coding genes that underwent selective sweep in the China population compared to CAMT population. Pathways with *P* < 0.05 were considered significantly overrepresented. **c** Distribution of *F*_ST_ values and Pi ratios around the genomic regions that were positively selected in the China population. The red dotted line represents the outlier cutoff of *F*_ST_ values. The peak region in light blue background corresponds to a gene under selection in China, an ATP-binding cassette transporter (ABC), whose exon-intron structure is also shown below. **d** List of eight representative genes associated with stress resistance and environmental adaptation that showed strong signatures of selection in the China population compared to CAMT population. One cytochrome P450 (CYP450), two heat shock proteins (HSPs), and five ABCs were observed to be strongly selected in the China population

To identify genomic regions in RTB showing signatures of selection during adaptation, selective sweep analyses were conducted across geographic populations in native and invaded regions as described previously [38]. Considering that the American population was divided into three subpopulations (Fig. 4a), selective sweep analysis was conducted for three contrast groups (including AZCO vs. CHN, CAMT vs. CHN, and WIMN vs. CHN), respectively. Overall, we observed more genomic regions showing significant signals of selection in China population than in three American subpopulations (Additional file 2: Figure S6). Herein, we placed an emphasis on the selective sweep analysis between CAMT and China population (Fig. 5a). A total of 377 genes showed significant signatures of positive selection in the Chinese population (Additional file 1: Table S14).

Notably, positively selected genes were strongly associated with terpenoid backbone biosynthesis, protein processing in endoplasmic reticulum, neuroactive ligand-receptor interaction, and Hippo signaling pathway (Fig. 5b). We also identified some genes associated with environmental adaptation and stress resistance that are significantly selected in China population. For example, an ATP-binding cassette (ABC) transporter gene, which might be associated with multiple anti-oxidant response and immune response, was found to be under selection in China (Fig. 5c). As shown in Fig. 5d, eight representative genes that were involved in detoxification and stress response were strongly selected in China population, including a cytochrome P450 gene, two genes encoding heat shock proteins, and five ABC transporter genes, implying the rapid response of the bark beetle to biotic or abiotic stress.

## Discussion

Chromosome-level genome assemblies have been obtained for several Coleoptera through combining PacBio or Nanopore long-read sequencing and Hi-C technology [39–41]. Such reference genome assemblies of model organisms are continuously updated with the development of sequencing technology [42–45], to facilitate further functional analysis. The first chromosome-level reference genome of bark beetles was assembled in this study, which also showed significantly better quality in terms of N50 and N90 metrics compared to five others representative Coleoptera species [13, 18–20, 46]. Nonetheless, there exists a difference between the genome size estimated by k-mer analysis (372.97 Mb) and the final assembled genome size (322.41 Mb). One possible explanation might be that the bias was caused by the high repeat content and high heterozygosity rate of the RTB genome. Due to the existence of long repeats in the *D. valens* genome, PacBio reads could not span such repeat regions successfully, thus resulting in the bias in genome size. Similarly, the difference between the estimated genome size and the assembled genome size was also reported in genome assemblies of other insect species published previously, e.g., 204 Mb (estimated) vs. 166 Mb (assembled) in *T. castaneum* [18], 269.87 Mb (estimated) vs. 111.71 Mb (assembled) in *H. cichorii* [46], 6.3 Gb (estimated) vs. 6.5 Gb (assembled) in *Locusta migratoria* [47], and 940 Mb (estimated) vs. 902 Mb (assembled) in *Aquatica lateralis* [39]. Using this high-quality chromosome-level genome assembly of RTB, we have identified much more repetitive elements than in four of five other Coleoptera insects’ genomes previously published [18–20, 46], enabling a comprehensive analysis in any kind of repeat sequence content as well as chromosome fission and X chromosome identification. The high-quality assembly allows fine annotation of gene models, facilitating evolutionary and functional analysis.

The high-quality assembly genome enabled a comprehensive analysis of TEs which play important roles in driving genome evolution in eukaryotes [48]. In this study, repetitive elements occupied 45.22% of genome sequence in *D. valens*, which is substantially higher than the percentage of repetitive elements in *D. ponderosae* (21.12%) [19], *T. castaneum* (28.90%) [18], *H. cichorii* (31.93%) [46], and *L. decemlineata* (38.63%) [20], and lower than that in *A. glabripennis* (62.1%) [13] (Table 1). It should be noted that although we compared TE content among different Coleoptera insects, the methods used for TE annotation are different for each species, this might create a bias on the percentage of total TE copies. TEs are powerful facilitators of evolution to introduce small adaptive changes within a lineage, promoting the responses and adaptation of organisms to changing environment [49, 50]. The high content of TEs in the *D. valens* genome may enhance its environmental adaptation and substantially contribute to its invasiveness. It should be noted that multiple high-quality Coleoptera insect genomes have been reported in recent years, including the firefly *Photinus pyralis* [39], the Easter Egg Weevil *Pachyrhynchus sulphureomaculatus* [51], the carabid beetle *Nebria riversi* [52], and the ladybird beetles *Propylea japonica* [40] and *Harmonia axyridis* [41]. A systematic comparison of genomes across these insect species will provide insights into TE content, the variation of genome size, and evolutionary relationship in the vast Coleoptera phylogeny in the future.

Whether single genes or whole genomes [53–55], duplications relax the selection on any one gene or gene component that remains duplicated. Notably, genetic evolution is particularly important, especially when examining different duplicate gene retention patterns. In this study, these rapidly expanded gene families in RTB were associated with multiple metabolism-related pathways (Fig. 2b). These genes not only are expected to confer better use of nitrogen-poor host pine trees and adaptations to environmental stress [56–58], but also are involved in detoxification processes of endogenous and xenobiotic reactive carbonyl compounds [59, 60]. As host *Pinus* spp have relatively low nitrogen content [32] and high concentration of carbonyl compounds [61, 62], we postulate that these expanded carbonyl reductase genes contribute to adaption to low-nutrient hosts. Additionally, the genes involved in geranylgeranylation and mevalonate kinase activity might be related to production of pheromone frontalin, which is de novo synthesized through the mevalonate pathway in *Dendroctonus*, helping RTB aggregate on host *P. tabuliformis* [63]. Together, these indicate that the RTB might have enhanced adaptation to environmental stress and elevated metabolic capacity [64].

We used genome resequencing data of geographic populations in native and invaded regions to identify the genes that exhibited significant signatures of positive selection in China population. Interestingly, the enrichment of selected genes in terpenoid backbone biosynthesis might indicate a difference in the biosynthesis of hormone and pheromone between China and CAMT subpopulations. In its native range of North America, RTB typically infest weakened pine trees caused by root diseases, fire, and mechanical wounding [25, 26], and because these trees are less resistant to bark beetle attack, RTB may not need to positively select terpenoid backbone biosynthesis genes in its native environment. However, Chinese populations of RTB can infest healthy standing Yousong *P. tabuliformis* [17]. These trees have substantial resin and the tree’s genome reveals genes for terpenoid biosynthesis that are significantly expanded [28], which may have imposed pressure on RTB when it first invaded China. Differences in genes may attribute to pheromone frontalin production regulating RTB mass attack to overcome host resistance in China relative to North America [35]. Besides, positive selection on genes such as cytochrome P450 gene, heat shock protein, and ABC transporter genes, in China population, may be involved in detoxification and stress response (Fig. 5d), implying the rapid response of the bark beetle to biotic or abiotic stress [19, 65–69]. We postulate that after the invasion of RTB into China, several functional genes related to detoxification and stress response were strongly selected, which not only enhanced their resistance to temperature and rapid pesticide adaptation ability, but also increased RTB survival to adapt to novel host *P. tabuliformis* in China.

The invasion and spread pattern of invasive species have long been the focus of attention. Population-based resequencing has been successfully applied to elucidate the origin and spread of populations in a number of species [70, 71]. Our results show that RTB samples in their native ranges were clustered into three subpopulations (Fig. 4a–c), indicating significant divergence in North America, which correspond to previous research in RTB [72], MPB [73], and *Dendroctonus rufipennis* Kirby [74].

The natural history of *Dendroctonus* is closely linked to its conifer hosts in North America [75, 76]. Host *Pinus* spp. underwent a north to south dispersion along the principal mountain ranges of North America, such as the Rocky Mountains [76–78] and previous studies suggest the diversity center of *Dendroctonus* is in the northern region of North America [76]. According to this hypothesis and the current results (Fig. 4a–d), RTB likely originated in Canada and extended southward in two branches, a western route that ultimately arrived in Mexico along the Rocky Mountains and an eastern route via Minnesota and Wisconsin that reached New York, to yield the current wide distribution throughout North and Central America [75]. The Central Great Plains of the USA, which are about 800 km wide, may block genetic exchange between the AZCO and WIMN populations, thus contributing to the formation of two distinct populations. However, there are no equally extensive natural barriers between population CAMT and AZCO. Future studies should collect samples from more populations in the native ranges of RTB to test our hypothesis on RTB’s movement across North America.

In contrast to the results from native ranges, 5 Chinese populations (Shanxi, Shaanxi, HB, LN, and NM) were clustered into one group (Fig. 4a). The China population was pure in composition of population structure, even representing one of three ancestry populations of RTB according to the population genetic structure analysis (Fig. 4c). However, the CAMT subpopulation was an admixture of three ancestry populations with approximately one third of composition being derived from China population (Fig. 4c). Regarding the lower level of genetic differentiation between the CAMT and China population (Fig. 4d, e), we postulate that China population might originate from CAMT subpopulation, coinciding with the assumption that the RTB that invaded China were most likely from the Pacific Northwest of the USA as reported in Cognato et al. [30]. At the same time, we postulate that the original RTB population that invaded into China from CAMT was pure in the composition of population structure since the China populations are rather pure. The current hybridized pattern of subpopulation CAMT in genetic structure may result from potential gene flow from AZCO and WIMN through natural dispersion and infested log transportation as many international ports occur in the Pacific Northwest.

Strikingly, it was observed that the median genetic diversity of the invasive population of RTB was greater than that of each of three native populations (Fig. 4d), a phenomenon also observed by Cai et al. [31] and apparently in contrast to the genetic paradox of biological invasion reported in most previous studies [79]. High levels of genetic diversity can be maintained, however, if the population expands [80]. We cannot rule out the possibility that the RTB invasive population could harbor higher genetic diversity under certain conditions, such as the occurrence of multiple invasions from different source populations, or the rapid evolutionary adaptation driven by selection during the colonization of new habitats [81, 82]. Recently, it was found that most native populations of the grape phylloxera, *Daktulosphaira vitifoliae* (Fitch), exhibited a higher genetic diversity than did invasive populations, while some introduced populations also showed a higher genetic diversity relative to part of native geographical populations [71]. As the pure population structure pattern was observed in the China population (Fig. 4c), we are more inclined to believe that the Chinese populations of RTB expanded rapidly since introduction.

## Conclusions

A chromosome-level high-quality genome assembly was obtained for the invasive forest pest RTB through integrating multiple sequencing technologies. The genome sequence of RTB provides a new valuable resource in Curculionoidea, many species of which are economically and ecologically important pests in forestry and agriculture. It also provides an important data resource for interaction studies between RTB and its host *P. tabuliformis*, particularly interactions with terpenoids. Moreover, as an invasive species, RTB also serves as a model to decipher the genomic basis of how a secondary pest in its original habitat can become highly destructive in invaded ranges. Additionally, gene family expansion and positive selection analysis revealed the significant enhancement of metabolic capacity, signal transduction, and gene expression regulation in RTB, which might be related to adaptation in new environments. The X-chromosome associated linkage group was determined based on female to male coverage ratio analysis, providing genetic basis for studying sex-specific behaviors. Moreover, genome resequencing analysis highlighted the population genetic structure of RTB in its native and invaded regions, suggesting substantial divergence among American subpopulations. Furthermore, selective sweep analysis showed that multiple genes associated with environmental adaptation and stress resistance underwent strong selection in China. Our work provides an important resource for genomic study in forest bark beetles in general. Moreover, it will also facilitate the functional study and decipher how an invasive beetle adapt to a new host pine *P. tabuliformis* with copious defensive oleoresin in invaded range aided by the recently published genome of *P. tabuliformis* [28].

## Methods

### Sample preparation

Field trapping was conducted using eight-funnel Lindgren traps baited with kairomone attractant (3-carene, 95%, Sigma-Aldrich) in 2016 in a natural stand of *P. tabuliformis* at Beishe Mountain in Shanxi (37° 48′ N, 111° 57′ E, mean elevation 1400 m). Traps were checked daily, and RTB were collected and sexed by stridulation [36]. Ten pairs of RTB were inoculated into holes of pre-drilled pine bolts and reared in a temperature-controlled room (20°C) with natural light to obtain sibling larvae and adults for Hi-C and genome sequencing, respectively.

For population resequencing, RTB field population were collected from multiple states in the native habitat of the USA, and several provinces in the invaded region China by field trapping with eight-funnel Lindgren traps baited with the kairomone attractant and sites were tabulated (Additional file 1: Table S12). In the native range, six states selected in North America, i.e., Arizona (AZ), California (CA), Colorado (CO), Montana (MT), Minnesota (MN), and Wisconsin (WI), represent much of RTB range distribution in North America [75]. In invaded China, five provinces were selected in its main distribution, i.e., Shanxi (SX), Shaanxi (SHX), Hebei (HB), Inner Mongolia (NM), and Liaoning (LN), since Beijing is inside of Hebei province in geographical location. Beetles captured were sexed by male’s stridulation [36]. Beetles were placed in 100% ethanol and kept in −80 °C for later resequencing.

### DNA extraction

Three new emerged males of a single family from a log were pooled to extract DNA for genomic sequencing. Ten beetles of each sex and of each population were individually extracted for population resequencing. Genomic DNA was extracted using the Qiagen DNA purification kit (Qiagen, Valencia, CA, USA). Three new emerged males/single frozen beetle kept in 100% ethanol were/was homogenized using a cell disrupter (BeadBeater; Bio-Spec, Bartlesville, OK, USA) and followed the protocol of the kit to yield genomic DNA. Finally, DNA quantity and quality controls were validated by Qubit, Nanodrop, and Femto Pulse machines.

### Genome survey and sequencing

#### Genome size estimation

To evaluate the genome size of RTB, an Illumina high-throughput sequencing library with an insert size of 350 bp was constructed from genomic DNA and paired-end sequenced on the Illumina HiSeq X Ten platform. Raw reads were preprocessed to obtain clean data using Trimmomatic v0.36 software [83]. Generally speaking, a k-mer analysis was used to evaluate the genome size. In detail, the k-mer frequency analysis was performed using the Jellyfish v2.1.3 software [84] with the default parameters except that *k* was set as 17. Subsequently, the genome size, heterozygosity rate, and repeat content were estimated using GenomeScope 2.0 [85] based on the k-mer frequency distribution.

#### Illumina and PacBio sequencing

For Illumina short-read sequencing, two paired-end sequencing libraries with an insert size of 350 bp were constructed and sequenced on Illumina HiSeq X Ten platform. For Pacific Biosciences (PacBio) long-read sequencing, two SMRT bell sequencing libraries with insert sizes of 40 and 20 kb were constructed and sequenced on the PacBio Sequel sequencing platform, generating 42.3 Gb (40 kb) and 54.16 Gb (20 kb) data separately. The raw sequencing data with adapters, more than 10% of unknown nucleotides (N), and 50% of low quality (Q-value <= 10) bases generated by the Illumina HiSeq X Ten platform were trimmed to obtain high-quality reads. The subreads of PacBio sequencing data were filtered with default parameters.

#### Library construction and 10X Genomics sequencing

The Chromium library was prepared according to 10X Genomics’ protocols using the Genome Reagent Kit v2 (10X Genomics, San Francisco, California, USA). DNA sample quantity and quality controls were validated by Qubit, Nanodrop, and Femto Pulse machines. Briefly, ~10 ng of high molecular weight (HMW) gDNA (mean fragment length > 65 kb) was used for each library. A total of four 10X Genomics linked-read libraries were constructed and sequenced on an Illumina HiSeq X Ten platform.

#### Hi-C library construction and sequencing

One Dovetail Genomics Hi-C library was prepared using the whole body of a single RTB larva which was dealt with 75% alcohol as described previously with certain modifications [86]. Hi-C libraries were controlled for quality and sequenced on an Illumina HiSeq X Ten platform.

### Genome assembly and annotation

#### Genome assembly

The draft genome was assembled using the raw reads generated by the PacBio and Illumina sequencing platform. Regarding the characteristics of reads generated by two platforms, PacBio long reads were employed for the assembly of the genome framework and Illumina short reads were utilized for improving the genome assembly. First, the assembly of the genome framework was conducted using the FALCON assembler v1.2.4 [87] with default parameters. The primary contigs were subsequently polished with PacBio reads using Quiver (SMRT Link v5.0.1). Then, the Purge Haplotigs software [88] was used to remove redundant contigs from the initial assembly, obtaining a non-redundant genome assembly. The resulting contigs were connected to form super-scaffolds by 10X Genomics linked-read data using the fragScaff software (version 140324) [89], and then gap closing was performed using the PBJelly software [90] based on PacBio reads. Finally, the Illumina short reads were used to correct any remaining errors within the genome assembly using the Pilon v1.22 software [91], yielding a final draft genome assembly of RTB.

To generate a chromosome-level genome assembly, Hi-C technology was used to anchor the scaffolds onto chromosomes. First, the high-quality paired-end Hi-C sequencing reads were mapped to RTB draft genome and filtered using HiCUP v0.7.4 [92]. Briefly, the Hi-C reads were truncated at the enzyme digestion ligation site (^GATC) using hicup_truncater that separated two DNA fragments. After truncation, the resulting trimmed forward and reverse reads were mapped to RTB draft genome by Bowtie (v1.3.0) [93] that was embedded in hicup_mapper, respectively. Then, hicup_digester was applied to create digested reference genome. With the information of digested reference genome, sequences representing other uninformative di-tags and experimental Hi-C artifacts were removed, and those unique high-quality alignments were remained to build raw inter or intra-chromosomal contact maps for further analysis. Finally, based on the hierarchical clustering algorithm, the scaffolds were clustered into 14 pseudo-chromosome linkage groups using the ALLHiC pipeline [94].

#### Evaluation of genome assembly

To assess the quality and completeness of the genome assembly, benchmarking universal single-copy orthologs (BUSCO) [95] was performed for evaluation of the assembled genome with the lineage Insecta v9 data set (insecta_odb9, containing 1658 core genes in 42 species) in genome mode.

#### Transposable element annotation

To identify the repetitive elements in RTB, both homology alignment and ab initio prediction methods were used to annotate transposable elements (TEs) in the RTB genome. First, RepeatModeler (http://www.repeatmasker.org/RepeatModeler/), RepeatScout [96], PILER [97], and LTR_FINDER [98] were applied for de novo construction of candidate libraries of repetitive elements of RTB genome. Then, the de novo libraries of repeat sequences in combination with the Repbase database were used to search against RTB genome for the discovery of repeat sequences using RepeatMasker (http://www.repeatmasker.org/). Tandem repeats were also predicted using Tandem Repeat Finder (TRF; v4.07b). Based on the above procedures, repeat sequences of RTB genome were finally annotated.

#### Non-coding RNAs

Canonical small non-coding RNAs, including ribosomal RNAs (rRNAs), transfer RNAs (tRNAs), small nuclear RNAs (snRNAs), and small nucleolar RNAs (snoRNAs), were identified in RTB genome. Firstly, rRNAs were analyzed by searching against the invertebrate rRNA database using BLAST with an E-value of 1e−10. tRNAs were identified using the tRNAscan-SE software [99]. Meanwhile, snRNAs, snoRNAs, and miRNAs were identified using INFERNAL (v1.1rc4) [100] search against the Rfam database.

#### Protein-coding gene annotation

To annotate protein-coding genes in the RTB genome, three types of gene annotation methods, including homology-based annotation, transcriptome-based annotation, and ab initio prediction, were simultaneously employed for obtaining a better gene annotation result. For homology-based annotation, reference protein sequences for nine species downloaded from National Center for Biotechnology Information (NCBI) database were aligned against the RTB genome using TBLASTN v2.2.29+ with an E-value cutoff of 1e−5. All BLAST hits were concatenated by the Solar software (v0.9.6). The genomic region corresponding to the 1000 bp upstream and downstream of each candidate gene was extracted for predicting the exact gene structure using GeneWise v2.4.1 [101]. The resulting homology predictions were denoted as the “Homology set.” For transcriptome-based annotation, RNA-sequencing (RNA-seq) data were assembled using Trinity (v2.1.1) [102]. The assembled sequences were then aligned against the RTB genome using Program to Assemble Spliced Alignment (PASA) [103], by which the effective alignments were clustered based on genome mapping location and assembled into gene structures. The gene models generated by PASA were denoted as the “PASA-T-set (PASA Trinity set).” Further, RNA-seq reads were directly mapped to RTB genome using TopHat v2.0.13 [104]. The mapped reads were assembled into gene models (Cufflinks-set) by Cufflinks v2.1.1 [105]. For ab initio gene prediction, Augustus v3.2.3 [105, 106], GeneID v1.4 [107], GeneScan [108], GlimmerHMM v3.0.4 [109], and SNAP v2013-11-29 [110] were separately employed for gene prediction in the repeat-masked genome (Additional file 1: Table S5). The specific parameters of Augustus, GlimmerHMM, and SNAP were trained with the gene models in PASA-T-set. Finally, all the gene models were integrated into a consensus gene set using EVidenceModeler v1.1.1 [111] with the weights for each type of evidence: PASA-T-set > Homology-set = Cufflinks-set > Augustus > GeneID = SNAP = GlimmerHMM = GeneScan. Furthermore, those genes that encode proteins less than 50 amino acids in length and are supported by only ab initio evidence and with low expression level (< 1.0) were filtered.

#### Functional annotation of protein-coding genes

To consider potential functions of protein-coding genes in RTB, we annotated the official gene set against several commonly used database, including NCBI non-redundant protein database (NR), SwissProt, Pfam, InterPro, and Kyoto Encyclopedia of Genes and Genomes (KEGG). Notably, Pfam domain and gene ontology (GO) information were predicted using the InterProScan tool [112] search against the InterPro database based on conserved protein domains and functional sites. For other database, BLASTP search was performed with an E-value cutoff of 1e−5.

### Comparative genomics

#### OrthoMCL analysis

For comparison analysis between different genomes, the identification of orthologous genes was rather important for subsequent analysis. To identify orthologs and paralogs across different insects, OrthoMCL v2.0.9 software [113] was employed for identification of orthologous groups across insect species. During the orthoMCL analysis, a total of 16 Arthropoda species including 15 insect species and the two-spotted spider mite (*Tetranychus urticae*) were chosen for finding orthologs. For these 15 species (14 insect species and 1 spider mite), the genome assembly and annotation data were downloaded from NCBI RefSeq assembly or Ensembl invertebrate genome database. For genes with multiple alternative isoforms, only the longest transcript was retained for analysis of orthologs. OrthoMCL was performed with the following parameters: match percentage cutoff = 50%, and E-value cutoff = 1e−5. Markov clustering was performed using mcl with an inflation value of 1.5. After OrthoMCL analysis, single-copy orthologs, universal orthologs, and other types of genes were extracted from the clustering result using an in-house Perl script.

#### Phylogenetic analysis of RTB with other insect species

To reveal the phylogenetic relationship between RTB and other insect species, a phylogenetic species tree was reconstructed from RTB and 14 other insects, specifically *Drosophila melanogaster* (BDGP6; from Ensembl), *Ceratitis capitata* (GCF_000347755.3; from NCBI), *Bombyx mori* (ASM15162v1; from Ensembl), *Spodoptera litura* (GCF_002706865.1; from NCBI), *Apis mellifera* (amel_OGSv3.2; from BeeBase), *Nasonia vitripennis* (Nvit_OGSv1.2; from NasoniaBase), *Zootermopsis nevadensis* (GCF_000696155.1; from NCBI), *Anoplophora glabripennis* (Agla_1.0; from Ensembl), *Tribolium castaneum* (Tcas5.2; from Ensembl), *Leptinotarsa decemlineata* (lepdec_OGSv1.2; from i5k), *Hycleus cichorii* (GigaDB, downloaded from ftp://parrot.genomics.cn/gigadb/pub/10.5524/100001_101000/ 100405/), *Dendroctonus ponderosae* (GCF_000355655.1; from NCBI), *Nilaparvata lugens* (GCF_014356525.1; from NCBI), and *Pediculus humanus* (PhumU2.4; from VectorBase). Additionally, the two-spotted spider mite (*Tetranychus urticae*, GCF_000239435.1; from NCBI) was chosen as an outgroup during the inference of the phylogenetic species tree. First, multiple protein sequence alignment was separately performed for each single-copy gene of all these arthropod species using MUSCLE [114] with the default parameters, and the automatic alignment trimming was performed using trimAl 1.2rev59 [115] with the default parameters. Then, the trimmed protein sequences were concatenated into a super-sequence in the same order for each species. Prior to the construction of the phylogenetic tree, the optimal model of protein evolution was selected using ProtTest v3.4.2 [116] with the parameters “-all-distributions -F -AIC -BIC -tc 0.5,” and the best model was selected based on BIC. Subsequently, the maximum-likelihood (ML) phylogenetic species tree was inferred using RAxML v8.2.10 [117] with the best fit-model PROT+GAMMA+ILGF and the rapid bootstrap inference was implemented with 500 duplications.

To infer the divergence times of different species in the phylogeny, MCMCtree program within the Phylogenetic Analysis by Maximum Likelihood (PAML) package [118] was employed for estimation of divergence time with the JC69 model. Additionally, the species divergence time was calibrated with seven calibration time points: 295–305.5 million years ago (Mya) for *D. melanogaster* and *P. humanus*, 175–185 Mya for *A. mellifera* and *N. vitripennis*, 291–359 Mya for *D. melanogaster* and *A. mellifera*, 275–345 Mya for *D. melanogaster* and *T. castaneum*, 79–155 Mya for *D. melanogaster* and *C. capitata*, 162–254 Mya for *A. glabripennis* and *T. castaneum*, and 560–642 Mya for *P. humanus* and *T. urticae*. The calibration time points were collected from the fossil records and the TimeTree website (http://www.timetree.org). The parameters of mcmctree were set as follows: burn-in=5000, sample-number=20000, sample-frequency=50.

#### Gene family expansion and contraction analysis

First, all genes were assigned to corresponding gene families through search against the TreeFam v9 protein database for each species using the tool provided by TreeFam database [119]. Then, the gene family assignment results were merged together to generate a table of gene family member counts (6710 gene families). For the gene families with abnormality in gene count (difference in gene count >10-fold), they were excluded from the downstream analysis using an in-house Perl script, yielding a total of 6457 families for gene expansion and contraction analysis. To determine the change of gene family members of RTB during the evolution, gene gain and loss analysis was conducted using CAFÉ v3.0 [120], in which gene family change was simulated using a stochastic birth and death model. The gene count file and an ultrametric species tree with branch length information were provided as the input for CAFÉ analysis. The optimal lambda parameter was automatically determined by the program. For the CAFÉ analysis result, the gene families with family-wide *P*-value < 0.05 were defined as rapidly evolving families.

#### Positive selection analysis

The protein sequences encoded by single-copy orthologous genes in six Coleoptera species (*D. valens*, *D. ponderosae*, *L. decemlineata*, *H. cichorii*, *A. glabripennis*, and *T. castaneum*) were separately aligned using MUSCLE [114] with the default parameters. Subsequently, the codon multiple sequence alignment was generated based on the protein multiple sequence alignment result using the PAL2NAL software [121]. For each orthologous group, the codeml program as implemented in the PAML package [116] was performed with the branch-site model to determine whether the corresponding gene was positively selected in RTB. Likelihood ratio test (LRT) was performed to determine the statistical significance, and the *P*-value was adjusted using the FDR-based multiple-comparison testing. The genes with FDR < 0.05 were defined as candidate positively selected genes in RTB.

### Genome-wide synteny analysis and identification of sex chromosomes

To reveal the collinearity relationship between RTB and two Coleoptera species (*T. castaneum* and *H. axyridis*), genome-wide synteny analysis was performed using the MCScanX pipeline within JCVI utility libraries [122]. To identify potential sex chromosomes in RTB, genomic DNA from ten adults (five males and five females) was sequenced on an Illumina HiSeq X Ten platform. After filtering adaptor contamination and short or low-quality sequences, clean data were aligned to the reference genome using BWA-MEM v0.7.5a [123] with the default parameters. Read coverage data were compared between female and male samples to detect the sex-associated genomic regions. The read coverage was calculated for each sample using BEDTools [124] with a window size of 1 kb. Then, the average read coverage was computed for each genomic interval in male and female group. Subsequently, the female to male (F: M) coverage ratio was used to determine sex-linked scaffolds. Generally, equal coverage was expected for the autosomes between male and female individuals, while the X chromosome possess approximately twice greater coverage in female than in male and the Y chromosome have approximately twice greater coverage in male than in female. Chromosome with a log_2_(F:M coverage ratio) value approximately 0 was defined as autosomes, and a value approximately 1 as X chromosome, and a value equal to or less than −1 as Y chromosome. An in-house script was employed to identify sex-linked scaffolds based on the F:M coverage ratio.

### Population genomics analysis

#### Genome resequencing

For whole genome resequencing analysis across native and invasive populations, a total of 107 RTB individuals were collected from six states in the USA (10 in Arizona [AZ], 9 in Colorado [CO], 9 in California [CA], 10 in Montana [MT], 10 in Wisconsin [WI], and 10 in Minnesota [MN]) and five provinces and regions in China (10 in Inner Mongolia [NM], 10 in Liaoning [LN], 10 in Hebei [HB], 10 in Shanxi [SX], and 8 in Shaanxi [SHX]). Genomic DNA was extracted from each individual. Subsequently, genomic DNA samples were used for library preparation with TruSeq Nano DNA HT Sample Preparation Kit following the manufacturer’s instructions. The libraries were analyzed for size distribution using Agilent 2100 Bioanalyzer. Finally, these libraries were 150 bp paired-end sequenced on Illumina HiSeq X Ten platform.

#### Single-nucleotide polymorphism (SNP) calling

Raw reads were preprocessed to remove adaptors and low-quality sequences using Trimmomatic v0.36 [83]. For each sample, clean reads were mapped onto the *D. valens* reference genome using Burrows-Wheeler Aligner [123] with the parameters “mem -t 4 -k 32 -M.” Subsequently, Picard toolkit (http://broadinstitute.github.io/picard/) was used to mark and remove PCR duplicates in each sample. To identify SNP sites in resequencing individuals from different locations, the Genome Analysis Toolkit (GATK) v4 [125] was employed for SNP calling with the best-practices pipeline. Raw SNPs were hard filtered using the cutoffs “QD < 2.0 || MQ < 40.0 || FS > 60.0 || SOR > 3.0 || MQRankSum < -12.5 || ReadPosRankSum < -8.0.” For simplicity, only biallelic variation sites were retained for further analysis. The obtained SNPs were filtered using the following criteria: genotype missing rate < 10%, MAF (minor allele frequency) > 0.05, HWE (Hardy-Weinberg Equilibrium) < 0.001. Additionally, insertions and deletions (InDels) were not considered during our downstream analysis.

#### Population genetic structure

To investigate the characteristics of resequencing individuals collected from different geographic locations, population structure was determined using ADMIXTURE v1.0 [126] with the default parameters. To choose the optimal number of ancestral populations (*K*), ADMIXTURE was implemented with *K* ranging from 2 to 7, and the best value for *K* was determined based on the cross-validation error. The format conversion of genomic variants was conducted using plink v1.9 [127] prior to the population structure analysis. To determine the phylogenetic relationship of resequencing individuals, a neighbor-joining (NJ) tree was constructed based on the distance matrix calculated by plink v1.9 using the PHYLIP v3.697 software (https://evolution.genetics.washington.edu/phylip). Furthermore, principal component analysis (PCA) of genome-wide SNP data across all resequencing samples was also conducted to determine the clustering status of subpopulations using SMARTPCA program within the EIGENSOFT software (https://github.com/chrchang/eigensoft). Prior to PCA, SNPs were filtered to retain only genotypes with quality ≥ 30 using a Python script *vcf2smartpca.py* (https://github.com/DeWitP/SFG/blob/master/scripts/vcf2smartpca.py).

#### Genome-wide scan signal of selective sweeps

To identify the genomic regions potentially undergoing strong selective sweeps during the adaption process, fixation index (*F*_ST_) and nucleotide diversity (Pi) were computed for detecting the signatures of selective sweeps based on genome resequencing data of RTB from different geographic populations. To calculate the genetic differentiation index, pairwise *F*_ST_ and Pi were calculated using VCFtools with a slide window of 5 kb and a step size of 2.5 kb [128]. Based on *F*_ST_ values and Pi ratios, genomic regions with *F*_ST_ and Pi ratios that are located at upper 5% quantile were considered as candidate genomic regions undergoing strong selection.

#### Functional enrichment analysis

The relevant functions of the protein-coding genes overlapping with selective sweeps were characterized by searching for over-represented GO (gene ontology) terms and KEGG (Kyoto Encyclopedia of Genes and Genomes) pathways [129]. *Drosophila* protein sequences were used for performing functional enrichment tests on the target genes using the standalone KOBAS 3.0 [130]. The *P*-values were calculated using a hypergeometric distribution test, followed by multiple-comparison testing with false discovery rate (FDR) correction. KEGG pathways with an FDR-corrected *P*-value of <0.05 were considered statistically significantly enriched.

## Supplementary Information

**Additional file 1: Table S1.** Summary statistics of genome sequencing data of *Dendroctonus valens*. **Table S2.** Summary statistics of genome assembly of *Dendroctonus valens*. **Table S3.** BUSCO evaluation result for genome assembly of *Dendroctonus valens*. **Table S4.** Summary statistics of transposable elements in *Dendroctonus valens* genome. **Table S5.** Summary of gene families manually curated in *Dendroctonus valens* genome. **Table S6.** Summary statistics of genome annotation in *Dendroctonus valens* genome. **Table S7.** List of gene families that are unique in *Dendroctonus valens* compared to other three Coleoptera species. **Table S8.** Gene families that are rapidly expanded in *Dendroctonus valens* revealed by CAFE analysis. **Table S9.** Gene families that are rapidly contracted in *Dendroctonus valens* revealed by CAFE analysis. **Table S10.** List of genes that are positively selected in *Dendroctonus valens* revealed by codeml analysis. **Table S11.** Gene ontology enrichment result of positively selected genes in *Dendroctonus valens*. **Table S12.** Sampling site information for genome resequencing of geographical populations. **Table S13.** Summary statistics of genome resequencing data in different populations. **Table S14.** List of genes that undergo selective sweep in the China population compared to CAMT population.

**Additional file 2: Figure S1.** Genome survey result based on k-mer frequency analysis. K-mer frequency analysis was performed using Jellyfish (k-mer = 17) based on Illumina paired-end sequencing reads of genomic DNA. Genome size, repeat sequence content, and heterozygosity ratio were estimated based on k-mer frequency distribution using GenomeScope 2.0. The estimated genome size was 372.97 Mb. **Figure S2.** Linkage group contact map informed by Hi-C sequencing data in the red turpentine beetle genome. Fourteen linkage groups were generated after the clustering of contact map. The color bar indicates the frequency of Hi-C interaction intensity from low (yellow) to high (red) in the plot. **Figure S3.** Venn diagram showing the common and unique gene families across four Coleoptera species. Gene families were assigned by TreeFam database in four Coleoptera species, the red turpentine beetle *Dendroctonus valens*, the mountain pine beetle *Dendroctonus ponderosae*, the red flour beetle *Tribolium castaneum*, and the Asian long-horned beetle *Anoplophora glabripennis*. **Figure S4.** Synteny analysis between *Dendroctonus valens* and two closely related species. Dot plot representation of the syntenic relationship between *D. valens* and the species in the same genus, *Dendroctonus ponderosae*. Notably, *D. valens* linkage groups (LGs) showed strong syntenic relationship with *D. ponderosae* pseudo-chromosomes. Additionally, many fission and fusion events were observed between *D. valens* and *D. ponderosae*. **(b)** Genome-wide synteny relationship between *D. valens* and two Coleoptera insects, *D. ponderosae* and *Tribolium castaneum*. As shown in the figure, Dpochr1 was formed by the fusion of four complete LGs in *D. valens* (i.e. LG1, LG4, LG10, and LG11). By contrast, Dpochr9 fused with Dpochr12 to generate LG13 of *D. valens*. Genome-wide synteny analysis was performed using the MCScan pipeline of JCVI utility libraries. **Figure S5.** Global representation of sampling sites of different geographical populations and RTB movement. The putative invasion and spread routes was also indicated in the map based on data collected from literature (The green dot denotes the area Canada where RTB originates. The orange solid arrows indicate the west route of RTB spread in North America, and the blue solid arrows show the east route of RTB spread in North America. The red dotted arrow represents the putative invasion route from the west coast of North America to Shanxi province of China). Resequencing samples were collected from six states (red dots) in the original country North America (including Arizona [AZ], Colorado [CO], California [CA], Montana [MT], Wisconsin [WI], and Minnesota [MN]) and five provinces (blue dots ) in the invaded country China (including Liaoning [LN], Inner Mongolia [NM], Hebei [HB], Shanxi [SX], and Shaanxi [SHX]). **Figure S6.** Number of genomic regions showing signals of selective sweep. Selective sweep analysis was conducted based on genetic differentiation index (*F*_ST_) and nucleotide diversity (Pi) ratio in three contrast groups, including AZCO vs. CHN **(a)**, CAMT vs. CHN **(b)**, and WIMN vs. CHN **(c)**. The left panel represents the selection status in North American subpopulations, and the right panel stands for the selection status in China populations. The genomic regions that were under selection were determined by the intersection set of *F*_ST_ outliers (top 5% quantile) and Pi ratio outliers (top 5% quantile), which corresponds to the overlapping part in the Venn diagram.

## Data Availability

The whole genome shotgun project of RTB has been deposited at the public NCBI under BioProject PRJNA765904 (https://www.ncbi.nlm.nih.gov/bioproject/PRJNA765904) [131]. The raw sequencing data used for genome assembly and the raw sequencing data used for resequencing analysis are available at SRA site with accession number for each library. The genome assembly data have been deposited at GenBank under accession no. JAJTJO000000000 (https://www.ncbi.nlm.nih.gov/assembly/GCA_024550625.1/#/def) [132]. Additionally, the genome assembly and annotation data have been deposited in the Figshare database (10.6084/m9.figshare.19999844) [133].

## Abbreviations

ABC: ATP-binding cassette
AZ: Arizona
AZCO: Arizona-Colorado
BUSCO: Benchmarking universal single-copy orthologs
CA: California
CAMT: California-Montana
CHN: China
CO: Colorado
GATK: Genome Analysis Toolkit
GO: Gene Ontology
HB: Hebei
HMW: High molecular weight
HSPs: Heat shock proteins
InDels: Insertions and deletions
KEGG: Kyoto Encyclopedia of Genes and Genomes
LG: Linkage group
LINE: Long interspersed nuclear elements
LN: Liaoning
LTR-RT: Long terminal repeat retrotransposons
Mya: Million years ago
MN: Minnesota
MPB: Mountain pine beetle
MT: Montana
NCBI: National Center for Biotechnology Information
NM: Inner Mongolia
NR: Non-redundant protein database
OGS: Official gene set
PacBio: Pacific Biosciences
PAML: Phylogenetic Analysis by Maximum Likelihood
PCA: Principal component analysis
Pfam: Protein family
PNW: Pacific Northwest of North America
PSGs: Positively selected genes
rRNAs: Ribosomal RNAs
RTB: Red turpentine beetle
SHX: Shaanxi
SINE: Short interspersed nuclear elements
SMRT: Single molecule real-time
snRNAs: Small nuclear RNAs
snoRNAs: Small nucleolar RNAs
SNP: Single-nucleotide polymorphism
SX: Shanxi
TE: Transposable element
tRNAs: Transfer RNAs
WI: Wisconsin
WIMN: Wisconsin-Minnesota
