# Supplementary material for: Chromosome-level genome assembly and population genomic analyses provide insights into adaptive evolution of the red turpentine beetle, Dendroctonus valens
Source: BMC Biol. 2022 Aug 24;20:190. doi: 10.1186/s12915-022-01388-y (PMC9400205; doi:10.1186/s12915-022-01388-y)
Supplement: Supplementary file 1 — Additional file 1: Table S1. Summary statistics of genome sequencing data of Dendroctonus valens. Table S2. Summary statistics of genome assembly of Dendroctonus valens. Table S3. BUSCO evaluation result for genome assembly of Dendroctonus valens. Table S4. Summary statistics of transposable elements in Dendroctonus valens genome. Table S5. Summary of gene families manually curated in Dendroctonus valens genome. Table S6. Summary statistics of genome annotation in Dendroctonus valens genome. Table S7. List of gene families that are unique in Dendroctonus valens compared to other three Coleoptera species. Table S8. Gene families that are rapidly expanded in Dendroctonus valens revealed by CAFE analysis. Table S9. Gene families that are rapidly contracted in Dendroctonus valens revealed by CAFE analysis. Table S10. List of genes that are positively selected in Dendroctonus valens revealed by codeml analysis. Table S11. Gene ontology enrichment result of positively selected genes in Dendroctonus valens. Table S12. Sampling site information for genome resequencing of geographical populations. Table S13. Summary statistics of genome resequencing data in different populations. Table S14. List of genes that undergo selective sweep in the China population compared to CAMT population. [file 12915_2022_1388_MOESM1_ESM.zip › Table S10.docx]

| **Table** **S10** List of genes that are positively selected in *Dendroctonus valens* revealed by codeml analysis | | | | | |
| --- | --- | --- | --- | --- | --- |
| **Gene ID** | **Identity** | **Aligned length** | **Score** | **E-value** | **Description** |
| evm.model.scaffold_51.147 | 0.43 | 206 | 140 | 3.00E-41 | Akirin OS=Drosophila melanogaster GN=akirin PE=1 SV=1 |
| evm.model.scaffold_554.4.2 | 0.3 | 842 | 312 | 9.00E-85 | Ankyrin-1 OS=Homo sapiens GN=ANK1 PE=1 SV=3 |
| evm.model.scaffold_53.93 | 0.6 | 866 | 997 | 0 | Protein retinal degeneration B OS=Drosophila melanogaster GN=rdgB PE=1 SV=2 |
| evm.model.scaffold_94.189 | 0.4 | 749 | 493 | 1.00E-150 | Tubby-related protein 4 OS=Mus musculus GN=Tulp4 PE=2 SV=1 |
| evm.model.scaffold_162.8 | 0.46 | 314 | 324 | 1.00E-94 | RanBP-type and C3HC4-type zinc finger-containing protein 1 OS=Dicentrarchus labrax GN=rbck1 PE=3 SV=1 |
| evm.model.scaffold_10.110 | 0.38 | 330 | 163 | 4.00E-44 | GPI transamidase component PIG-S OS=Rattus norvegicus GN=Pigs PE=2 SV=3 |
| evm.model.scaffold_53.36 | 0.45 | 515 | 425 | 1.00E-130 | DNA repair protein REV1 OS=Gallus gallus GN=REV1 PE=2 SV=1 |
| evm.model.scaffold_102.100 | 0.36 | 883 | 562 | 0 | Putative ATP-dependent RNA helicase DDX11-like protein 8 OS=Homo sapiens GN=DDX11L8 PE=1 SV=1 |
| evm.model.scaffold_16.1 | 0.53 | 2379 | 2340 | 0 | CCR4-NOT transcription complex subunit 1 OS=Mus musculus GN=Cnot1 PE=1 SV=2 |
| evm.model.scaffold_116.22 | 0.55 | 70 | 79.7 | 5.00E-15 | Polymerase delta-interacting protein 3 OS=Homo sapiens GN=POLDIP3 PE=1 SV=2 |
| evm.model.scaffold_447.36 | 0.72 | 151 | 230 | 9.00E-65 | Protein dead ringer OS=Drosophila melanogaster GN=retn PE=1 SV=2 |
| evm.model.scaffold_42.72 | 0.43 | 362 | 198 | 2.00E-50 | Protein lingerer OS=Aedes aegypti GN=lig PE=3 SV=1 |
| evm.model.scaffold_102.23 | 0.61 | 165 | 202 | 1.00E-58 | U11/U12 small nuclear ribonucleoprotein 35 kDa protein OS=Xenopus laevis GN=snrnp35 PE=2 SV=1 |
| evm.model.scaffold_71.77 | 0.52 | 88 | 93.6 | 4.00E-18 | Mediator of RNA polymerase II transcription subunit 26 OS=Drosophila melanogaster GN=MED26 PE=1 SV=2 |
| evm.model.scaffold_296.33 | 0.45 | 207 | 190 | 1.00E-56 | Glutathione S-transferase 1, isoform D OS=Anopheles gambiae GN=GstD1 PE=1 SV=1 |
| evm.model.scaffold_60.10 | 0.36 | 793 | 549 | 0 | Transducin beta-like protein 3 OS=Homo sapiens GN=TBL3 PE=1 SV=2 |
| evm.model.scaffold_24.23 | 0.41 | 413 | 269 | 1.00E-79 | PREDICTED: zinc finger protein 396 [Tribolium castaneum] |
| evm.model.scaffold_359.16 | 0.38 | 282 | 197 | 1.00E-57 | Zinc finger MYND domain-containing protein 10 homolog OS=Drosophila melanogaster GN=CG11253 PE=1 SV=1 |
| evm.model.scaffold_24.196 | 0.47 | 352 | 333 | 1.00E-111 | Cysteine protease ATG4B OS=Xenopus laevis GN=atg4b PE=2 SV=1 |
| evm.model.scaffold_82.23 | 0.46 | 115 | 131 | 5.00E-32 | Zinc finger CCCH domain-containing protein 7 OS=Arabidopsis thaliana GN=At1g21570 PE=1 SV=1 |
| evm.model.scaffold_37.142.1 | 0.76 | 624 | 967 | 0 | PREDICTED: chitin deacetylase 5 isoform X5 [Tribolium castaneum] |
| evm.model.scaffold_463.277 | 0.63 | 463 | 589 | 0 | Nucleolar protein 58 OS=Rattus norvegicus GN=Nop58 PE=1 SV=1 |
| evm.model.scaffold_24.235.4 | 0.59 | 77 | 105 | 3.00E-27 | TATA box-binding protein-like protein 1 OS=Xenopus tropicalis GN=tbpl1 PE=2 SV=1 |
| evm.model.scaffold_588.11 | 0.33 | 2565 | 1176 | 0 | Leucine-rich repeat serine/threonine-protein kinase 1 OS=Caenorhabditis elegans GN=lrk-1 PE=1 SV=6 |
| evm.model.scaffold_882.28 | 0.71 | 90 | 155 | 5.00E-42 | Golgi reassembly-stacking protein 2 OS=Rattus norvegicus GN=Gorasp2 PE=1 SV=3 |
| evm.model.scaffold_509.51 | 0.59 | 247 | 317 | 1.00E-107 | Endonuclease III-like protein 1 OS=Gallus gallus GN=NTHL1 PE=2 SV=1 |
| evm.model.scaffold_108.31.1 | 0.47 | 161 | 153 | 2.00E-46 | Protein CutA homolog OS=Xenopus laevis GN=cuta PE=2 SV=2 |
| evm.model.scaffold_463.272 | 0.45 | 845 | 725 | 0 | Probable leucine--tRNA ligase, mitochondrial OS=Mus musculus GN=Lars2 PE=1 SV=1 |
| evm.model.scaffold_776.26 | 0.4 | 668 | 508 | 1.00E-166 | Phospholipase DDHD2 OS=Mus musculus GN=Ddhd2 PE=2 SV=3 |
| evm.model.scaffold_51.63 | 0.56 | 198 | 195 | 2.00E-54 | Protein abrupt OS=Drosophila melanogaster GN=ab PE=1 SV=2 |
| evm.model.scaffold_195.22 | 0.59 | 214 | 274 | 7.00E-78 | Trimethylguanosine synthase OS=Rattus norvegicus GN=Tgs1 PE=1 SV=1 |
| evm.model.scaffold_37.27 | 0.59 | 380 | 431 | 1.00E-149 | Bifunctional arginine demethylase and lysyl-hydroxylase PSR OS=Drosophila melanogaster GN=PSR PE=2 SV=1 |
| evm.model.scaffold_766.51 | 0.62 | 380 | 493 | 1.00E-172 | Serine/threonine-protein phosphatase 2A regulatory subunit B'' subunit gamma OS=Xenopus tropicalis GN=ppp2r3c PE=2 SV=1 |
| evm.model.scaffold_867.86 | 0.66 | 1203 | 1541 | 0 | Regulator of nonsense transcripts 1 homolog OS=Drosophila melanogaster GN=Upf1 PE=1 SV=2 |
| evm.model.scaffold_51.54 | 0.38 | 345 | 233 | 2.00E-70 | Cell division control protein 6 homolog OS=Homo sapiens GN=CDC6 PE=1 SV=1 |
| evm.model.scaffold_608.89 | 0.26 | 656 | 154 | 2.00E-36 | Hemicentin-1 OS=Homo sapiens GN=HMCN1 PE=1 SV=2 |
| evm.model.scaffold_447.20 | 0.41 | 618 | 471 | 1.00E-158 | Tubulin--tyrosine ligase-like protein 12 OS=Mus musculus GN=Ttll12 PE=2 SV=1 |
| evm.model.scaffold_882.18 | 0.42 | 713 | 532 | 1.00E-179 | Transmembrane and TPR repeat-containing protein 4 OS=Mus musculus GN=Tmtc4 PE=2 SV=1 |
| evm.model.scaffold_51.159 | 0.33 | 359 | 211 | 2.00E-62 | DNA polymerase epsilon subunit 2 OS=Gallus gallus GN=POLE2 PE=2 SV=1 |
| evm.model.scaffold_85.165 | 0.25 | 573 | 201 | 3.00E-52 | Transient receptor potential cation channel subfamily V member 6 OS=Homo sapiens GN=TRPV6 PE=1 SV=3 |
| evm.model.scaffold_81.77 | 0.62 | 1559 | 1824 | 0 | DNA topoisomerase 2 OS=Bombyx mori GN=TOP2 PE=2 SV=1 |
| evm.model.scaffold_116.28 | 0.46 | 1560 | 1417 | 0 | Glycogen debranching enzyme OS=Homo sapiens GN=AGL PE=1 SV=3 |
| evm.model.scaffold_94.116 | 0.64 | 954 | 1229 | 0 | Lon protease homolog, mitochondrial OS=Drosophila melanogaster GN=Lon PE=1 SV=1 |
| evm.model.scaffold_85.256 | 0.3 | 528 | 228 | 2.00E-66 | Protein spartin OS=Drosophila melanogaster GN=spartin PE=1 SV=1 |
| evm.model.scaffold_67.32 | 0.29 | 2826 | 1187 | 0 | Small subunit processome component 20 homolog OS=Homo sapiens GN=UTP20 PE=1 SV=3 |
| evm.model.scaffold_104.43 | 0.92 | 25 | 55.1 | 4.00E-06 | hypothetical protein TcasGA2_TC000560 [Tribolium castaneum] |
| evm.model.scaffold_739.14 | 0.36 | 268 | 174 | 2.00E-43 | Transport and Golgi organization protein 6 homolog OS=Homo sapiens GN=TANGO6 PE=1 SV=2 |
| evm.model.scaffold_94.50 | 0.82 | 347 | 601 | 0 | Fizzy-related protein homolog OS=Mus musculus GN=Fzr1 PE=1 SV=1 |
| evm.model.scaffold_867.51 | 0.44 | 397 | 350 | 1.00E-114 | Chromatin assembly factor 1 subunit B OS=Mus musculus GN=Chaf1b PE=2 SV=1 |
| evm.model.scaffold_323.11 | 0.36 | 504 | 211 | 6.00E-62 | Protein LTV1 homolog OS=Drosophila melanogaster GN=CG7686 PE=1 SV=1 |
| evm.model.scaffold_55.140 | 0.78 | 1017 | 1657 | 0 | UDP-N-acetylglucosamine--peptide N-acetylglucosaminyltransferase 110 kDa subunit OS=Oryctolagus cuniculus GN=OGT PE=1 SV=2 |
| evm.model.scaffold_322.30 | 0.43 | 312 | 245 | 1.00E-65 | SUN domain-containing ossification factor OS=Homo sapiens GN=SUCO PE=1 SV=1 |
| evm.model.scaffold_85.84 | 0.51 | 135 | 125 | 4.00E-30 | Microtubule-associated protein 2 OS=Rattus norvegicus GN=Map2 PE=1 SV=3 |
| evm.model.scaffold_117.39 | 0.51 | 244 | 241 | 3.00E-79 | ATP synthase subunit b, mitochondrial OS=Drosophila melanogaster GN=ATPsyn-b PE=2 SV=2 |
| evm.model.scaffold_79.13 | 0.3 | 575 | 206 | 5.00E-57 | Dolichyl-diphosphooligosaccharide--protein glycosyltransferase subunit 2 OS=Pongo abelii GN=RPN2 PE=2 SV=1 |
| evm.model.scaffold_55.23 | 0.41 | 239 | 171 | 2.00E-49 | Myeloid leukemia factor OS=Drosophila melanogaster GN=Mlf PE=1 SV=2 |
| evm.model.scaffold_117.35 | 0.33 | 216 | 107 | 8.00E-27 | Origin recognition complex subunit 6 OS=Drosophila melanogaster GN=Orc6 PE=1 SV=2 |
| evm.model.scaffold_502.57 | 0.51 | 729 | 634 | 0 | Nuclear hormone receptor FTZ-F1 beta OS=Drosophila melanogaster GN=Hr39 PE=1 SV=3 |
| evm.model.scaffold_57.81 | 0.32 | 508 | 245 | 4.00E-70 | Serine/threonine-protein phosphatase 2A 65 kDa regulatory subunit A beta isoform OS=Rattus norvegicus GN=Ppp2r1b PE=2 SV=1 |
| evm.model.scaffold_647.100 | 0.45 | 508 | 442 | 1.00E-140 | PHD finger protein 14 OS=Mus musculus GN=Phf14 PE=1 SV=1 |
| evm.model.scaffold_94.39.1 | 0.35 | 366 | 212 | 7.00E-55 | E3 ubiquitin-protein ligase SHPRH OS=Homo sapiens GN=SHPRH PE=1 SV=2 |
| evm.model.scaffold_739.37 | 0.38 | 743 | 446 | 1.00E-137 | Ubiquitin carboxyl-terminal hydrolase 64E OS=Drosophila melanogaster GN=Ubp64E PE=1 SV=2 |
| evm.model.scaffold_57.19 | 0.53 | 295 | 315 | 1.00E-105 | Magnesium transporter NIPA2 OS=Bos taurus GN=NIPA2 PE=2 SV=1 |
| evm.model.scaffold_515.13 | 0.7646 | 480 | 719 | 0 | hypothetical protein YQE_10496, partial [Dendroctonus ponderosae] |
| evm.model.scaffold_14.49 | 0.48 | 132 | 135 | 2.00E-33 | TRAF3-interacting protein 1 OS=Rattus norvegicus GN=Traf3ip1 PE=2 SV=1 |
| evm.model.scaffold_867.16 | 0.29 | 510 | 207 | 7.00E-60 | T-cell activation inhibitor, mitochondrial OS=Mus musculus GN=TCAIM PE=2 SV=1 |
| evm.model.scaffold_20.46 | 0.78 | 529 | 866 | 0 | T-complex protein 1 subunit eta OS=Gallus gallus GN=CCT7 PE=1 SV=1 |
| evm.model.scaffold_324.21 | 0.4 | 395 | 300 | 8.00E-93 | F-box only protein 42 OS=Mus musculus GN=Fbxo42 PE=2 SV=1 |
| evm.model.scaffold_31.253 | 0.28 | 204 | 65.1 | 2.00E-09 | Heparanase OS=Homo sapiens GN=HPSE PE=1 SV=2 |
| evm.model.scaffold_106.36 | 0.32 | 164 | 80.1 | 2.00E-16 | 45 kDa calcium-binding protein OS=Danio rerio GN=sdf4 PE=2 SV=1 |
| evm.model.scaffold_749.46.1 | 0.38 | 474 | 278 | 9.00E-87 | Slowpoke-binding protein OS=Drosophila melanogaster GN=Slob PE=1 SV=2 |
| evm.model.scaffold_194.23 | 0.53 | 421 | 407 | 1.00E-137 | Carboxypeptidase E OS=Rattus norvegicus GN=Cpe PE=1 SV=1 |
| evm.model.scaffold_599.13 | 0.72 | 282 | 420 | 2.00E-144 | hypothetical protein YQE_11806, partial [Dendroctonus ponderosae] |
| evm.model.scaffold_21.12 | 0.3 | 557 | 187 | 1.00E-51 | La-related protein 7 OS=Danio rerio GN=larp7 PE=2 SV=1 |
| evm.model.scaffold_844.7 | 0.55 | 271 | 298 | 9.00E-97 | E3 ubiquitin-protein ligase RNF144A OS=Homo sapiens GN=RNF144A PE=1 SV=2 |
| evm.model.scaffold_864.54 | 0.52 | 544 | 535 | 0 | Potassium voltage-gated channel subfamily KQT member 1 OS=Squalus acanthias GN=KCNQ1 PE=2 SV=1 |
| evm.model.scaffold_322.23 | 0.44 | 562 | 442 | 1.00E-137 | Period circadian protein OS=Periplaneta americana GN=per PE=2 SV=2 |
| evm.model.scaffold_463.163 | 0.32 | 577 | 296 | 8.00E-80 | Cat eye syndrome critical region protein 2 OS=Homo sapiens GN=CECR2 PE=1 SV=2 |
| evm.model.scaffold_24.45 | 0.52 | 278 | 275 | 3.00E-86 | Zinc finger protein ZIC 4 OS=Xenopus laevis GN=zic4 PE=2 SV=1 |
| evm.model.scaffold_109.39 | 0.4 | 321 | 214 | 8.00E-65 | rRNA methyltransferase 3, mitochondrial OS=Homo sapiens GN=RNMTL1 PE=1 SV=2 |
| evm.model.scaffold_17.29 | 0.58 | 373 | 436 | 1.00E-145 | Fidgetin-like protein 1 OS=Rattus norvegicus GN=Fignl1 PE=2 SV=1 |
| evm.model.scaffold_62.136 | 0.67 | 82 | 108 | 5.00E-29 | Protein FAM192A OS=Mus musculus GN=Fam192a PE=2 SV=1 |
| evm.model.scaffold_554.27 | 0.64 | 314 | 426 | 1.00E-140 | Glycine dehydrogenase (decarboxylating), mitochondrial OS=Homo sapiens GN=GLDC PE=1 SV=2 |
| evm.model.scaffold_99.85 | 0.36 | 631 | 352 | 1.00E-112 | Probable nucleoporin Nup54 OS=Drosophila melanogaster GN=Nup54 PE=2 SV=1 |
| evm.model.scaffold_31.276 | 0.37 | 174 | 110 | 1.00E-24 | Protein aurora borealis OS=Drosophila melanogaster GN=bora PE=1 SV=1 |
| evm.model.scaffold_633.30 | 0.31 | 420 | 202 | 3.00E-54 | General transcription factor 3C polypeptide 3 OS=Homo sapiens GN=GTF3C3 PE=1 SV=1 |
| evm.model.scaffold_82.7 | 0.28 | 337 | 125 | 8.00E-28 | Protein piccolo (Fragment) OS=Gallus gallus GN=PCLO PE=2 SV=1 |
| evm.model.scaffold_32.10 | 0.46 | 1763 | 1434 | 0 | WD repeat and FYVE domain-containing protein 3 OS=Homo sapiens GN=WDFY3 PE=1 SV=2 |
| evm.model.scaffold_90.43 | 0.45 | 822 | 660 | 0 | ATP-dependent RNA helicase DDX54 OS=Mus musculus GN=Ddx54 PE=1 SV=1 |
| evm.model.scaffold_535.5 | 0.53 | 223 | 202 | 4.00E-63 | Retinol dehydrogenase 13 OS=Homo sapiens GN=RDH13 PE=1 SV=2 |
| evm.model.scaffold_94.101 | 0.51 | 1343 | 1305 | 0 | Neuropathy target esterase sws OS=Drosophila virilis GN=sws PE=3 SV=1 |
| evm.model.scaffold_615.24 | 0.27 | 487 | 124 | 1.00E-28 | RNA polymerase II-associated protein 3 OS=Gallus gallus GN=RPAP3 PE=2 SV=1 |
| evm.model.scaffold_37.325 | 0.46 | 108 | 97.1 | 2.00E-19 | SAFB-like transcription modulator OS=Mus musculus GN=Sltm PE=1 SV=1 |
| evm.model.scaffold_653.16 | 0.36 | 797 | 497 | 1.00E-160 | Ubiquitin conjugation factor E4 A OS=Bos taurus GN=UBE4A PE=2 SV=1 |
| evm.model.scaffold_818.53.1 | 0.42 | 610 | 499 | 1.00E-160 | Lethal(3)malignant brain tumor-like protein 3 OS=Homo sapiens GN=L3MBTL3 PE=1 SV=2 |
| evm.model.scaffold_81.82 | 0.39 | 352 | 265 | 1.00E-77 | High affinity cAMP-specific 3',5'-cyclic phosphodiesterase 7A OS=Homo sapiens GN=PDE7A PE=1 SV=2 |
| evm.model.scaffold_882.25 | 0.34 | 1178 | 603 | 0 | Fibronectin type-III domain-containing protein 3a OS=Gallus gallus GN=FNDC3A PE=2 SV=2 |
| evm.model.scaffold_329.30 | 0.33 | 182 | 100 | 1.00E-23 | Transmembrane protein 192 OS=Danio rerio GN=tmem192 PE=2 SV=1 |
| evm.model.scaffold_85.94 | 0.41 | 388 | 221 | 4.00E-61 | Double-strand-break repair protein rad21 homolog OS=Mus musculus GN=Rad21 PE=1 SV=3 |
| evm.model.scaffold_119.39 | 0.75 | 286 | 453 | 1.00E-152 | Cytoplasmic tRNA 2-thiolation protein 1 OS=Drosophila melanogaster GN=CG8078 PE=1 SV=1 |
| evm.model.scaffold_181.18 | 0.34 | 294 | 175 | 4.00E-51 | Gamma-soluble NSF attachment protein OS=Mus musculus GN=Napg PE=1 SV=1 |
| evm.model.scaffold_329.46 | 0.34 | 195 | 114 | 9.00E-25 | Disks large-associated protein 1 OS=Homo sapiens GN=DLGAP1 PE=1 SV=1 |
| evm.model.scaffold_53.120 | 0.4 | 339 | 239 | 2.00E-66 | N-acetylglucosamine-1-phosphotransferase subunits alpha/beta OS=Danio rerio GN=gnptab PE=1 SV=1 |
| evm.model.scaffold_544.28 | 0.61 | 99 | 120 | 1.00E-32 | Peptidyl-prolyl cis-trans isomerase NIMA-interacting 4 OS=Danio rerio GN=pin4 PE=2 SV=1 |
| evm.model.scaffold_882.3 | 0.51 | 1700 | 1652 | 0 | E3 ubiquitin-protein ligase TRIP12 OS=Mus musculus GN=Trip12 PE=1 SV=1 |
| evm.model.scaffold_393.5 | 0.78 | 324 | 523 | 1.00E-171 | Roquin-1 OS=Xenopus laevis GN=rc3h1 PE=2 SV=1 |
| evm.model.scaffold_766.30 | 0.33 | 670 | 295 | 2.00E-86 | IQ and AAA domain-containing protein 1-like OS=Mus musculus GN=Iqca1l PE=2 SV=3 |
| evm.model.scaffold_181.23.1 | 0.31 | 218 | 115 | 2.00E-25 | Chromodomain Y-like protein 2 OS=Homo sapiens GN=CDYL2 PE=1 SV=2 |
| evm.model.scaffold_771.9 | 0.3 | 1057 | 493 | 1.00E-156 | Structural maintenance of chromosomes protein 5 OS=Takifugu rubripes GN=smc5 PE=2 SV=1 |
| evm.model.scaffold_51.127 | 0.85 | 289 | 491 | 1.00E-169 | hypothetical protein YQE_04886, partial [Dendroctonus ponderosae] |
| evm.model.scaffold_771.42 | 0.28 | 356 | 135 | 5.00E-31 | Glutamine-rich protein 2 OS=Homo sapiens GN=QRICH2 PE=1 SV=1 |
| evm.model.scaffold_24.89 | 0.62 | 64 | 89.4 | 5.00E-23 | NADH dehydrogenase [ubiquinone] iron-sulfur protein 6, mitochondrial OS=Macaca fascicularis GN=NDUFS6 PE=2 SV=1 |
| evm.model.scaffold_524.32 | 0.65 | 126 | 189 | 3.00E-60 | Alpha-ketoglutarate-dependent dioxygenase alkB homolog 6 OS=Homo sapiens GN=ALKBH6 PE=1 SV=2 |
| evm.model.scaffold_365.1 | 0.83 | 535 | 873 | 0 | Potassium voltage-gated channel protein Shaker OS=Drosophila melanogaster GN=Sh PE=1 SV=3 |
| evm.model.scaffold_728.10 | 0.46 | 541 | 493 | 1.00E-169 | Nedd8-activating enzyme E1 regulatory subunit OS=Drosophila melanogaster GN=APP-BP1 PE=1 SV=1 |
| evm.model.scaffold_633.27 | 0.45 | 72 | 73.2 | 7.00E-14 | tRNA-splicing endonuclease subunit Sen2 OS=Rattus norvegicus GN=Tsen2 PE=2 SV=1 |
| evm.model.scaffold_24.148 | 0.43 | 869 | 661 | 0 | Solute carrier family 12 member 9 OS=Xenopus laevis GN=slc12a9 PE=2 SV=1 |
| evm.model.scaffold_64.4 | 0.32 | 928 | 381 | 1.00E-118 | Protein smg8 OS=Xenopus tropicalis GN=smg8 PE=2 SV=1 |
| evm.model.scaffold_842.121 | 0.4 | 292 | 201 | 1.00E-61 | UPF0160 protein MYG1, mitochondrial OS=Bos taurus PE=2 SV=1 |
| evm.model.scaffold_608.77.1 | 0.58 | 417 | 477 | 1.00E-161 | Fragile X mental retardation syndrome-related protein 1 OS=Drosophila melanogaster GN=Fmr1 PE=1 SV=1 |
| evm.model.scaffold_451.21 | 0.56 | 371 | 426 | 1.00E-145 | Ankyrin repeat domain-containing protein 13C OS=Homo sapiens GN=ANKRD13C PE=2 SV=2 |
| evm.model.scaffold_647.50 | 0.7 | 587 | 897 | 0 | Eukaryotic translation initiation factor 5B OS=Homo sapiens GN=EIF5B PE=1 SV=4 |
| evm.model.scaffold_37.195 | 0.29 | 524 | 206 | 5.00E-54 | Atrial natriuretic peptide-converting enzyme OS=Homo sapiens GN=CORIN PE=1 SV=2 |
| evm.model.scaffold_53.175 | 0.29 | 332 | 117 | 2.00E-26 | Putative protein tag-52 OS=Caenorhabditis elegans GN=tag-52 PE=3 SV=1 |
| evm.model.scaffold_647.71 | 0.49 | 143 | 138 | 2.00E-39 | Membrane-associated progesterone receptor component 2 OS=Homo sapiens GN=PGRMC2 PE=1 SV=1 |
| evm.model.scaffold_890.198 | 0.29 | 289 | 103 | 2.00E-23 | G patch domain and KOW motifs-containing protein OS=Mus musculus GN=Gpkow PE=2 SV=2 |
| evm.model.scaffold_916.61 | 0.51 | 338 | 300 | 3.00E-95 | ATPase family AAA domain-containing protein 3 OS=Xenopus tropicalis GN=atad3 PE=2 SV=1 |
| evm.model.scaffold_37.368 | 0.52 | 538 | 553 | 0 | GTP-binding protein 2 OS=Homo sapiens GN=GTPBP2 PE=1 SV=1 |
| evm.model.scaffold_463.260 | 0.37 | 243 | 172 | 7.00E-52 | Mitochondrial inner membrane protease ATP23 homolog OS=Danio rerio GN=zgc:162885 PE=2 SV=1 |
| evm.model.scaffold_87.13.1 | 0.59 | 247 | 288 | 8.00E-98 | NECAP-like protein CG9132 OS=Drosophila melanogaster GN=CG9132 PE=2 SV=1 |
| evm.model.scaffold_53.46 | 0.62 | 1058 | 1388 | 0 | Ubiquitin-like modifier-activating enzyme 1 OS=Bos taurus GN=UBA1 PE=2 SV=1 |
| evm.model.scaffold_529.21 | 0.56 | 116 | 141 | 2.00E-41 | PRKR-interacting protein 1 homolog OS=Xenopus laevis GN=prkrip1 PE=2 SV=1 |
| evm.model.scaffold_61.28 | 0.39 | 68 | 51.6 | 2.00E-07 | Probable peroxisomal membrane protein PEX13 OS=Caenorhabditis elegans GN=prx-13 PE=3 SV=2 |
| evm.model.scaffold_24.40 | 0.49 | 596 | 585 | 0 | tRNA-dihydrouridine(47) synthase [NAD(P)(+)]-like OS=Xenopus laevis GN=dus3l PE=2 SV=1 |
| evm.model.scaffold_85.122.1 | 0.59 | 253 | 332 | 1.00E-102 | Actin-binding LIM protein 1 OS=Homo sapiens GN=ABLIM1 PE=1 SV=3 |
| evm.model.scaffold_51.34 | 0.37 | 195 | 124 | 1.00E-31 | UMP-CMP kinase 2, mitochondrial OS=Homo sapiens GN=CMPK2 PE=1 SV=3 |
| evm.model.scaffold_822.16 | 0.56 | 225 | 277 | 1.00E-83 | Phosphorylated CTD-interacting factor 1 OS=Homo sapiens GN=PCIF1 PE=1 SV=1 |
| evm.model.scaffold_513.55 | 0.24 | 403 | 124 | 7.00E-28 | Putative methyltransferase NSUN7 OS=Mus musculus GN=Nsun7 PE=2 SV=2 |
| evm.model.scaffold_463.128 | 0.41 | 295 | 197 | 2.00E-60 | Methyltransferase-like protein 9 OS=Gallus gallus GN=METTL9 PE=2 SV=1 |
| evm.model.scaffold_818.24 | 0.33 | 1168 | 591 | 1.00E-176 | Protein abnormal spindle OS=Drosophila melanogaster GN=asp PE=1 SV=3 |
| evm.model.scaffold_37.234 | 0.55 | 106 | 134 | 3.00E-34 | SH2 domain-containing adapter protein F OS=Mus musculus GN=Shf PE=1 SV=2 |
| evm.model.scaffold_79.6 | 0.55 | 331 | 403 | 1.00E-140 | Geranylgeranyl transferase type-2 subunit beta OS=Bos taurus GN=RABGGTB PE=2 SV=1 |
| evm.model.scaffold_51.149 | 0.56 | 283 | 333 | 1.00E-112 | Kidney mitochondrial carrier protein 1 OS=Mus musculus GN=Slc25a30 PE=1 SV=1 |
| evm.model.scaffold_11.82 | 0.47 | 541 | 479 | 1.00E-162 | Polypeptide N-acetylgalactosaminyltransferase 1 OS=Drosophila melanogaster GN=GalNAc-T1 PE=2 SV=2 |
| evm.model.scaffold_75.3 | 0.31 | 560 | 257 | 8.00E-77 | WD and tetratricopeptide repeats protein 1 OS=Homo sapiens GN=WDTC1 PE=1 SV=2 |
| evm.model.scaffold_554.44 | 0.62 | 331 | 406 | 1.00E-142 | COP9 signalosome complex subunit 5 OS=Drosophila melanogaster GN=CSN5 PE=1 SV=1 |
| evm.model.scaffold_100.19 | 0.42 | 318 | 254 | 3.00E-74 | ALK tyrosine kinase receptor OS=Homo sapiens GN=ALK PE=1 SV=3 |
| evm.model.scaffold_85.93 | 0.3 | 145 | 77.4 | 9.00E-14 | Protein jim lovell OS=Drosophila melanogaster GN=lov PE=2 SV=2 |
| evm.model.scaffold_59.234 | 0.4 | 430 | 271 | 3.00E-83 | Probable 28S rRNA (cytosine-C(5))-methyltransferase OS=Mus musculus GN=Nsun5 PE=2 SV=2 |
| evm.model.scaffold_63.9 | 0.37 | 294 | 197 | 9.00E-59 | DnaJ homolog subfamily C member 28 OS=Mus musculus GN=Dnajc28 PE=2 SV=2 |
| evm.model.scaffold_797.16 | 0.34 | 136 | 58.5 | 5.00E-09 | Stathmin-2-B OS=Xenopus laevis GN=stmn2-b PE=2 SV=1 |
| evm.model.scaffold_673.30 | 0.34 | 562 | 348 | 1.00E-103 | Protein lev-9 OS=Caenorhabditis elegans GN=lev-9 PE=1 SV=3 |
| evm.model.scaffold_1.11 | 0.48 | 1085 | 944 | 0 | Protein transport protein Sec24C OS=Homo sapiens GN=SEC24C PE=1 SV=3 |
| evm.model.scaffold_57.24 | 0.5 | 194 | 171 | 3.00E-49 | Protein hairy OS=Drosophila melanogaster GN=h PE=1 SV=2 |
| evm.model.scaffold_31.157 | 0.42 | 172 | 131 | 4.00E-34 | Protein artemis OS=Rattus norvegicus GN=Dclre1c PE=2 SV=1 |
| evm.model.scaffold_55.114 | 0.32 | 669 | 248 | 2.00E-70 | Ankyrin repeat and zinc finger domain-containing protein 1 OS=Homo sapiens GN=ANKZF1 PE=1 SV=1 |
| evm.model.scaffold_94.125 | 0.4 | 232 | 214 | 5.00E-60 | Small RNA 2'-O-methyltransferase OS=Drosophila melanogaster GN=Hen1 PE=1 SV=1 |
| evm.model.scaffold_30.19 | 0.65 | 325 | 469 | 1.00E-166 | Cathepsin L OS=Sarcophaga peregrina PE=1 SV=1 |
| evm.model.scaffold_867.53 | 0.71 | 492 | 691 | 0 | T-complex protein 1 subunit beta OS=Rattus norvegicus GN=Cct2 PE=1 SV=3 |
| evm.model.scaffold_89.95 | 0.78 | 431 | 723 | 0 | Eukaryotic translation initiation factor 3 subunit A OS=Aedes aegypti GN=eIF3-S10 PE=3 SV=1 |
| evm.model.scaffold_24.20 | 0.45 | 1056 | 810 | 0 | Diacylglycerol kinase eta OS=Drosophila melanogaster GN=CG34384 PE=2 SV=1 |
| evm.model.scaffold_890.148 | 0.26 | 403 | 113 | 2.00E-24 | PR domain zinc finger protein 5 OS=Homo sapiens GN=PRDM5 PE=1 SV=2 |
| evm.model.scaffold_93.70 | 0.46 | 726 | 691 | 0 | Spermatogenesis-associated protein 20 OS=Homo sapiens GN=SPATA20 PE=2 SV=3 |
| evm.model.scaffold_24.141 | 0.26 | 626 | 198 | 5.00E-54 | Zinc finger MYND domain-containing protein 11 OS=Homo sapiens GN=ZMYND11 PE=1 SV=2 |
| evm.model.scaffold_51.25 | 0.47 | 833 | 728 | 0 | Pre-rRNA-processing protein TSR1 homolog OS=Drosophila melanogaster GN=CG7338 PE=1 SV=1 |
| evm.model.scaffold_28.15 | 0.32 | 422 | 186 | 1.00E-53 | Mevalonate kinase OS=Arabidopsis thaliana GN=At5g27450 PE=2 SV=1 |
| evm.model.scaffold_70.9 | 0.45 | 1064 | 966 | 0 | Probable E3 ubiquitin-protein ligase HERC4 OS=Mus musculus GN=Herc4 PE=2 SV=2 |
| evm.model.scaffold_55.15 | 0.51 | 406 | 431 | 7.00E-130 | PREDICTED: uncharacterized protein LOC103314146 [Tribolium castaneum] |
| evm.model.scaffold_7.13.1 | 0.33 | 384 | 212 | 5.00E-62 | Protein SMG9 OS=Salmo salar GN=smg9 PE=2 SV=1 |
| evm.model.scaffold_544.7 | 0.4 | 147 | 103 | 2.00E-22 | Male-specific lethal 1-like 1 OS=Danio rerio GN=msl1l1 PE=2 SV=1 |
| evm.model.scaffold_94.164 | 0.62 | 435 | 585 | 0 | Nedd8-activating enzyme E1 catalytic subunit OS=Drosophila melanogaster GN=Uba3 PE=1 SV=1 |
| evm.model.scaffold_119.20 | 0.34 | 433 | 238 | 9.00E-72 | RUS1 family protein C16orf58 homolog OS=Rattus norvegicus PE=2 SV=1 |
| evm.model.scaffold_197.22 | 0.9 | 150 | 296 | 3.00E-99 | Eukaryotic translation initiation factor 5A OS=Spodoptera frugiperda GN=eIF-5A PE=2 SV=1 |
| evm.model.scaffold_608.34 | 0.6 | 352 | 444 | 1.00E-154 | Polycomb protein EED OS=Gallus gallus GN=EED PE=2 SV=1 |
| evm.model.scaffold_471.4 | 0.92 | 277 | 476 | 5.00E-162 | hypothetical protein D910_10399 [Dendroctonus ponderosae] |
| evm.model.scaffold_766.38 | 0.23 | 385 | 107 | 6.00E-23 | XK-related protein 6 OS=Homo sapiens GN=XKR6 PE=2 SV=1 |
| evm.model.scaffold_137.41 | 0.72 | 160 | 251 | 6.00E-74 | Probable protein phosphatase CG10417 OS=Drosophila melanogaster GN=CG10417 PE=1 SV=1 |
| evm.model.scaffold_37.340.1 | 0.41 | 269 | 209 | 2.00E-65 | Cathepsin L1 OS=Bos taurus GN=CTSL PE=1 SV=3 |
| evm.model.scaffold_56.17 | 0.6 | 87 | 119 | 4.00E-26 | RNA-binding protein 12 OS=Mus musculus GN=Rbm12 PE=1 SV=3 |
| evm.model.scaffold_55.32 | 0.33 | 104 | 79.7 | 2.00E-14 | Protein bric-a-brac 1 OS=Drosophila melanogaster GN=bab1 PE=2 SV=2 |
| evm.model.scaffold_916.42 | 0.85 | 80 | 141 | 4.00E-39 | Class E basic helix-loop-helix protein 22 OS=Xenopus tropicalis GN=bhlhe22 PE=2 SV=1 |
| evm.model.scaffold_890.13.1 | 0.56 | 517 | 545 | 1.00E-177 | Receptor-type tyrosine-protein phosphatase N2 OS=Macaca nemestrina GN=PTPRN2 PE=2 SV=1 |
| evm.model.scaffold_57.119 | 0.32 | 864 | 450 | 1.00E-142 | Patched domain-containing protein 3 OS=Mus musculus GN=Ptchd3 PE=1 SV=1 |
| evm.model.scaffold_81.86 | 0.35 | 637 | 420 | 1.00E-135 | Cap-specific mRNA (nucleoside-2'-O-)-methyltransferase 1 OS=Drosophila melanogaster GN=CG6379 PE=2 SV=1 |
| evm.model.scaffold_544.27 | 0.56 | 436 | 486 | 1.00E-168 | Frizzled-7-A OS=Xenopus laevis GN=fzd7-a PE=1 SV=2 |
| evm.model.scaffold_554.21 | 0.71 | 121 | 205 | 2.00E-60 | unknown [Dendroctonus ponderosae] |
| evm.model.scaffold_451.20.2 | 0.64 | 540 | 726 | 0 | Phosphoglucomutase OS=Drosophila melanogaster GN=Pgm PE=1 SV=1 |
| evm.model.scaffold_890.32 | 0.25 | 192 | 72.8 | 6.00E-12 | DDB1- and CUL4-associated factor 15 OS=Bos taurus GN=DCAF15 PE=2 SV=1 |
| evm.model.scaffold_37.162 | 0.38 | 515 | 352 | 1.00E-114 | Protein C12orf4 homolog OS=Rattus norvegicus PE=1 SV=1 |
| evm.model.scaffold_1.91 | 0.46 | 666 | 538 | 0 | Macoilin-1 OS=Danio rerio GN=tmem57a PE=2 SV=1 |
| evm.model.scaffold_99.155 | 0.44 | 800 | 651 | 0 | GTPase-activating Rap/Ran-GAP domain-like protein 3 OS=Danio rerio GN=garnl3 PE=3 SV=1 |
| evm.model.scaffold_37.174 | 0.31 | 147 | 53.5 | 1.00E-07 | Caspase OS=Drosophila melanogaster GN=Drice PE=1 SV=2 |
| evm.model.scaffold_24.198 | 0.4 | 2138 | 1594 | 0 | Dedicator of cytokinesis protein 9 OS=Homo sapiens GN=DOCK9 PE=1 SV=2 |
