# Supplementary material for: Chromosome-level genome assembly and population genomic analyses provide insights into adaptive evolution of the red turpentine beetle, Dendroctonus valens
Source: BMC Biol. 2022 Aug 24;20:190. doi: 10.1186/s12915-022-01388-y (PMC9400205; doi:10.1186/s12915-022-01388-y)
Supplement: Supplementary file 1 — Additional file 1: Table S1. Summary statistics of genome sequencing data of Dendroctonus valens. Table S2. Summary statistics of genome assembly of Dendroctonus valens. Table S3. BUSCO evaluation result for genome assembly of Dendroctonus valens. Table S4. Summary statistics of transposable elements in Dendroctonus valens genome. Table S5. Summary of gene families manually curated in Dendroctonus valens genome. Table S6. Summary statistics of genome annotation in Dendroctonus valens genome. Table S7. List of gene families that are unique in Dendroctonus valens compared to other three Coleoptera species. Table S8. Gene families that are rapidly expanded in Dendroctonus valens revealed by CAFE analysis. Table S9. Gene families that are rapidly contracted in Dendroctonus valens revealed by CAFE analysis. Table S10. List of genes that are positively selected in Dendroctonus valens revealed by codeml analysis. Table S11. Gene ontology enrichment result of positively selected genes in Dendroctonus valens. Table S12. Sampling site information for genome resequencing of geographical populations. Table S13. Summary statistics of genome resequencing data in different populations. Table S14. List of genes that undergo selective sweep in the China population compared to CAMT population. [file 12915_2022_1388_MOESM1_ESM.zip › Table S11.docx]

| **Table S11** Gene ontology enrichment result of positively selected genes in *Dendroctonus valens* | | | | | |
| --- | --- | --- | --- | --- | --- |
| **GO ID** | **GO Term** | **Category** | **Pvalue** | **AdjP** | **Gene ID** |
| GO:0006429 | leucyl-tRNA aminoacylation | BP | 0.001 | 0.289 | evm.model.scaffold_463.272,evm.model.scaffold_24.20 |
| GO:0051128 | regulation of cellular component organization | BP | 0.001 | 0.289 | evm.model.scaffold_502.57,evm.model.scaffold_599.13,evm.model.scaffold_85.93,evm.model.scaffold_797.16,evm.model.scaffold_197.22,evm.model.scaffold_55.32,evm.model.scaffold_55.152 |
| GO:0001510 | RNA methylation | BP | 0.002 | 0.289 | evm.model.scaffold_195.22,evm.model.scaffold_94.125 |
| GO:0018193 | peptidyl-amino acid modification | BP | 0.002 | 0.289 | evm.model.scaffold_79.13,evm.model.scaffold_544.7,evm.model.scaffold_197.22 |
| GO:0018205 | peptidyl-lysine modification | BP | 0.006 | 0.408 | evm.model.scaffold_544.7,evm.model.scaffold_197.22 |
| GO:0006897 | endocytosis | BP | 0.012 | 0.408 | evm.model.scaffold_87.13.1,evm.model.scaffold_85.93,evm.model.scaffold_55.32,evm.model.scaffold_55.152 |
| GO:0051049 | regulation of transport | BP | 0.013 | 0.408 | evm.model.scaffold_739.37,evm.model.scaffold_85.93,evm.model.scaffold_55.32,evm.model.scaffold_55.152 |
| GO:0008033 | tRNA processing | BP | 0.013 | 0.408 | evm.model.scaffold_296.33,evm.model.scaffold_633.27,evm.model.scaffold_24.40 |
| GO:0032508 | DNA duplex unwinding | BP | 0.015 | 0.408 | evm.model.scaffold_102.100 |
| GO:0001682 | tRNA 5'-leader removal | BP | 0.015 | 0.408 | evm.model.scaffold_296.33 |
| GO:0000184 | nuclear-transcribed mRNA catabolic process, nonsense-mediated decay | BP | 0.015 | 0.408 | evm.model.scaffold_867.86 |
| GO:0007096 | regulation of exit from mitosis | BP | 0.015 | 0.408 | evm.model.scaffold_599.13 |
| GO:0016560 | protein import into peroxisome matrix, docking | BP | 0.015 | 0.408 | evm.model.scaffold_61.28 |
| GO:0018344 | protein geranylgeranylation | BP | 0.015 | 0.408 | evm.model.scaffold_79.6 |
| GO:0043984 | histone H4-K16 acetylation | BP | 0.015 | 0.408 | evm.model.scaffold_544.7 |
| GO:0045116 | protein neddylation | BP | 0.015 | 0.408 | evm.model.scaffold_94.164 |
| GO:0006452 | translational frameshifting | BP | 0.015 | 0.408 | evm.model.scaffold_197.22 |
| GO:0045901 | positive regulation of translational elongation | BP | 0.015 | 0.408 | evm.model.scaffold_197.22 |
| GO:0045905 | positive regulation of translational termination | BP | 0.015 | 0.408 | evm.model.scaffold_197.22 |
| GO:0034470 | ncRNA processing | BP | 0.018 | 0.442 | evm.model.scaffold_296.33,evm.model.scaffold_60.10,evm.model.scaffold_633.27,evm.model.scaffold_24.40 |
| GO:0006396 | RNA processing | BP | 0.018 | 0.452 | evm.model.scaffold_296.33,evm.model.scaffold_60.10,evm.model.scaffold_195.22,evm.model.scaffold_109.39,evm.model.scaffold_633.27,evm.model.scaffold_24.40 |
| GO:0030100 | regulation of endocytosis | BP | 0.019 | 0.461 | evm.model.scaffold_85.93,evm.model.scaffold_55.32,evm.model.scaffold_55.152 |
| GO:0007626 | locomotory behavior | BP | 0.021 | 0.466 | evm.model.scaffold_53.93,evm.model.scaffold_322.30 |
| GO:0010646 | regulation of cell communication | BP | 0.021 | 0.466 | evm.model.scaffold_739.37,evm.model.scaffold_53.175,evm.model.scaffold_544.27,evm.model.scaffold_99.155,evm.model.scaffold_428.137,evm.model.scaffold_844.1 |
| GO:1902531 | regulation of intracellular signal transduction | BP | 0.021 | 0.466 | evm.model.scaffold_53.175,evm.model.scaffold_544.27,evm.model.scaffold_99.155,evm.model.scaffold_428.137,evm.model.scaffold_844.1 |
| GO:0023051 | regulation of signaling | BP | 0.022 | 0.466 | evm.model.scaffold_739.37,evm.model.scaffold_53.175,evm.model.scaffold_544.27,evm.model.scaffold_99.155,evm.model.scaffold_428.137,evm.model.scaffold_844.1 |
| GO:0032259 | methylation | BP | 0.022 | 0.466 | evm.model.scaffold_195.22,evm.model.scaffold_94.125,evm.model.scaffold_81.86 |
| GO:0034660 | ncRNA metabolic process | BP | 0.026 | 0.466 | evm.model.scaffold_296.33,evm.model.scaffold_60.10,evm.model.scaffold_463.272,evm.model.scaffold_633.27,evm.model.scaffold_24.40,evm.model.scaffold_24.20 |
| GO:0032446 | protein modification by small protein conjugation | BP | 0.030 | 0.466 | evm.model.scaffold_653.16,evm.model.scaffold_94.164 |
| GO:0016255 | attachment of GPI anchor to protein | BP | 0.03 | 0.466 | evm.model.scaffold_10.110 |
| GO:0006284 | base-excision repair | BP | 0.03 | 0.466 | evm.model.scaffold_509.51 |
| GO:0009452 | 7-methylguanosine RNA capping | BP | 0.03 | 0.466 | evm.model.scaffold_195.22 |
| GO:0018279 | protein N-linked glycosylation via asparagine | BP | 0.03 | 0.466 | evm.model.scaffold_79.13 |
| GO:0008612 | peptidyl-lysine modification to peptidyl-hypusine | BP | 0.03 | 0.466 | evm.model.scaffold_197.22 |
| GO:0006399 | tRNA metabolic process | BP | 0.031 | 0.479 | evm.model.scaffold_296.33,evm.model.scaffold_463.272,evm.model.scaffold_633.27,evm.model.scaffold_24.40,evm.model.scaffold_24.20 |
| GO:0007264 | small GTPase mediated signal transduction | BP | 0.040 | 0.510 | evm.model.scaffold_588.11,evm.model.scaffold_53.175,evm.model.scaffold_37.368,evm.model.scaffold_99.155,evm.model.scaffold_24.198,evm.model.scaffold_844.1 |
| GO:0051056 | regulation of small GTPase mediated signal transduction | BP | 0.044 | 0.510 | evm.model.scaffold_53.175,evm.model.scaffold_99.155,evm.model.scaffold_428.137,evm.model.scaffold_844.1 |
| GO:0006388 | tRNA splicing, via endonucleolytic cleavage and ligation | BP | 0.045 | 0.510 | evm.model.scaffold_633.27 |
| GO:0006004 | fucose metabolic process | BP | 0.045 | 0.510 | evm.model.scaffold_24.191 |
| GO:0005887 | integral component of plasma membrane | CC | 0.002 | 0.289 | evm.model.scaffold_53.93,evm.model.scaffold_94.189,evm.model.scaffold_85.256,evm.model.scaffold_322.30,evm.model.scaffold_739.37,evm.model.scaffold_864.54,evm.model.scaffold_82.7,evm.model.scaffold_94.101,evm.model.scaffold_85.94,evm.model.scaffold_53.120,evm.model.scaffold_393.5,evm.model.scaffold_51.127,evm.model.scaffold_365.1,evm.model.scaffold_463.260 |
| GO:0044459 | plasma membrane part | CC | 0.003 | 0.289 | evm.model.scaffold_53.93,evm.model.scaffold_94.189,evm.model.scaffold_60.10,evm.model.scaffold_463.272,evm.model.scaffold_85.256,evm.model.scaffold_322.30,evm.model.scaffold_739.37,evm.model.scaffold_20.46,evm.model.scaffold_864.54,evm.model.scaffold_99.85,evm.model.scaffold_82.7,evm.model.scaffold_94.101,evm.model.scaffold_85.94,evm.model.scaffold_53.120,evm.model.scaffold_393.5,evm.model.scaffold_51.127,evm.model.scaffold_365.1,evm.model.scaffold_463.260 |
| GO:0005886 | plasma membrane | CC | 0.003 | 0.361 | evm.model.scaffold_53.93,evm.model.scaffold_94.189,evm.model.scaffold_10.110,evm.model.scaffold_60.10,evm.model.scaffold_463.272,evm.model.scaffold_85.256,evm.model.scaffold_322.30,evm.model.scaffold_55.23,evm.model.scaffold_739.37,evm.model.scaffold_20.46,evm.model.scaffold_864.54,evm.model.scaffold_99.85,evm.model.scaffold_82.7,evm.model.scaffold_94.101,evm.model.scaffold_85.94,evm.model.scaffold_53.120,evm.model.scaffold_393.5,evm.model.scaffold_51.127,evm.model.scaffold_365.1,evm.model.scaffold_463.260 |
| GO:0071944 | cell periphery | CC | 0.006 | 0.408 | evm.model.scaffold_53.93,evm.model.scaffold_94.189,evm.model.scaffold_10.110,evm.model.scaffold_60.10,evm.model.scaffold_463.272,evm.model.scaffold_85.256,evm.model.scaffold_322.30,evm.model.scaffold_55.23,evm.model.scaffold_739.37,evm.model.scaffold_20.46,evm.model.scaffold_864.54,evm.model.scaffold_99.85,evm.model.scaffold_82.7,evm.model.scaffold_94.101,evm.model.scaffold_85.94,evm.model.scaffold_181.18,evm.model.scaffold_53.120,evm.model.scaffold_393.5,evm.model.scaffold_51.127,evm.model.scaffold_771.42,evm.model.scaffold_365.1,evm.model.scaffold_463.260,evm.model.scaffold_554.21 |
| GO:0005664 | nuclear origin of replication recognition complex | CC | 0.015 | 0.408 | evm.model.scaffold_117.35 |
| GO:0072487 | MSL complex | CC | 0.015 | 0.408 | evm.model.scaffold_544.7 |
| GO:0001518 | voltage-gated sodium channel complex | CC | 0.016 | 0.408 | evm.model.scaffold_85.94,evm.model.scaffold_393.5 |
| GO:0008250 | oligosaccharyltransferase complex | CC | 0.03 | 0.466 | evm.model.scaffold_79.13 |
| GO:0030127 | COPII vesicle coat | CC | 0.03 | 0.466 | evm.model.scaffold_1.11 |
| GO:0043198 | dendritic shaft | CC | 0.045 | 0.510 | evm.model.scaffold_739.37 |
| GO:0008173 | RNA methyltransferase activity | MF | 0.001 | 0.289 | evm.model.scaffold_109.39,evm.model.scaffold_94.125,evm.model.scaffold_162.9 |
| GO:0004823 | leucine-tRNA ligase activity | MF | 0.001 | 0.289 | evm.model.scaffold_463.272,evm.model.scaffold_24.20 |
| GO:0004549 | tRNA-specific ribonuclease activity | MF | 0.001 | 0.289 | evm.model.scaffold_296.33,evm.model.scaffold_633.27 |
| GO:0008641 | small protein activating enzyme activity | MF | 0.002 | 0.289 | evm.model.scaffold_53.46,evm.model.scaffold_94.164 |
| GO:0005515 | protein binding | MF | 0.011 | 0.408 | evm.model.scaffold_554.4.2,evm.model.scaffold_53.93,evm.model.scaffold_162.8,evm.model.scaffold_296.33,evm.model.scaffold_60.10,evm.model.scaffold_24.235.4,evm.model.scaffold_588.11,evm.model.scaffold_108.31.1,evm.model.scaffold_37.27,evm.model.scaffold_608.89,evm.model.scaffold_882.18,evm.model.scaffold_85.165,evm.model.scaffold_739.14,evm.model.scaffold_94.50,evm.model.scaffold_867.51,evm.model.scaffold_55.140,evm.model.scaffold_85.84,evm.model.scaffold_647.100,evm.model.scaffold_94.39.1,evm.model.scaffold_14.49,evm.model.scaffold_20.46,evm.model.scaffold_749.46.1,evm.model.scaffold_844.7,evm.model.scaffold_864.54,evm.model.scaffold_463.163,evm.model.scaffold_24.45,evm.model.scaffold_633.30,evm.model.scaffold_82.7,evm.model.scaffold_32.10,evm.model.scaffold_615.24,evm.model.scaffold_818.53.1,evm.model.scaffold_882.25,evm.model.scaffold_766.30,evm.model.scaffold_524.32,evm.model.scaffold_365.1,evm.model.scaffold_728.10,evm.model.scaffold_451.21,evm.model.scaffold_37.195,evm.model.scaffold_53.46,evm.model.scaffold_822.16,evm.model.scaffold_818.24,evm.model.scaffold_37.234,evm.model.scaffold_79.6,evm.model.scaffold_75.3,evm.model.scaffold_554.44,evm.model.scaffold_85.93,evm.model.scaffold_59.234,evm.model.scaffold_57.24,evm.model.scaffold_55.114,evm.model.scaffold_867.53,evm.model.scaffold_89.95,evm.model.scaffold_24.20,evm.model.scaffold_7.13.1,evm.model.scaffold_197.22,evm.model.scaffold_608.34,evm.model.scaffold_137.41,evm.model.scaffold_55.32,evm.model.scaffold_916.42,evm.model.scaffold_544.27,evm.model.scaffold_24.198 |
| GO:0016741 | transferase activity, transferring one-carbon groups | MF | 0.012 | 0.408 | evm.model.scaffold_195.22,evm.model.scaffold_109.39,evm.model.scaffold_94.125,evm.model.scaffold_890.148,evm.model.scaffold_81.86,evm.model.scaffold_162.9 |
| GO:0004135 | amylo-alpha-1,6-glucosidase activity | MF | 0.015 | 0.408 | evm.model.scaffold_116.28 |
| GO:0016428 | tRNA (cytosine-5-)-methyltransferase activity | MF | 0.015 | 0.408 | evm.model.scaffold_162.9 |
| GO:0004842 | ubiquitin-protein transferase activity | MF | 0.016 | 0.408 | evm.model.scaffold_14.49,evm.model.scaffold_653.16,evm.model.scaffold_882.3,evm.model.scaffold_70.9 |
| GO:0005248 | voltage-gated sodium channel activity | MF | 0.016 | 0.408 | evm.model.scaffold_85.94,evm.model.scaffold_393.5 |
| GO:0004521 | endoribonuclease activity | MF | 0.016 | 0.408 | evm.model.scaffold_296.33,evm.model.scaffold_633.27 |
| GO:0008168 | methyltransferase activity | MF | 0.021 | 0.466 | evm.model.scaffold_195.22,evm.model.scaffold_109.39,evm.model.scaffold_94.125,evm.model.scaffold_81.86,evm.model.scaffold_162.9 |
| GO:0004526 | ribonuclease P activity | MF | 0.03 | 0.466 | evm.model.scaffold_296.33 |
| GO:0005135 | interleukin-3 receptor binding | MF | 0.03 | 0.466 | evm.model.scaffold_108.31.1 |
| GO:0000213 | tRNA-intron endonuclease activity | MF | 0.03 | 0.466 | evm.model.scaffold_633.27 |
| GO:0004496 | mevalonate kinase activity | MF | 0.03 | 0.466 | evm.model.scaffold_28.15 |
| GO:0043022 | ribosome binding | MF | 0.03 | 0.466 | evm.model.scaffold_197.22 |
| GO:0016779 | nucleotidyltransferase activity | MF | 0.035 | 0.510 | evm.model.scaffold_53.36,evm.model.scaffold_51.159,evm.model.scaffold_119.39,evm.model.scaffold_882.3,evm.model.scaffold_197.22,evm.model.scaffold_162.9 |
| GO:0004993 | serotonin receptor activity | MF | 0.037 | 0.510 | evm.model.scaffold_53.93,evm.model.scaffold_85.256,evm.model.scaffold_322.30,evm.model.scaffold_739.37,evm.model.scaffold_463.260 |
| GO:0008047 | enzyme activator activity | MF | 0.040 | 0.510 | evm.model.scaffold_615.24,evm.model.scaffold_53.46,evm.model.scaffold_94.164,evm.model.scaffold_544.27,evm.model.scaffold_99.155,evm.model.scaffold_428.137 |
| GO:0008864 | formyltetrahydrofolate deformylase activity | MF | 0.045 | 0.510 | evm.model.scaffold_85.165 |
| GO:0004176 | ATP-dependent peptidase activity | MF | 0.045 | 0.510 | evm.model.scaffold_94.116 |
| GO:0004579 | dolichyl-diphosphooligosaccharide-protein glycotransferase activity | MF | 0.045 | 0.510 | evm.model.scaffold_79.13 |
| GO:0004375 | glycine dehydrogenase (decarboxylating) activity | MF | 0.045 | 0.510 | evm.model.scaffold_554.27 |
| GO:0034450 | ubiquitin-ubiquitin ligase activity | MF | 0.045 | 0.510 | evm.model.scaffold_653.16 |
| GO:0005283 | sodium:amino acid symporter activity | MF | 0.047 | 0.516 | evm.model.scaffold_82.7,evm.model.scaffold_61.28 |
| GO:0005085 | guanyl-nucleotide exchange factor activity | MF | 0.048 | 0.516 | evm.model.scaffold_53.175,evm.model.scaffold_24.198,evm.model.scaffold_844.1 |
