# Supplementary material for: Chromosome-level genome assembly and population genomic analyses provide insights into adaptive evolution of the red turpentine beetle, Dendroctonus valens
Source: BMC Biol. 2022 Aug 24;20:190. doi: 10.1186/s12915-022-01388-y (PMC9400205; doi:10.1186/s12915-022-01388-y)
Supplement: Supplementary file 1 — Additional file 1: Table S1. Summary statistics of genome sequencing data of Dendroctonus valens. Table S2. Summary statistics of genome assembly of Dendroctonus valens. Table S3. BUSCO evaluation result for genome assembly of Dendroctonus valens. Table S4. Summary statistics of transposable elements in Dendroctonus valens genome. Table S5. Summary of gene families manually curated in Dendroctonus valens genome. Table S6. Summary statistics of genome annotation in Dendroctonus valens genome. Table S7. List of gene families that are unique in Dendroctonus valens compared to other three Coleoptera species. Table S8. Gene families that are rapidly expanded in Dendroctonus valens revealed by CAFE analysis. Table S9. Gene families that are rapidly contracted in Dendroctonus valens revealed by CAFE analysis. Table S10. List of genes that are positively selected in Dendroctonus valens revealed by codeml analysis. Table S11. Gene ontology enrichment result of positively selected genes in Dendroctonus valens. Table S12. Sampling site information for genome resequencing of geographical populations. Table S13. Summary statistics of genome resequencing data in different populations. Table S14. List of genes that undergo selective sweep in the China population compared to CAMT population. [file 12915_2022_1388_MOESM1_ESM.zip › Table S14.docx]

| **Table** **S14** List of genes that undergo selective sweep in the China population compared to CAMT population | | | | | |
| --- | --- | --- | --- | --- | --- |
| **Gene ID** | **Identity** | **Aligned length** | **Score** | **E-value** | **Description** |
| evm.model.scaffold_0.4 | 0.33 | 315 | 181 | 1.00E-46 | Heterogeneous nuclear ribonucleoprotein U OS=Homo sapiens GN=HNRNPU PE=1 SV=6 |
| evm.model.scaffold_1.1 | 0.27 | 1778 | 568 | 1.00E-160 | Muscle M-line assembly protein unc-89 OS=Caenorhabditis elegans GN=unc-89 PE=1 SV=3 |
| evm.model.scaffold_1.30 | 0.23 | 252 | 78.2 | 3.00E-15 | Lysosomal-associated transmembrane protein 4B OS=Rattus norvegicus GN=Laptm4b PE=2 SV=1 |
| evm.model.scaffold_10.118 | 0.91 | 150 | 283 | 1.00E-87 | hypothetical protein D910_08855, partial [Dendroctonus ponderosae] |
| evm.model.scaffold_10.39 | 0.48 | 308 | 310 | 1.00E-103 | Acyl-CoA Delta(11) desaturase OS=Trichoplusia ni GN=D11DS PE=1 SV=2 |
| evm.model.scaffold_100.59 | 0.22 | 336 | 123 | 2.00E-28 | WD repeat-containing protein 34 OS=Homo sapiens GN=WDR34 PE=1 SV=2 |
| evm.model.scaffold_100.6 | 0.4 | 134 | 97.8 | 3.00E-21 | Neural proliferation differentiation and control protein 1 OS=Homo sapiens GN=NPDC1 PE=1 SV=2 |
| evm.model.scaffold_102.2 | 0.46 | 763 | 658 | 0 | B-box type zinc finger protein ncl-1 OS=Caenorhabditis elegans GN=ncl-1 PE=2 SV=1 |
| evm.model.scaffold_104.103.1 | 0.34 | 428 | 218 | 2.00E-56 | Sorbin and SH3 domain-containing protein 1 OS=Mus musculus GN=Sorbs1 PE=1 SV=2 |
| evm.model.scaffold_104.32 | 0.95 | 231 | 467 | 7.00E-156 | hypothetical protein YQE_10997, partial [Dendroctonus ponderosae] |
| evm.model.scaffold_104.5 | 0.47 | 4266 | 3685 | 0 | E3 ubiquitin-protein ligase UBR4 OS=Homo sapiens GN=UBR4 PE=1 SV=1 |
| evm.model.scaffold_104.89 | 0.46 | 249 | 246 | 2.00E-79 | Tetraspanin-5 OS=Bos taurus GN=TSPAN5 PE=2 SV=1 |
| evm.model.scaffold_107.42 | 0.83 | 253 | 431 | 6.00E-149 | hypothetical protein D910_11821 [Dendroctonus ponderosae] |
| evm.model.scaffold_107.61 | 0.39 | 268 | 178 | 2.00E-49 | Raf homolog serine/threonine-protein kinase phl OS=Drosophila melanogaster GN=phl PE=1 SV=6 |
| evm.model.scaffold_107.72 | 0.35 | 1386 | 823 | 0 | Nuclear pore complex protein Nup155 OS=Mus musculus GN=Nup155 PE=1 SV=1 |
| evm.model.scaffold_108.1 | 0.38 | 233 | 117 | 1.00E-27 | hypothetical protein TcasGA2_TC005430 [Tribolium castaneum] |
| evm.model.scaffold_110.11 | 0.64 | 177 | 240 | 3.00E-80 | Phosphatidylglycerophosphatase and protein-tyrosine phosphatase 1 OS=Drosophila melanogaster GN=Plip PE=2 SV=1 |
| evm.model.scaffold_110.15 | 0.51 | 314 | 355 | 1.00E-119 | Mitochondrial tRNA-specific 2-thiouridylase 1 OS=Drosophila melanogaster GN=CG3021 PE=2 SV=3 |
| evm.model.scaffold_110.19 | 0.33 | 242 | 117 | 8.00E-28 | GB17449; uncharacterized LOC412835; K14313 nuclear pore complex protein Nup53 (A) |
| evm.model.scaffold_111.31 | 0.89 | 140 | 256 | 5.00E-84 | hypothetical protein YQE_03879, partial [Dendroctonus ponderosae] |
| evm.model.scaffold_111.33 | 0.87 | 685 | 1213 | 0 | Neural-cadherin OS=Drosophila melanogaster GN=CadN PE=1 SV=2 |
| evm.model.scaffold_111.36 | 0.36 | 518 | 333 | 9.00E-99 | Run domain Beclin-1 interacting and cysteine-rich containing protein OS=Mus musculus GN=Kiaa0226 PE=1 SV=2 |
| evm.model.scaffold_112.4 | 0.34 | 257 | 60.1 | 4.00E-06 | hypothetical protein TcasGA2_TC007800 [Tribolium castaneum] |
| evm.model.scaffold_112.7 | 0.49 | 455 | 449 | 1.00E-151 | Vesicular glutamate transporter 2 OS=Homo sapiens GN=SLC17A6 PE=2 SV=1 |
| evm.model.scaffold_114.7 | 0.38 | 592 | 390 | 1.00E-126 | Heat shock 70 kDa protein cognate 4 OS=Drosophila melanogaster GN=Hsc70-4 PE=1 SV=3 |
| evm.model.scaffold_116.16 | 0.41 | 763 | 622 | 0 | Gamma-aminobutyric acid type B receptor subunit 2 OS=Homo sapiens GN=GABBR2 PE=1 SV=1 |
| evm.model.scaffold_116.41.3 | 0.27 | 834 | 273 | 6.00E-78 | Sphingomyelin phosphodiesterase 4 OS=Mus musculus GN=Smpd4 PE=2 SV=2 |
| evm.model.scaffold_116.42 | 0.34 | 347 | 198 | 3.00E-53 | A-kinase anchor protein 1, mitochondrial OS=Rattus norvegicus GN=Akap1 PE=2 SV=1 |
| evm.model.scaffold_116.43 | 0.29 | 712 | 273 | 2.00E-78 | Histone H2A deubiquitinase MYSM1 OS=Homo sapiens GN=MYSM1 PE=1 SV=1 |
| evm.model.scaffold_116.45 | 0.34 | 322 | 175 | 1.00E-50 | Interferon-inducible double-stranded RNA-dependent protein kinase activator A homolog A OS=Xenopus laevis GN=prkra-a PE=2 SV=1 |
| evm.model.scaffold_116.46 | 0.22 | 586 | 59.3 | 2.00E-07 | Serine/threonine-protein phosphatase 6 regulatory ankyrin repeat subunit B OS=Homo sapiens GN=ANKRD44 PE=1 SV=3 |
| evm.model.scaffold_116.70 | 0.37 | 67 | 65.5 | 5.00E-10 | SH3 and cysteine-rich domain-containing protein 2 OS=Homo sapiens GN=STAC2 PE=1 SV=1 |
| evm.model.scaffold_116.93.3 | 0.51 | 653 | 590 | 0 | Serine/threonine-protein kinase tousled-like 1 OS=Mus musculus GN=Tlk1 PE=2 SV=2 |
| evm.model.scaffold_13.36 | 0.90 | 118 | 223 | 7.00E-71 | hypothetical protein YQE_09620, partial [Dendroctonus ponderosae] |
| evm.model.scaffold_131.12 | 0.59 | 365 | 465 | 1.00E-161 | Septin-4 OS=Macaca fascicularis GN=SEPT4 PE=2 SV=1 |
| evm.model.scaffold_14.64 | 0.32 | 794 | 437 | 1.00E-140 | Inactive dipeptidyl peptidase 10 OS=Homo sapiens GN=DPP10 PE=1 SV=2 |
| evm.model.scaffold_143.1 | 0.43 | 286 | 213 | 2.00E-62 | Paired box protein Pax-6 OS=Coturnix coturnix japonica GN=PAX6 PE=2 SV=1 |
| evm.model.scaffold_143.9 | 0.5 | 188 | 166 | 3.00E-46 | ATP-binding cassette sub-family G member 4 OS=Homo sapiens GN=ABCG4 PE=1 SV=2 |
| evm.model.scaffold_156.10 | 0.31 | 174 | 63.9 | 2.00E-09 | Nephrin OS=Rattus norvegicus GN=Nphs1 PE=1 SV=2 |
| evm.model.scaffold_16.14_evm.model.scaffold_16.13 | 0.55 | 349 | 351 | 1.00E-119 | Zinc transporter ZIP11 OS=Mus musculus GN=Slc39a11 PE=2 SV=1 |
| evm.model.scaffold_164.6 | 0.39 | 415 | 284 | 4.00E-91 | Orexin receptor type 2 OS=Rattus norvegicus GN=Hcrtr2 PE=2 SV=1 |
| evm.model.scaffold_169.1 | 0.79 | 120 | 224 | 2.00E-70 | hypothetical protein YQE_06570, partial [Dendroctonus ponderosae] |
| evm.model.scaffold_189.17 | 0.32 | 305 | 219 | 5.00E-59 | Putative sodium-coupled neutral amino acid transporter 10 OS=Mus musculus GN=Slc38a10 PE=2 SV=2 |
| evm.model.scaffold_19.3 | 0.42 | 339 | 298 | 1.00E-98 | Protein Wnt-6 OS=Homo sapiens GN=WNT6 PE=1 SV=2 |
| evm.model.scaffold_192.3 | NA | NA | NA | NA | No blast hit |
| evm.model.scaffold_194.3 | 0.21 | 247 | 52 | 7.00E-06 | Cuticlin-1 OS=Caenorhabditis elegans GN=cut-1 PE=2 SV=2 |
| evm.model.scaffold_197.2 | 0.23 | 339 | 65.5 | 2.00E-09 | Formin-like protein 1 OS=Homo sapiens GN=FMNL1 PE=1 SV=3 |
| evm.model.scaffold_20.44 | 0.82 | 588 | 1029 | 0 | Pre-mRNA-splicing factor ATP-dependent RNA helicase DHX15 OS=Pongo abelii GN=DHX15 PE=2 SV=2 |
| evm.model.scaffold_207.1 | 0.22 | 414 | 61.2 | 8.00E-09 | Organic cation/carnitine transporter 7 OS=Arabidopsis thaliana GN=OCT7 PE=2 SV=1 |
| evm.model.scaffold_21.37 | 0.54 | 116 | 113 | 9.00E-26 | Neurogenic differentiation factor 1 OS=Rattus norvegicus GN=Neurod1 PE=1 SV=1 |
| evm.model.scaffold_214.24 | 0.32 | 399 | 204 | 2.00E-59 | Sialin OS=Ovis aries GN=SLC17A5 PE=2 SV=1 |
| evm.model.scaffold_215.102 | NA | NA | NA | NA | No blast hit |
| evm.model.scaffold_215.127 | 0.35 | 558 | 359 | 1.00E-106 | MutS protein homolog 4 OS=Mus musculus GN=Msh4 PE=2 SV=1 |
| evm.model.scaffold_215.147 | 0.83 | 97 | 168 | 3.00E-54 | 40S ribosomal protein S26 OS=Anopheles gambiae GN=RpS26 PE=3 SV=2 |
| evm.model.scaffold_215.148 | 0.66 | 53 | 67.8 | 3.00E-14 | PEST proteolytic signal-containing nuclear protein OS=Rattus norvegicus GN=Pcnp PE=2 SV=1 |
| evm.model.scaffold_215.156 | 0.43 | 1125 | 855 | 0 | Lethal(2) giant larvae protein OS=Drosophila melanogaster GN=l(2)gl PE=1 SV=2 |
| evm.model.scaffold_215.46 | 0.48 | 241 | 201 | 4.00E-55 | Dual 3',5'-cyclic-AMP and -GMP phosphodiesterase 11 OS=Drosophila melanogaster GN=Pde11 PE=1 SV=4 |
| evm.model.scaffold_215.53 | 0.46 | 797 | 625 | 0 | RING finger protein unkempt homolog OS=Homo sapiens GN=UNK PE=1 SV=2 |
| evm.model.scaffold_22.110 | 0.53 | 132 | 169 | 7.00E-42 | Caskin-1 OS=Rattus norvegicus GN=Caskin1 PE=1 SV=1 |
| evm.model.scaffold_227.2 | 0.61 | 426 | 575 | 0 | Protein mesh OS=Bombyx mori PE=1 SV=1 |
| evm.model.scaffold_23.64 | 0.34 | 180 | 107 | 2.00E-24 | Peroxidase OS=Drosophila melanogaster GN=Pxd PE=2 SV=2 |
| evm.model.scaffold_234.1 | 0.31 | 157 | 83.6 | 1.00E-16 | Unconventional myosin-Ia OS=Gallus gallus GN=MYO1A PE=1 SV=2 |
| evm.model.scaffold_234.11 | 0.49 | 669 | 649 | 0 | UPF0668 protein C10orf76 OS=Homo sapiens GN=C10orf76 PE=2 SV=1 |
| evm.model.scaffold_24.224 | NA | NA | NA | NA | No blast hit |
| evm.model.scaffold_242.24 | 0.44 | 4559 | 3571 | 0 | E3 ubiquitin-protein ligase HERC2 OS=Homo sapiens GN=HERC2 PE=1 SV=2 |
| evm.model.scaffold_25.10 | 0.97 | 541 | 1012 | 0 | hypothetical protein YQE_05803, partial [Dendroctonus ponderosae] |
| evm.model.scaffold_251.18.1 | 0.54 | 355 | 415 | 1.00E-120 | SH3 and multiple ankyrin repeat domains protein 3 OS=Homo sapiens GN=SHANK3 PE=1 SV=3 |
| evm.model.scaffold_260.14 | NA | NA | NA | NA | No blast hit |
| evm.model.scaffold_265.17 | 0.23 | 339 | 65.5 | 2.00E-09 | Formin-like protein 1 OS=Homo sapiens GN=FMNL1 PE=1 SV=3 |
| evm.model.scaffold_27.6 | 0.26 | 377 | 151 | 6.00E-39 | Importin-13 OS=Gallus gallus GN=IPO13 PE=2 SV=1 |
| evm.model.scaffold_283.11 | 0.26 | 364 | 82.4 | 4.00E-15 | Cysteine-rich motor neuron 1 protein OS=Homo sapiens GN=CRIM1 PE=1 SV=1 |
| evm.model.scaffold_283.19 | 0.32 | 546 | 284 | 2.00E-88 | Sensory neuron membrane protein 2 OS=Drosophila melanogaster GN=Snmp2 PE=2 SV=1 |
| evm.model.scaffold_283.28 | NA | NA | NA | NA | No blast hit |
| evm.model.scaffold_285.26 | 0.32 | 283 | 145 | 9.00E-38 | Polycomb group RING finger protein 2 OS=Mus musculus GN=Pcgf2 PE=1 SV=2 |
| evm.model.scaffold_285.7 | 0.29 | 123 | 48.5 | 3.00E-05 | Cell adhesion molecule 1 OS=Homo sapiens GN=CADM1 PE=1 SV=2 |
| evm.model.scaffold_299.12 | 0.5 | 102 | 95.5 | 6.00E-25 | Cardioactive peptide OS=Manduca sexta GN=CCAP PE=1 SV=1 |
| evm.model.scaffold_299.7 | 0.27 | 189 | 80.5 | 2.00E-17 | Gamma-interferon-inducible lysosomal thiol reductase OS=Mus musculus GN=Ifi30 PE=1 SV=3 |
| evm.model.scaffold_3.59 | 0.36 | 324 | 212 | 3.00E-54 | Nuclear receptor coactivator 2 OS=Rattus norvegicus GN=Ncoa2 PE=1 SV=1 |
| evm.model.scaffold_3.66 | 0.22 | 244 | 59.7 | 4.00E-08 | Growth factor receptor-bound protein 14 OS=Homo sapiens GN=GRB14 PE=1 SV=2 |
| evm.model.scaffold_30.12 | 0.78 | 319 | 475 | 5.00E-165 | hypothetical protein D910_03865 [Dendroctonus ponderosae] |
| evm.model.scaffold_309.24 | 0.37 | 198 | 129 | 3.00E-29 | WW domain-containing protein tag-325 OS=Caenorhabditis elegans GN=tag-325 PE=3 SV=1 |
| evm.model.scaffold_309.4 | 0.38 | 264 | 135 | 1.00E-37 | Eukaryotic translation initiation factor 4H OS=Mus musculus GN=Eif4h PE=1 SV=3 |
| evm.model.scaffold_309.6 | 0.43 | 608 | 506 | 1.00E-157 | Glucose dehydrogenase [FAD, quinone] OS=Drosophila pseudoobscura pseudoobscura GN=Gld PE=3 SV=4 |
| evm.model.scaffold_31.20 | 0.89 | 535 | 976 | 0 | hypothetical protein D910_07448 [Dendroctonus ponderosae] |
| evm.model.scaffold_31.200.1 | 0.26 | 282 | 85.5 | 1.00E-16 | Sex peptide receptor OS=Drosophila melanogaster GN=SPR PE=1 SV=1 |
| evm.model.scaffold_31.208 | 0.72 | 369 | 553 | 0 | hypothetical protein YQE_04363, partial [Dendroctonus ponderosae] |
| evm.model.scaffold_31.228 | 0.53 | 121 | 150 | 3.00E-40 | Biorientation of chromosomes in cell division protein 1 OS=Rattus norvegicus GN=Bod1 PE=2 SV=1 |
| evm.model.scaffold_31.240 | 0.57 | 754 | 828 | 0 | Potassium voltage-gated channel subfamily H member 8 OS=Mus musculus GN=Kcnh8 PE=2 SV=2 |
| evm.model.scaffold_31.50 | 0.37 | 232 | 118 | 2.00E-30 | Retinol dehydrogenase 13 OS=Homo sapiens GN=RDH13 PE=1 SV=2 |
| evm.model.scaffold_31.92.7 | 0.45 | 531 | 500 | 1.00E-171 | Sodium- and chloride-dependent GABA transporter 1 OS=Rattus norvegicus GN=Slc6a1 PE=1 SV=1 |
| evm.model.scaffold_315.17 | 0.48 | 229 | 237 | 5.00E-76 | Putative endoglucanase type K OS=Fusarium oxysporum PE=2 SV=1 |
| evm.model.scaffold_32.46.2 | 0.93 | 723 | 1268 | 0 | hypothetical protein YQE_00963, partial [Dendroctonus ponderosae] |
| evm.model.scaffold_328.1 | 0.49 | 93 | 84.7 | 3.00E-18 | Decaprenyl-diphosphate synthase subunit 2 OS=Homo sapiens GN=PDSS2 PE=1 SV=2 |
| evm.model.scaffold_33.2 | 0.29 | 334 | 110 | 5.00E-24 | Leucine-rich repeat-containing protein 24 OS=Homo sapiens GN=LRRC24 PE=2 SV=2 |
| evm.model.scaffold_35.55 | 0.23 | 237 | 57 | 2.00E-07 | UPF0392 protein F13G3.3 OS=Caenorhabditis elegans GN=F13G3.3 PE=3 SV=2 |
| evm.model.scaffold_359.5 | NA | NA | NA | NA | No blast hit |
| evm.model.scaffold_367.1 | 0.22 | 371 | 68.6 | 1.00E-10 | Coiled-coil domain-containing protein 142 OS=Mus musculus GN=Ccdc142 PE=2 SV=2 |
| evm.model.scaffold_37.122 | NA | NA | NA | NA | No blast hit |
| evm.model.scaffold_37.125 | 0.35 | 1168 | 663 | 0 | Nuclear pore complex protein Nup205 OS=Homo sapiens GN=NUP205 PE=1 SV=3 |
| evm.model.scaffold_37.133 | 0.53 | 618 | 652 | 0 | Plastin-2 OS=Mus musculus GN=Lcp1 PE=1 SV=4 |
| evm.model.scaffold_37.137 | 0.45 | 749 | 557 | 1.00E-179 | La-related protein 1 OS=Mus musculus GN=Larp1 PE=1 SV=3 |
| evm.model.scaffold_37.139 | 0.37 | 1096 | 696 | 0 | Down syndrome cell adhesion molecule-like protein Dscam2 OS=Drosophila melanogaster GN=Dscam2 PE=2 SV=3 |
| evm.model.scaffold_37.146 | 0.44 | 561 | 438 | 1.00E-137 | Lysine-specific demethylase 6A OS=Mus musculus GN=Kdm6a PE=1 SV=2 |
| evm.model.scaffold_37.159 | 0.67 | 2861 | 3707 | 0 | Inositol 1,4,5-trisphosphate receptor OS=Drosophila melanogaster GN=Itp-r83A PE=2 SV=3 |
| evm.model.scaffold_37.167 | 0.56 | 787 | 891 | 0 | Neuroglian OS=Drosophila melanogaster GN=Nrg PE=1 SV=2 |
| evm.model.scaffold_37.169 | 0.21 | 420 | 60.5 | 3.00E-07 | zinc finger protein 271-like; K09228 KRAB domain-containing zinc finger protein (A) |
| evm.model.scaffold_37.17 | 0.18 | 408 | 59.3 | 4.00E-08 | Nucleoporin NUP188 homolog OS=Xenopus tropicalis GN=nup188 PE=2 SV=1 |
| evm.model.scaffold_37.2 | 0.62 | 752 | 953 | 0 | Metabotropic glutamate receptor OS=Drosophila melanogaster GN=mGluR PE=1 SV=2 |
| evm.model.scaffold_37.206 | 0.46 | 1108 | 918 | 0 | Nipped-B-like protein OS=Homo sapiens GN=NIPBL PE=1 SV=2 |
| evm.model.scaffold_37.229 | 0.46 | 231 | 191 | 1.00E-55 | Protein tipE OS=Drosophila melanogaster GN=tipE PE=2 SV=1 |
| evm.model.scaffold_37.273 | 0.44 | 72 | 60.5 | 1.00E-08 | Grainyhead-like protein 2 homolog OS=Xenopus tropicalis GN=grhl2 PE=2 SV=1 |
| evm.model.scaffold_37.284 | 0.6 | 329 | 421 | 1.00E-144 | TBC1 domain family member 22B OS=Homo sapiens GN=TBC1D22B PE=1 SV=3 |
| evm.model.scaffold_37.285 | 0.43 | 530 | 459 | 1.00E-155 | Tetratricopeptide repeat protein 39B OS=Xenopus laevis GN=ttc39b PE=2 SV=1 |
| evm.model.scaffold_37.297 | 0.24 | 191 | 57 | 5.00E-09 | Mid1-interacting protein 1-like OS=Danio rerio GN=mid1ip1l PE=2 SV=1 |
| evm.model.scaffold_37.298 | 0.34 | 232 | 137 | 1.00E-34 | Protein eva-1 homolog C OS=Mus musculus GN=Eva1c PE=2 SV=2 |
| evm.model.scaffold_37.304 | NA | NA | NA | NA | No blast hit |
| evm.model.scaffold_37.305 | 0.3 | 362 | 124 | 9.00E-30 | Golgin subfamily A member 5 OS=Xenopus laevis GN=golga5 PE=2 SV=1 |
| evm.model.scaffold_37.311 | 0.46 | 460 | 377 | 1.00E-118 | Protein disulfide-isomerase A3 OS=Gallus gallus GN=PDIA3 PE=2 SV=1 |
| evm.model.scaffold_37.312 | 0.85 | 355 | 651 | 0 | Arginine kinase OS=Plodia interpunctella GN=ARGK PE=1 SV=1 |
| evm.model.scaffold_37.313 | 0.27 | 152 | 52.4 | 3.00E-07 | Filamentous hemagglutinin OS=Bordetella pertussis (strain Tohama I / ATCC BAA-589 / NCTC 13251) GN=fhaB PE=1 SV=4 |
| evm.model.scaffold_37.325 | 0.46 | 108 | 97.1 | 2.00E-19 | SAFB-like transcription modulator OS=Mus musculus GN=Sltm PE=1 SV=1 |
| evm.model.scaffold_37.329 | 0.69 | 413 | 582 | 0 | Protein lin-10 OS=Caenorhabditis elegans GN=lin-10 PE=1 SV=1 |
| evm.model.scaffold_37.338 | 0.57 | 1383 | 1539 | 0 | Kinesin-like protein KIF13A OS=Mus musculus GN=Kif13a PE=1 SV=1 |
| evm.model.scaffold_37.340.1 | 0.41 | 269 | 209 | 2.00E-65 | Cathepsin L1 OS=Bos taurus GN=CTSL PE=1 SV=3 |
| evm.model.scaffold_37.342 | 0.38 | 442 | 302 | 4.00E-90 | Nischarin OS=Homo sapiens GN=NISCH PE=1 SV=3 |
| evm.model.scaffold_37.347 | 0.58 | 662 | 691 | 0 | Aryl hydrocarbon receptor nuclear translocator homolog OS=Drosophila melanogaster GN=tgo PE=1 SV=3 |
| evm.model.scaffold_37.350 | 0.32 | 497 | 263 | 3.00E-71 | Atrial natriuretic peptide-converting enzyme OS=Homo sapiens GN=CORIN PE=1 SV=2 |
| evm.model.scaffold_37.353 | 0.48 | 566 | 466 | 1.00E-157 | Ubiquilin-1 OS=Homo sapiens GN=UBQLN1 PE=1 SV=2 |
| evm.model.scaffold_37.354 | 0.99 | 388 | 786 | 0 | hypothetical protein YQE_09247, partial [Dendroctonus ponderosae] |
| evm.model.scaffold_37.49 | 0.72 | 507 | 774 | 0 | Inosine-5'-monophosphate dehydrogenase OS=Drosophila melanogaster GN=ras PE=1 SV=1 |
| evm.model.scaffold_37.62 | 0.45 | 242 | 204 | 4.00E-63 | Very-long-chain 3-oxoacyl-CoA reductase OS=Xenopus tropicalis GN=hsd17b12 PE=2 SV=1 |
| evm.model.scaffold_37.74 | 0.85 | 309 | 556 | 0 | Steroid receptor seven-up, isoforms B/C OS=Drosophila melanogaster GN=svp PE=2 SV=1 |
| evm.model.scaffold_379.2 | 0.32 | 235 | 113 | 4.00E-27 | Neuropeptide FF receptor 1 OS=Rattus norvegicus GN=Npffr1 PE=1 SV=1 |
| evm.model.scaffold_38.2.1 | 0.68 | 138 | 193 | 7.00E-57 | Homeotic protein Sex combs reduced OS=Drosophila melanogaster GN=Scr PE=1 SV=5 |
| evm.model.scaffold_390.93 | 0.69 | 116 | 129 | 5.00E-35 | Vesicle transport protein GOT1B OS=Mus musculus GN=Golt1b PE=2 SV=1 |
| evm.model.scaffold_390.96 | 0.53 | 158 | 157 | 6.00E-47 | N(G),N(G)-dimethylarginine dimethylaminohydrolase 1 OS=Bos taurus GN=DDAH1 PE=1 SV=3 |
| evm.model.scaffold_390.97 | 0.35 | 102 | 69.3 | 3.00E-14 | N(G),N(G)-dimethylarginine dimethylaminohydrolase 1 OS=Bos taurus GN=DDAH1 PE=1 SV=3 |
| evm.model.scaffold_40.1 | 0.28 | 255 | 82 | 2.00E-17 | Upstream activation factor subunit spp27 OS=Schizosaccharomyces pombe (strain 972 / ATCC 24843) GN=spp27 PE=1 SV=1 |
| evm.model.scaffold_400.1.2 | 0.61 | 162 | 176 | 2.00E-52 | Homeobox protein homothorax OS=Drosophila melanogaster GN=hth PE=1 SV=1 |
| evm.model.scaffold_400.137 | 0.43 | 829 | 683 | 0 | Probable ATP-dependent DNA helicase HFM1 OS=Xenopus tropicalis GN=hfm1 PE=2 SV=1 |
| evm.model.scaffold_400.138 | 0.37 | 178 | 126 | 5.00E-36 | Fas apoptotic inhibitory molecule 1 OS=Bos taurus GN=FAIM PE=2 SV=1 |
| evm.model.scaffold_400.149 | 0.22 | 541 | 86.3 | 5.00E-16 | Glutamate receptor ionotropic, kainate 2 OS=Macaca fascicularis GN=GRIK2 PE=2 SV=1 |
| evm.model.scaffold_400.150 | NA | NA | NA | NA | No blast hit |
| evm.model.scaffold_400.151 | 0.39 | 235 | 154 | 1.00E-44 | Chymotrypsin OS=Phaedon cochleariae PE=2 SV=1 |
| evm.model.scaffold_400.155 | 0.38 | 223 | 148 | 3.00E-34 | Zinc finger protein 40 OS=Homo sapiens GN=HIVEP1 PE=1 SV=3 |
| evm.model.scaffold_400.156 | 0.46 | 113 | 117 | 1.00E-33 | Transmembrane protein 50B OS=Pongo abelii GN=TMEM50B PE=2 SV=1 |
| evm.model.scaffold_400.164 | 0.77 | 99 | 168 | 4.00E-42 | PREDICTED: uncharacterized protein LOC664468 isoform X2 [Tribolium castaneum] |
| evm.model.scaffold_400.198 | 0.22 | 530 | 56.2 | 9.00E-07 | Neprilysin-1 OS=Caenorhabditis elegans GN=nep-1 PE=1 SV=3 |
| evm.model.scaffold_400.27 | 0.46 | 597 | 506 | 1.00E-169 | Dystrobrevin beta OS=Homo sapiens GN=DTNB PE=1 SV=1 |
| evm.model.scaffold_400.28 | 0.74 | 123 | 191 | 1.00E-51 | Protein EFR3 homolog cmp44E OS=Drosophila melanogaster GN=stmA PE=2 SV=3 |
| evm.model.scaffold_400.36 | 0.65 | 418 | 562 | 0 | hypothetical protein D910_04254 [Dendroctonus ponderosae] |
| evm.model.scaffold_400.37 | 0.55 | 65 | 58.2 | 3.00E-10 | DAZ-associated protein 2 OS=Rattus norvegicus GN=Dazap2 PE=3 SV=1 |
| evm.model.scaffold_400.42 | 0.54 | 486 | 546 | 0 | Synaptic vesicle 2-related protein OS=Homo sapiens GN=SVOP PE=2 SV=1 |
| evm.model.scaffold_400.51 | 0.92 | 48 | 94 | 5.00E-22 | hypothetical protein D910_07417 [Dendroctonus ponderosae] |
| evm.model.scaffold_400.53 | 0.75 | 108 | 177 | 2.00E-52 | 12 kDa FK506-binding protein OS=Drosophila melanogaster GN=FK506-bp2 PE=3 SV=2 |
| evm.model.scaffold_400.55 | 0.68 | 620 | 808 | 0 | Sodium/potassium/calcium exchanger Nckx30C OS=Drosophila melanogaster GN=Nckx30C PE=2 SV=4 |
| evm.model.scaffold_400.61 | 0.88 | 480 | 877 | 0 | hypothetical protein YQE_08753, partial [Dendroctonus ponderosae] |
| evm.model.scaffold_400.91 | 0.44 | 230 | 179 | 4.00E-50 | Heterogeneous nuclear ribonucleoprotein K OS=Gallus gallus GN=HNRNPK PE=2 SV=1 |
| evm.model.scaffold_400.98 | 0.28 | 329 | 127 | 1.00E-27 | Adhesion G-protein coupled receptor G4 OS=Mus musculus GN=Adgrg4 PE=3 SV=1 |
| evm.model.scaffold_415.1 | 0.28 | 242 | 94.4 | 4.00E-21 | Lachesin OS=Drosophila melanogaster GN=Lac PE=1 SV=2 |
| evm.model.scaffold_42.4 | 0.34 | 398 | 244 | 5.00E-72 | Venom serine protease 34 OS=Apis mellifera PE=2 SV=1 |
| evm.model.scaffold_422.3 | 0.91 | 789 | 1523 | 0 | hypothetical protein D910_02838 [Dendroctonus ponderosae] |
| evm.model.scaffold_422.6 | 0.58 | 637 | 769 | 0 | Nuclear protein localization protein 4 homolog OS=Drosophila melanogaster GN=Npl4 PE=1 SV=3 |
| evm.model.scaffold_427.18.1 | 0.35 | 120 | 76.6 | 1.00E-13 | PREDICTED: myb/SANT-like DNA-binding domain-containing protein 3 [Acyrthosiphon pisum] |
| evm.model.scaffold_427.23 | 0.36 | 88 | 53.9 | 3.00E-06 | Unconventional myosin-XVI OS=Mus musculus GN=Myo16 PE=1 SV=2 |
| evm.model.scaffold_442.1 | 0.63 | 193 | 261 | 2.00E-81 | Amidophosphoribosyltransferase OS=Drosophila melanogaster GN=Prat PE=1 SV=2 |
| evm.model.scaffold_442.9_evm.model.scaffold_442.13 | 0.31 | 518 | 239 | 3.00E-71 | Putative tRNA pseudouridine synthase Pus10 OS=Mus musculus GN=Pus10 PE=2 SV=1 |
| evm.model.scaffold_447.10 | 0.56 | 350 | 409 | 4.00E-136 | PREDICTED: uncharacterized protein LOC100141786 isoform X1 [Tribolium castaneum] |
| evm.model.scaffold_447.62 | 0.70 | 135 | 198 | 7.00E-55 | hypothetical protein TcasGA2_TC008643 [Tribolium castaneum] |
| evm.model.scaffold_447.63 | 0.28 | 202 | 62.4 | 2.00E-09 | Nephrin OS=Homo sapiens GN=NPHS1 PE=1 SV=1 |
| evm.model.scaffold_451.32 | 0.64 | 154 | 195 | 8.00E-64 | Mediator of RNA polymerase II transcription subunit 21 OS=Aedes aegypti GN=MED21 PE=3 SV=1 |
| evm.model.scaffold_451.60 | 0.47 | 994 | 870 | 0 | Ephrin type-A receptor 4-A OS=Xenopus laevis GN=epha4-a PE=2 SV=1 |
| evm.model.scaffold_455.15 | 0.93 | 195 | 294 | 1.00E-97 | hypothetical protein YQE_07557, partial [Dendroctonus ponderosae] |
| evm.model.scaffold_455.7 | 0.47 | 1676 | 1364 | 0 | Mediator of RNA polymerase II transcription subunit 12 OS=Drosophila melanogaster GN=kto PE=1 SV=2 |
| evm.model.scaffold_463.10 | 0.61 | 186 | 207 | 6.00E-63 | Gamma-aminobutyric acid receptor subunit beta OS=Drosophila simulans GN=Rdl PE=2 SV=1 |
| evm.model.scaffold_463.170 | NA | NA | NA | NA | No blast hit |
| evm.model.scaffold_463.245 | 0.35 | 589 | 361 | 1.00E-111 | Exonuclease mut-7 homolog OS=Aedes aegypti GN=AAEL005527 PE=3 SV=1 |
| evm.model.scaffold_463.267 | 0.37 | 99 | 70.5 | 1.00E-15 | GSK3-beta interaction protein OS=Macaca fascicularis GN=GSKIP PE=2 SV=1 |
| evm.model.scaffold_463.268 | 0.42 | 461 | 296 | 1.00E-90 | Transmembrane and coiled-coil domains protein 1 OS=Homo sapiens GN=TMCC1 PE=1 SV=3 |
| evm.model.scaffold_471.144 | 0.39 | 514 | 382 | 1.00E-117 | Cytochrome P450 6A1 OS=Musca domestica GN=CYP6A1 PE=2 SV=1 |
| evm.model.scaffold_471.164 | 0.58 | 202 | 239 | 2.00E-78 | Endoglucanase OS=Phaedon cochleariae PE=2 SV=1 |
| evm.model.scaffold_471.165 | 0.49 | 223 | 235 | 3.00E-75 | Putative endoglucanase type K OS=Fusarium oxysporum PE=2 SV=1 |
| evm.model.scaffold_471.37 | 0.52 | 1232 | 1102 | 0 | Liprin-alpha-2 OS=Homo sapiens GN=PPFIA2 PE=1 SV=2 |
| evm.model.scaffold_474.17 | 0.66 | 256 | 381 | 1.00E-130 | Heparan sulfate glucosamine 3-O-sulfotransferase 3A1 OS=Mus musculus GN=Hs3st3a1 PE=2 SV=1 |
| evm.model.scaffold_486.8 | 0.48 | 245 | 241 | 3.00E-75 | Trypsin-4 OS=Anopheles gambiae GN=TRYP4 PE=2 SV=2 |
| evm.model.scaffold_487.58 | 0.55 | 293 | 341 | 1.00E-112 | Mitochondrial uncoupling protein 4 OS=Homo sapiens GN=SLC25A27 PE=2 SV=1 |
| evm.model.scaffold_50.28 | 0.43 | 286 | 213 | 2.00E-62 | Paired box protein Pax-6 OS=Coturnix coturnix japonica GN=PAX6 PE=2 SV=1 |
| evm.model.scaffold_50.36 | 0.57 | 136 | 143 | 2.00E-39 | ATP-binding cassette sub-family G member 4 OS=Homo sapiens GN=ABCG4 PE=1 SV=2 |
| evm.model.scaffold_502.19 | 0.67 | 483 | 622 | 0 | Vesicular acetylcholine transporter OS=Drosophila melanogaster GN=VAChT PE=2 SV=2 |
| evm.model.scaffold_502.25 | NA | NA | NA | NA | No blast hit |
| evm.model.scaffold_502.57 | 0.51 | 729 | 634 | 0 | Nuclear hormone receptor FTZ-F1 beta OS=Drosophila melanogaster GN=Hr39 PE=1 SV=3 |
| evm.model.scaffold_51.1 | 0.56 | 326 | 374 | 1.00E-125 | T-box transcription factor TBX20 OS=Gallus gallus GN=TBX20 PE=2 SV=1 |
| evm.model.scaffold_515.7_evm.model.scaffold_515.8 | 0.56 | 136 | 181 | 6.00E-50 | Arf-GAP domain and FG repeat-containing protein 1 OS=Mus musculus GN=Agfg1 PE=1 SV=2 |
| evm.model.scaffold_518.2 | 0.4 | 1303 | 840 | 0 | Multidrug resistance-associated protein 7 OS=Homo sapiens GN=ABCC10 PE=1 SV=1 |
| evm.model.scaffold_52.10.3 | 0.41 | 215 | 160 | 1.00E-41 | Zinc finger protein 362 OS=Homo sapiens GN=ZNF362 PE=1 SV=1 |
| evm.model.scaffold_524.13 | 0.39 | 353 | 244 | 4.00E-74 | Mannosyl-oligosaccharide alpha-1,2-mannosidase isoform A OS=Drosophila melanogaster GN=alpha-Man-I PE=1 SV=2 |
| evm.model.scaffold_524.6 | 0.4 | 873 | 631 | 0 | Glutamyl aminopeptidase OS=Homo sapiens GN=ENPEP PE=1 SV=3 |
| evm.model.scaffold_528.22 | 0.48 | 330 | 297 | 1.00E-98 | Nuclear migration protein nudC OS=Gallus gallus GN=NUDC PE=2 SV=1 |
| evm.model.scaffold_53.183 | 0.51 | 604 | 605 | 0 | SUMO-activating enzyme subunit 2 OS=Homo sapiens GN=UBA2 PE=1 SV=2 |
| evm.model.scaffold_535.25 | 0.93 | 97 | 183 | 3.00E-57 | hypothetical protein D910_09071 [Dendroctonus ponderosae] |
| evm.model.scaffold_537.9 | 0.54 | 188 | 238 | 1.00E-63 | EH domain-binding protein 1 OS=Mus musculus GN=Ehbp1 PE=1 SV=3 |
| evm.model.scaffold_54.101 | 0.36 | 230 | 125 | 1.00E-30 | GTP-binding protein REM 1 OS=Mus musculus GN=Rem1 PE=1 SV=1 |
| evm.model.scaffold_54.102 | 0.6 | 792 | 974 | 0 | Probable cleavage and polyadenylation specificity factor subunit 2 OS=Drosophila melanogaster GN=Cpsf100 PE=1 SV=1 |
| evm.model.scaffold_544.19.3 | 0.28 | 266 | 84.3 | 6.00E-15 | Cordon-bleu protein-like 1 OS=Mus musculus GN=Cobll1 PE=1 SV=2 |
| evm.model.scaffold_55.214 | 0.59 | 569 | 671 | 0 | Putative polypeptide N-acetylgalactosaminyltransferase 9 OS=Drosophila melanogaster GN=pgant9 PE=2 SV=2 |
| evm.model.scaffold_554.4.2 | 0.3 | 842 | 312 | 9.00E-85 | Ankyrin-1 OS=Homo sapiens GN=ANK1 PE=1 SV=3 |
| evm.model.scaffold_554.63 | 0.73 | 53 | 98.2 | 4.00E-23 | Mushroom body large-type Kenyon cell-specific protein 1 OS=Apis mellifera GN=Mblk-1 PE=1 SV=1 |
| evm.model.scaffold_56.10 | 0.81 | 542 | 842 | 0 | hypothetical protein D910_00430, partial [Dendroctonus ponderosae] |
| evm.model.scaffold_56.11 | 0.5 | 205 | 221 | 2.00E-69 | Dexamethasone-induced Ras-related protein 1 OS=Homo sapiens GN=RASD1 PE=1 SV=1 |
| evm.model.scaffold_56.35 | 0.57 | 231 | 299 | 1.00E-101 | E3 ubiquitin-protein ligase NRDP1 OS=Xenopus laevis GN=rnf41 PE=2 SV=1 |
| evm.model.scaffold_561.101 | 0.36 | 765 | 335 | 1.00E-93 | PREDICTED: uncharacterized protein LOC103314558 [Tribolium castaneum] |
| evm.model.scaffold_561.18 | 0.24 | 361 | 87 | 1.00E-16 | Nucleolar protein 11 OS=Xenopus laevis GN=nol11 PE=2 SV=2 |
| evm.model.scaffold_561.24 | 0.42 | 596 | 478 | 1.00E-153 | Glucose dehydrogenase [FAD, quinone] OS=Drosophila pseudoobscura pseudoobscura GN=Gld PE=3 SV=4 |
| evm.model.scaffold_561.29 | 0.57 | 363 | 426 | 1.00E-128 | BMP-2-inducible protein kinase OS=Homo sapiens GN=BMP2K PE=1 SV=2 |
| evm.model.scaffold_561.3 | 0.5 | 402 | 376 | 7.00E-124 | PREDICTED: uncharacterized protein LOC100142468 [Tribolium castaneum] |
| evm.model.scaffold_561.52.7 | 0.23 | 892 | 111 | 4.00E-23 | Hemicentin-2 OS=Mus musculus GN=Hmcn2 PE=2 SV=1 |
| evm.model.scaffold_561.55 | 0.30 | 380 | 130 | 2.00E-27 | hypothetical protein TcasGA2_TC006984 [Tribolium castaneum] |
| evm.model.scaffold_561.67 | 0.5 | 497 | 521 | 1.00E-178 | Carnitine O-palmitoyltransferase 1, muscle isoform OS=Homo sapiens GN=CPT1B PE=1 SV=2 |
| evm.model.scaffold_561.85 | 0.36 | 216 | 122 | 4.00E-29 | Prostasin OS=Homo sapiens GN=PRSS8 PE=1 SV=1 |
| evm.model.scaffold_561.90 | 0.39 | 161 | 108 | 1.00E-28 | Heat shock protein 23 OS=Drosophila melanogaster GN=Hsp23 PE=2 SV=2 |
| evm.model.scaffold_566.2 | 0.4 | 187 | 134 | 9.00E-36 | Glutamate receptor ionotropic, kainate 1 OS=Rattus norvegicus GN=Grik1 PE=1 SV=3 |
| evm.model.scaffold_566.20 | 0.38 | 488 | 330 | 1.00E-107 | Sialin OS=Homo sapiens GN=SLC17A5 PE=1 SV=2 |
| evm.model.scaffold_57.3 | 0.46 | 135 | 142 | 6.00E-37 | Zinc finger protein 500 OS=Homo sapiens GN=ZNF500 PE=2 SV=2 |
| evm.model.scaffold_57.6 | 0.45 | 662 | 598 | 0 | DNA helicase MCM9 OS=Xenopus tropicalis GN=mcm9 PE=3 SV=1 |
| evm.model.scaffold_575.22 | 0.47 | 916 | 798 | 0 | Staphylococcal nuclease domain-containing protein 1 OS=Pongo abelii GN=SND1 PE=2 SV=1 |
| evm.model.scaffold_588.16 | 0.5 | 102 | 95.5 | 6.00E-25 | Cardioactive peptide OS=Manduca sexta GN=CCAP PE=1 SV=1 |
| evm.model.scaffold_59.188 | 0.38 | 231 | 154 | 2.00E-38 | Harmonin OS=Homo sapiens GN=USH1C PE=1 SV=3 |
| evm.model.scaffold_59.224 | 0.28 | 288 | 115 | 8.00E-29 | Uncharacterized protein C15orf26 homolog OS=Mus musculus PE=2 SV=1 |
| evm.model.scaffold_59.241.1 | 0.56 | 219 | 250 | 4.00E-79 | T-box transcription factor TBX20 OS=Xenopus tropicalis GN=tbx20 PE=2 SV=1 |
| evm.model.scaffold_59.50 | 0.80 | 185 | 305 | 4.00E-102 | hypothetical protein D910_09243 [Dendroctonus ponderosae] |
| evm.model.scaffold_59.60 | 0.27 | 355 | 87 | 6.00E-17 | Neural cell adhesion molecule 1 OS=Homo sapiens GN=NCAM1 PE=1 SV=3 |
| evm.model.scaffold_604.10 | 0.36 | 980 | 508 | 1.00E-162 | Protein phosphatase PHLPP-like protein OS=Drosophila melanogaster GN=Phlpp PE=3 SV=1 |
| evm.model.scaffold_604.12 | 0.36 | 274 | 171 | 2.00E-51 | Cysteine-rich secretory protein 2 OS=Homo sapiens GN=CRISP2 PE=1 SV=1 |
| evm.model.scaffold_604.41_evm.model.scaffold_604.42 | 0.26 | 188 | 50.4 | 1.00E-05 | Nectin-4 OS=Mus musculus GN=Pvrl4 PE=2 SV=1 |
| evm.model.scaffold_608.71 | 0.38 | 256 | 179 | 3.00E-51 | Octopamine receptor OS=Bombyx mori PE=2 SV=1 |
| evm.model.scaffold_608.88 | 0.6 | 818 | 949 | 0 | Ankyrin repeat and KH domain-containing protein 1 OS=Homo sapiens GN=ANKHD1 PE=1 SV=1 |
| evm.model.scaffold_609.1 | 0.25 | 261 | 75.9 | 9.00E-13 | Nephrin OS=Rattus norvegicus GN=Nphs1 PE=1 SV=2 |
| evm.model.scaffold_62.115 | 0.99 | 233 | 369 | 1.00E-121 | hypothetical protein D910_03944 [Dendroctonus ponderosae] |
| evm.model.scaffold_62.116 | 0.45 | 83 | 53.9 | 2.00E-06 | rrbp1; ribosome binding protein 1; K14000 ribosome-binding protein 1 (A) |
| evm.model.scaffold_62.6.2 | 0.53 | 116 | 144 | 5.00E-37 | Modifier of mdg4 OS=Drosophila melanogaster GN=mod(mdg4) PE=1 SV=1 |
| evm.model.scaffold_625.2 | 0.67 | 67 | 111 | 4.00E-28 | GJ23623 gene product from transcript GJ23623-RB; K18080 tensin (A) |
| evm.model.scaffold_63.11 | 0.25 | 225 | 71.2 | 1.00E-10 | 63 kDa sperm flagellar membrane protein OS=Strongylocentrotus purpuratus PE=2 SV=1 |
| evm.model.scaffold_631.2 | 0.74 | 1063 | 1385 | 0 | hypothetical protein YQE_05794, partial [Dendroctonus ponderosae] |
| evm.model.scaffold_633.34 | 0.43 | 53 | 50.8 | 5.00E-06 | Broad-complex core protein isoform 6 OS=Drosophila melanogaster GN=br PE=1 SV=2 |
| evm.model.scaffold_633.6 | 0.67 | 110 | 149 | 1.00E-41 | Voltage-dependent T-type calcium channel subunit alpha-1I OS=Homo sapiens GN=CACNA1I PE=1 SV=1 |
| evm.model.scaffold_633.93 | 0.48 | 612 | 543 | 0 | Polypeptide N-acetylgalactosaminyltransferase 3 OS=Drosophila melanogaster GN=pgant3 PE=1 SV=1 |
| evm.model.scaffold_639.2 | 0.35 | 194 | 126 | 2.00E-31 | U3 small nucleolar RNA-associated protein 14 homolog A OS=Bos taurus GN=UTP14A PE=2 SV=1 |
| evm.model.scaffold_64.87 | 0.48 | 84 | 72 | 4.00E-17 | Tenecin-1 OS=Tenebrio molitor PE=1 SV=1 |
| evm.model.scaffold_64.98 | 0.38 | 1083 | 696 | 0 | Symplekin OS=Drosophila melanogaster GN=Sym PE=1 SV=1 |
| evm.model.scaffold_662.8 | 0.31 | 447 | 239 | 4.00E-67 | Frizzled-10-A OS=Xenopus laevis GN=fzd10-a PE=2 SV=1 |
| evm.model.scaffold_668.1 | 0.24 | 280 | 56.6 | 8.00E-07 | Neuropilin-2 OS=Rattus norvegicus GN=Nrp2 PE=2 SV=1 |
| evm.model.scaffold_668.11 | 0.48 | 76 | 61.6 | 2.00E-09 | Ring canal kelch homolog OS=Anopheles stephensi GN=kel PE=2 SV=2 |
| evm.model.scaffold_674.3 | 0.97 | 75 | 161 | 1.00E-48 | Tubulin alpha-3 chain OS=Bos taurus GN=TUBA3 PE=2 SV=1 |
| evm.model.scaffold_675.3 | 0.39 | 176 | 139 | 3.00E-33 | Probable ATP-dependent RNA helicase DDX20 OS=Danio rerio GN=ddx20 PE=3 SV=1 |
| evm.model.scaffold_7.39 | 0.87 | 217 | 419 | 7.00E-139 | hypothetical protein D910_12371, partial [Dendroctonus ponderosae] |
| evm.model.scaffold_70.1.2 | 0.4 | 355 | 225 | 2.00E-59 | Angiomotin OS=Homo sapiens GN=AMOT PE=1 SV=1 |
| evm.model.scaffold_703.1 | 0.92 | 100 | 180 | 1.00E-51 | hypothetical protein YQE_10783, partial [Dendroctonus ponderosae] |
| evm.model.scaffold_703.15 | 0.31 | 133 | 62.8 | 2.00E-09 | Trans-Golgi network integral membrane protein 2 OS=Homo sapiens GN=TGOLN2 PE=1 SV=2 |
| evm.model.scaffold_703.16 | 0.27 | 102 | 51.6 | 1.00E-07 | CAAX prenyl protease 1 homolog OS=Arabidopsis thaliana GN=FACE1 PE=1 SV=1 |
| evm.model.scaffold_703.2 | 0.47 | 201 | 186 | 1.00E-58 | 39S ribosomal protein L32, mitochondrial OS=Drosophila melanogaster GN=mRpL32 PE=2 SV=1 |
| evm.model.scaffold_703.3 | 0.55 | 329 | 375 | 1.00E-126 | Decaprenyl-diphosphate synthase subunit 1 OS=Homo sapiens GN=PDSS1 PE=1 SV=1 |
| evm.model.scaffold_728.3 | 0.58 | 368 | 450 | 1.00E-149 | Gelsolin OS=Drosophila melanogaster GN=Gel PE=1 SV=2 |
| evm.model.scaffold_73.9 | 0.4 | 375 | 257 | 2.00E-80 | Cholecystokinin receptor OS=Xenopus laevis GN=cckar PE=2 SV=1 |
| evm.model.scaffold_732.10 | 0.90 | 151 | 269 | 4.00E-89 | hypothetical protein YQE_03022, partial [Dendroctonus ponderosae] |
| evm.model.scaffold_732.11 | 0.49 | 672 | 644 | 0 | UPF0668 protein C10orf76 OS=Homo sapiens GN=C10orf76 PE=2 SV=1 |
| evm.model.scaffold_732.16 | 0.42 | 760 | 614 | 0 | Chorion peroxidase OS=Drosophila melanogaster GN=Pxt PE=2 SV=3 |
| evm.model.scaffold_732.8 | 0.35 | 444 | 276 | 4.00E-74 | GTPase-activating protein and VPS9 domain-containing protein 1 OS=Mus musculus GN=Gapvd1 PE=1 SV=2 |
| evm.model.scaffold_732.9 | 0.65 | 354 | 516 | 0 | 2-oxoisovalerate dehydrogenase subunit beta, mitochondrial OS=Bos taurus GN=BCKDHB PE=1 SV=2 |
| evm.model.scaffold_748.1_evm.model.scaffold_748.2 | 0.55 | 247 | 272 | 3.00E-89 | BTB/POZ domain-containing protein KCTD15 OS=Mus musculus GN=Kctd15 PE=1 SV=1 |
| evm.model.scaffold_749.4 | 0.35 | 903 | 525 | 1.00E-163 | Roundabout homolog 2 OS=Homo sapiens GN=ROBO2 PE=1 SV=2 |
| evm.model.scaffold_749.43 | 0.57 | 98 | 112 | 4.00E-30 | Graves disease carrier protein OS=Bos taurus GN=SLC25A16 PE=2 SV=1 |
| evm.model.scaffold_749.54 | 0.34 | 197 | 110 | 1.00E-24 | GD10796 gene product from transcript GD10796-RA; K11426 SET and MYND domain-containing protein (A) |
| evm.model.scaffold_749.6 | 0.28 | 262 | 107 | 4.00E-26 | E3 ubiquitin-protein ligase Siah2 OS=Danio rerio GN=siah2l PE=2 SV=2 |
| evm.model.scaffold_75.10 | 0.31 | 111 | 59.7 | 2.00E-10 | Alcohol dehydrogenase 2 OS=Ceratitis capitata GN=ADH2 PE=3 SV=1 |
| evm.model.scaffold_752.1 | 0.38 | 393 | 258 | 7.00E-81 | Orexin receptor type 2 OS=Mus musculus GN=Hcrtr2 PE=2 SV=2 |
| evm.model.scaffold_752.19 | 0.85 | 55 | 108 | 3.00E-28 | Junctophilin-1 OS=Mus musculus GN=Jph1 PE=2 SV=1 |
| evm.model.scaffold_752.30.1 | 0.6 | 207 | 257 | 3.00E-86 | Protein big brother OS=Drosophila melanogaster GN=Bgb PE=2 SV=3 |
| evm.model.scaffold_759.1 | 0.44 | 134 | 117 | 3.00E-31 | Spermine synthase OS=Bos taurus GN=SMS PE=2 SV=1 |
| evm.model.scaffold_770.1 | 0.26 | 201 | 63.5 | 2.00E-10 | Kynurenine formamidase OS=Bacillus cereus subsp. cytotoxis (strain NVH 391-98) GN=kynB PE=3 SV=1 |
| evm.model.scaffold_771.60 | 0.32 | 492 | 204 | 6.00E-59 | Ubiquitin carboxyl-terminal hydrolase 30 OS=Danio rerio GN=usp30 PE=3 SV=1 |
| evm.model.scaffold_773.48 | NA | NA | NA | NA | No blast hit |
| evm.model.scaffold_776.151.3 | 0.48 | 461 | 404 | 1.00E-135 | DNA-binding protein P3A2 OS=Strongylocentrotus purpuratus PE=1 SV=1 |
| evm.model.scaffold_776.184 | 0.28 | 280 | 135 | 7.00E-35 | Lachesin OS=Schistocerca americana GN=LAC PE=1 SV=1 |
| evm.model.scaffold_776.214 | 0.28 | 189 | 57.8 | 4.00E-08 | Neuronal growth regulator 1 OS=Mus musculus GN=Negr1 PE=1 SV=1 |
| evm.model.scaffold_776.234 | 0.34 | 408 | 166 | 5.00E-40 | Uncharacterized protein CG43427 OS=Drosophila melanogaster GN=CG43427 PE=2 SV=1 |
| evm.model.scaffold_776.235 | 0.59 | 435 | 516 | 0 | Protein disulfide-isomerase A6 OS=Rattus norvegicus GN=Pdia6 PE=1 SV=2 |
| evm.model.scaffold_776.272 | 0.28 | 154 | 54.3 | 6.00E-07 | Ectodysplasin-A OS=Bos taurus GN=EDA PE=3 SV=2 |
| evm.model.scaffold_776.30 | 0.4 | 2504 | 1647 | 0 | E3 ubiquitin-protein ligase HUWE1 OS=Homo sapiens GN=HUWE1 PE=1 SV=3 |
| evm.model.scaffold_776.300 | 0.67 | 184 | 260 | 1.00E-88 | Translocon-associated protein subunit gamma OS=Homo sapiens GN=SSR3 PE=1 SV=1 |
| evm.model.scaffold_776.301 | 0.4 | 386 | 209 | 1.00E-61 | Transcriptional coactivator YAP1 OS=Rattus norvegicus GN=Yap1 PE=1 SV=1 |
| evm.model.scaffold_776.302 | 0.25 | 1081 | 291 | 1.00E-77 | Apolipophorins OS=Locusta migratoria PE=1 SV=2 |
| evm.model.scaffold_776.316 | 0.39 | 409 | 292 | 4.00E-94 | Battenin OS=Canis familiaris GN=CLN3 PE=3 SV=1 |
| evm.model.scaffold_776.322 | 0.41 | 448 | 355 | 1.00E-117 | Alpha-1,6-mannosyl-glycoprotein 2-beta-N-acetylglucosaminyltransferase OS=Rattus norvegicus GN=Mgat2 PE=1 SV=1 |
| evm.model.scaffold_776.324.2 | 0.32 | 117 | 51.2 | 2.00E-05 | Nuclear protein MDM1 OS=Rattus norvegicus GN=Mdm1 PE=2 SV=2 |
| evm.model.scaffold_776.326 | 0.58 | 592 | 707 | 0 | PREDICTED: uncharacterized protein LOC661132 [Tribolium castaneum] |
| evm.model.scaffold_776.44 | 0.45 | 84 | 80.1 | 4.00E-18 | Peflin OS=Homo sapiens GN=PEF1 PE=1 SV=1 |
| evm.model.scaffold_776.49 | 0.7 | 318 | 461 | 1.00E-163 | Electron transfer flavoprotein subunit alpha, mitochondrial OS=Macaca fascicularis GN=ETFA PE=2 SV=1 |
| evm.model.scaffold_776.83 | 0.76 | 210 | 345 | 1.00E-115 | Fez family zinc finger protein 2 OS=Mus musculus GN=Fezf2 PE=2 SV=1 |
| evm.model.scaffold_776.84 | 0.37 | 1090 | 701 | 0 | Guanine nucleotide exchange factor DBS OS=Homo sapiens GN=MCF2L PE=1 SV=2 |
| evm.model.scaffold_776.94 | 0.5 | 199 | 215 | 1.00E-55 | Zinc finger protein basonuclin-2 OS=Homo sapiens GN=BNC2 PE=1 SV=1 |
| evm.model.scaffold_78.34 | 0.39 | 79 | 61.6 | 2.00E-08 | CAP-Gly domain-containing linker protein 1 OS=Gallus gallus GN=CLIP1 PE=2 SV=1 |
| evm.model.scaffold_78.4 | 0.76 | 153 | 242 | 3.00E-76 | hypothetical protein TcasGA2_TC014772 [Tribolium castaneum] |
| evm.model.scaffold_78.48_evm.model.scaffold_78.49 | 0.54 | 519 | 561 | 0 | Importin subunit alpha-1 OS=Homo sapiens GN=KPNA2 PE=1 SV=1 |
| evm.model.scaffold_78.6 | 0.90 | 335 | 617 | 0 | hypothetical protein D910_02278 [Dendroctonus ponderosae] |
| evm.model.scaffold_78.7 | 0.36 | 100 | 52.4 | 5.00E-06 | PREDICTED: uncharacterized protein LOC105695640 [Orussus abietinus] |
| evm.model.scaffold_78.8 | 0.36 | 291 | 193 | 2.00E-48 | Connector enhancer of kinase suppressor of ras 2 OS=Mus musculus GN=Cnksr2 PE=1 SV=1 |
| evm.model.scaffold_781.3.1 | 0.45 | 371 | 297 | 4.00E-85 | Transcription factor hamlet OS=Drosophila melanogaster GN=ham PE=2 SV=1 |
| evm.model.scaffold_781.5.3 | 0.76 | 974 | 1312 | 0 | hypothetical protein YQE_05794, partial [Dendroctonus ponderosae] |
| evm.model.scaffold_781.8 | 0.33 | 679 | 363 | 1.00E-115 | ABC transporter G family member 14 OS=Arabidopsis thaliana GN=ABCG14 PE=2 SV=1 |
| evm.model.scaffold_788.7 | 0.22 | 490 | 66.6 | 2.00E-10 | Sodium channel protein Nach OS=Drosophila melanogaster GN=Nach PE=2 SV=2 |
| evm.model.scaffold_79.32 | 0.46 | 338 | 311 | 1.00E-102 | Peptide chain release factor 1-like, mitochondrial OS=Homo sapiens GN=MTRF1L PE=1 SV=1 |
| evm.model.scaffold_79.48 | 0.67 | 52 | 75.1 | 4.00E-15 | Decaprenyl-diphosphate synthase subunit 2 OS=Homo sapiens GN=PDSS2 PE=1 SV=2 |
| evm.model.scaffold_79.5 | 0.63 | 279 | 372 | 1.00E-130 | Proteasome subunit beta type-7 OS=Rattus norvegicus GN=Psmb7 PE=1 SV=1 |
| evm.model.scaffold_79.50 | 0.46 | 1134 | 935 | 0 | Afadin OS=Homo sapiens GN=MLLT4 PE=1 SV=3 |
| evm.model.scaffold_797.10 | 0.27 | 213 | 89 | 1.00E-17 | BTB/POZ domain-containing protein 2 OS=Homo sapiens GN=BTBD2 PE=1 SV=1 |
| evm.model.scaffold_797.12 | 0.4 | 1259 | 964 | 0 | Probable multidrug resistance-associated protein lethal(2)03659 OS=Drosophila melanogaster GN=l(2)03659 PE=2 SV=3 |
| evm.model.scaffold_797.14 | 0.67 | 204 | 294 | 1.00E-101 | Ras-related protein Rab-18-B OS=Danio rerio GN=rab18b PE=2 SV=1 |
| evm.model.scaffold_797.15 | NA | NA | NA | NA | No blast hit |
| evm.model.scaffold_797.16 | 0.34 | 136 | 58.5 | 5.00E-09 | Stathmin-2-B OS=Xenopus laevis GN=stmn2-b PE=2 SV=1 |
| evm.model.scaffold_797.2 | 0.33 | 500 | 117 | 3.00E-24 | PREDICTED: extensin-like isoform X1 [Musca domestica] |
| evm.model.scaffold_797.9 | 0.99 | 196 | 414 | 1.00E-144 | hypothetical protein YQE_08302, partial [Dendroctonus ponderosae] |
| evm.model.scaffold_80.1 | 0.32 | 295 | 154 | 9.00E-40 | Polycomb group RING finger protein 2 OS=Mus musculus GN=Pcgf2 PE=1 SV=2 |
| evm.model.scaffold_801.9 | 0.42 | 319 | 248 | 1.00E-79 | Cyclin-H OS=Mus musculus GN=Ccnh PE=2 SV=2 |
| evm.model.scaffold_813.1 | 0.31 | 276 | 145 | 2.00E-39 | Corticotropin-releasing factor-binding protein OS=Ovis aries GN=CRHBP PE=2 SV=1 |
| evm.model.scaffold_816.20 | 0.46 | 138 | 123 | 9.00E-30 | Lipase member H-A OS=Xenopus laevis GN=liph-a PE=2 SV=1 |
| evm.model.scaffold_818.43 | 0.66 | 1944 | 2424 | 0 | Chromodomain-helicase-DNA-binding protein Mi-2 homolog OS=Drosophila melanogaster GN=Mi-2 PE=1 SV=2 |
| evm.model.scaffold_82.12.3 | 0.74 | 834 | 1293 | 0 | Protein argonaute-2 OS=Mus musculus GN=Ago2 PE=1 SV=3 |
| evm.model.scaffold_821.2 | 0.44 | 247 | 233 | 5.00E-67 | Zinc finger protein 235 OS=Homo sapiens GN=ZNF235 PE=2 SV=3 |
| evm.model.scaffold_821.3 | NA | NA | NA | NA | No blast hit |
| evm.model.scaffold_822.10 | 0.48 | 2092 | 1907 | 0 | Phosphatidylinositol 4-kinase alpha OS=Homo sapiens GN=PI4KA PE=1 SV=3 |
| evm.model.scaffold_822.11 | 0.49 | 416 | 428 | 1.00E-145 | Protein RCC2 OS=Homo sapiens GN=RCC2 PE=1 SV=2 |
| evm.model.scaffold_832.4 | 0.96 | 90 | 180 | 7.00E-60 | U6 snRNA-associated Sm-like protein LSm2 OS=Mus musculus GN=Lsm2 PE=3 SV=1 |
| evm.model.scaffold_832.6 | NA | NA | NA | NA | No blast hit |
| evm.model.scaffold_832.7 | 0.55 | 149 | 160 | 1.00E-46 | LMX-HD, Lmx1b2; LIM homeobox transcription factor 1-alpha; K09371 LIM homeobox transcription factor 1 (A) |
| evm.model.scaffold_832.8 | 0.54 | 208 | 218 | 7.00E-69 | LIM homeobox transcription factor 1-alpha OS=Mus musculus GN=Lmx1a PE=2 SV=1 |
| evm.model.scaffold_832.9 | 0.65 | 755 | 957 | 0 | AFG3-like protein 2 OS=Mus musculus GN=Afg3l2 PE=1 SV=1 |
| evm.model.scaffold_842.34.1 | 0.59 | 447 | 560 | 0 | Phosphatidylinositol 4-phosphate 5-kinase type-1 alpha OS=Rattus norvegicus GN=Pip5k1a PE=2 SV=1 |
| evm.model.scaffold_842.82 | 0.6 | 352 | 417 | 1.00E-143 | Insulin gene enhancer protein ISL-1 OS=Rattus norvegicus GN=Isl1 PE=2 SV=1 |
| evm.model.scaffold_842.89 | 0.44 | 289 | 239 | 1.00E-76 | Transcription initiation factor TFIID subunit 8 OS=Drosophila melanogaster GN=Taf8 PE=1 SV=1 |
| evm.model.scaffold_85.310 | 0.36 | 925 | 525 | 1.00E-165 | Roundabout homolog 2 OS=Homo sapiens GN=ROBO2 PE=1 SV=2 |
| evm.model.scaffold_86.8 | 0.82 | 128 | 227 | 2.00E-71 | hypothetical protein YQE_09731, partial [Dendroctonus ponderosae] |
| evm.model.scaffold_88.14 | 0.28 | 298 | 96.7 | 2.00E-21 | Phosphoglycolate phosphatase 1B, chloroplastic OS=Arabidopsis thaliana GN=PGLP1B PE=1 SV=1 |
| evm.model.scaffold_88.15 | 0.4 | 62 | 57.8 | 1.00E-07 | PDZ and LIM domain protein Zasp OS=Drosophila melanogaster GN=Zasp52 PE=1 SV=2 |
| evm.model.scaffold_89.1 | 0.31 | 546 | 265 | 7.00E-80 | UPF0704 protein C6orf165 homolog OS=Bos taurus PE=2 SV=2 |
| evm.model.scaffold_89.34.3 | 0.89 | 738 | 1271 | 0 | hypothetical protein YQE_03158, partial [Dendroctonus ponderosae] |
| evm.model.scaffold_89.53 | 0.54 | 591 | 578 | 0 | Protein melted OS=Drosophila melanogaster GN=melt PE=1 SV=2 |
| evm.model.scaffold_89.54 | 0.8 | 171 | 290 | 1.00E-100 | Peptidyl-prolyl cis-trans isomerase H OS=Homo sapiens GN=PPIH PE=1 SV=1 |
| evm.model.scaffold_89.57 | 0.24 | 385 | 118 | 2.00E-28 | Potassium channel subfamily K member 18 OS=Rattus norvegicus GN=Kcnk18 PE=2 SV=1 |
| evm.model.scaffold_89.7.1 | 0.49 | 625 | 602 | 0 | Myotubularin-related protein 3 OS=Rattus norvegicus GN=Mtmr3 PE=2 SV=1 |
| evm.model.scaffold_89.8.2 | 0.34 | 1005 | 503 | 1.00E-159 | Protein diaphanous homolog 3 OS=Mus musculus GN=Diaph3 PE=1 SV=1 |
| evm.model.scaffold_890.207 | 0.24 | 583 | 142 | 2.00E-33 | WD repeat-containing protein 63 OS=Homo sapiens GN=WDR63 PE=2 SV=1 |
| evm.model.scaffold_892.3 | 0.33 | 328 | 174 | 2.00E-50 | Sulfotransferase 1C4 OS=Homo sapiens GN=SULT1C4 PE=1 SV=2 |
| evm.model.scaffold_898.26_evm.model.scaffold_898.27 | 0.49 | 459 | 422 | 1.00E-139 | E3 SUMO-protein ligase PIAS1 OS=Homo sapiens GN=PIAS1 PE=1 SV=2 |
| evm.model.scaffold_898.36 | 0.39 | 168 | 97.8 | 7.00E-25 | Uncharacterized protein CG16817 OS=Drosophila melanogaster GN=CG16817 PE=1 SV=1 |
| evm.model.scaffold_9.23.1 | 0.38 | 283 | 172 | 5.00E-52 | Protein twisted gastrulation OS=Drosophila melanogaster GN=tsg PE=1 SV=1 |
| evm.model.scaffold_9.28 | 0.29 | 677 | 301 | 1.00E-82 | Sushi, von Willebrand factor type A, EGF and pentraxin domain-containing protein 1 OS=Mus musculus GN=Svep1 PE=1 SV=1 |
| evm.model.scaffold_9.84 | 0.93 | 60 | 119 | 3.00E-28 | hypothetical protein D910_08800 [Dendroctonus ponderosae] |
| evm.model.scaffold_9.85 | 0.44 | 167 | 165 | 6.00E-49 | Zinc carboxypeptidase (Fragment) OS=Simulium vittatum PE=2 SV=1 |
| evm.model.scaffold_90.39 | 0.87 | 680 | 1126 | 0 | hypothetical protein YQE_08798, partial [Dendroctonus ponderosae] |
| evm.model.scaffold_913.6 | 0.7 | 747 | 1114 | 0 | Transient receptor potential cation channel subfamily A member 1 OS=Drosophila melanogaster GN=TrpA1 PE=2 SV=4 |
| evm.model.scaffold_93.21 | 0.45 | 286 | 239 | 4.00E-70 | Trafficking protein particle complex subunit 10 OS=Homo sapiens GN=TRAPPC10 PE=1 SV=2 |
| evm.model.scaffold_93.22 | 0.48 | 192 | 207 | 2.00E-60 | Trafficking protein particle complex subunit 10 OS=Drosophila melanogaster GN=SIDL PE=1 SV=1 |
| evm.model.scaffold_94.205 | 0.41 | 246 | 174 | 3.00E-48 | Transmembrane protease serine 7 OS=Mus musculus GN=Tmprss7 PE=1 SV=3 |
| evm.model.scaffold_95.118 | 0.36 | 176 | 104 | 5.00E-23 | Crossover junction endonuclease MUS81 OS=Danio rerio GN=mus81 PE=1 SV=1 |
| evm.model.scaffold_95.119 | 0.4 | 273 | 204 | 2.00E-63 | Pyrroline-5-carboxylate reductase OS=Actinidia chinensis PE=2 SV=1 |
| evm.model.scaffold_95.129 | 0.39 | 83 | 70.1 | 1.00E-10 | Hemicentin-1 OS=Homo sapiens GN=HMCN1 PE=1 SV=2 |
| evm.model.scaffold_95.60 | 0.34 | 389 | 225 | 7.00E-69 | Sister chromatid cohesion protein DCC1 OS=Xenopus tropicalis GN=dscc1 PE=2 SV=1 |
| evm.model.scaffold_95.61 | 0.43 | 1142 | 812 | 0 | Probable Rho GTPase-activating protein CG5521 OS=Drosophila melanogaster GN=CG5521 PE=1 SV=2 |
| evm.model.scaffold_96.109_evm.model.scaffold_96.108 | 0.39 | 273 | 199 | 2.00E-60 | Delta-sarcoglycan OS=Homo sapiens GN=SGCD PE=1 SV=2 |
| evm.model.scaffold_96.15 | 0.62 | 325 | 443 | 1.00E-153 | Sensory neuron membrane protein 1 OS=Tribolium castaneum GN=SNMP01 PE=3 SV=1 |
| evm.model.scaffold_97.21 | 0.35 | 190 | 129 | 2.00E-31 | Testican-2 OS=Homo sapiens GN=SPOCK2 PE=1 SV=1 |
| evm.model.scaffold_97.35.1 | 0.56 | 931 | 936 | 0 | Centaurin-gamma-1A OS=Drosophila melanogaster GN=CenG1A PE=2 SV=2 |
| evm.model.scaffold_97.43 | 0.25 | 327 | 99 | 1.00E-21 | Carbohydrate sulfotransferase 5 OS=Mus musculus GN=Chst5 PE=2 SV=1 |
| evm.model.scaffold_97.46 | 0.45 | 447 | 419 | 1.00E-142 | Diacylglycerol O-acyltransferase 1 OS=Mus musculus GN=Dgat1 PE=1 SV=1 |
| evm.model.scaffold_99.137 | 0.46 | 247 | 182 | 6.00E-51 | Zinc finger protein Elbow OS=Drosophila melanogaster GN=elB PE=1 SV=4 |
| evm.model.scaffold_99.149 | 0.33 | 401 | 214 | 2.00E-56 | Protein SMG5 OS=Mus musculus GN=Smg5 PE=2 SV=2 |
| evm.model.scaffold_99.3 | 0.56 | 391 | 461 | 1.00E-158 | CCR4-NOT transcription complex subunit 6 OS=Xenopus laevis GN=cnot6 PE=1 SV=1 |
