# Supplementary material for: Chromosome-level genome assembly and population genomic analyses provide insights into adaptive evolution of the red turpentine beetle, Dendroctonus valens
Source: BMC Biol. 2022 Aug 24;20:190. doi: 10.1186/s12915-022-01388-y (PMC9400205; doi:10.1186/s12915-022-01388-y)
Supplement: Supplementary file 1 — Additional file 1: Table S1. Summary statistics of genome sequencing data of Dendroctonus valens. Table S2. Summary statistics of genome assembly of Dendroctonus valens. Table S3. BUSCO evaluation result for genome assembly of Dendroctonus valens. Table S4. Summary statistics of transposable elements in Dendroctonus valens genome. Table S5. Summary of gene families manually curated in Dendroctonus valens genome. Table S6. Summary statistics of genome annotation in Dendroctonus valens genome. Table S7. List of gene families that are unique in Dendroctonus valens compared to other three Coleoptera species. Table S8. Gene families that are rapidly expanded in Dendroctonus valens revealed by CAFE analysis. Table S9. Gene families that are rapidly contracted in Dendroctonus valens revealed by CAFE analysis. Table S10. List of genes that are positively selected in Dendroctonus valens revealed by codeml analysis. Table S11. Gene ontology enrichment result of positively selected genes in Dendroctonus valens. Table S12. Sampling site information for genome resequencing of geographical populations. Table S13. Summary statistics of genome resequencing data in different populations. Table S14. List of genes that undergo selective sweep in the China population compared to CAMT population. [file 12915_2022_1388_MOESM1_ESM.zip › Table S2.docx]

| **Table S2** Summary statistics of genome assembly of *Dendroctonus valens* | | | | |
| --- | --- | --- | --- | --- |
| **Item** | **Contig number** | **Contig length** | **Scaffold number** | **Scaffold length** |
| Total | 1,144 | 320,964,741 | 923 | 322,406,701 |
| Max | - | 9,688,036 | - | 9,688,036 |
| >2 kb | 1,121 | - | 900 | - |
| N50 | 83 | 985,469 | 57 | 1,658,008 |
| N60 | 118 | 833,887 | 79 | 1,307,813 |
| N70 | 165 | 573,053 | 107 | 1,013,080 |
| N80 | 228 | 424,338 | 143 | 734,843 |
| N90 | 330 | 217,022 | 201 | 385,131 |
