# Supplementary material for: Chromosome-level genome assembly and population genomic analyses provide insights into adaptive evolution of the red turpentine beetle, Dendroctonus valens
Source: BMC Biol. 2022 Aug 24;20:190. doi: 10.1186/s12915-022-01388-y (PMC9400205; doi:10.1186/s12915-022-01388-y)
Supplement: Supplementary file 1 — Additional file 1: Table S1. Summary statistics of genome sequencing data of Dendroctonus valens. Table S2. Summary statistics of genome assembly of Dendroctonus valens. Table S3. BUSCO evaluation result for genome assembly of Dendroctonus valens. Table S4. Summary statistics of transposable elements in Dendroctonus valens genome. Table S5. Summary of gene families manually curated in Dendroctonus valens genome. Table S6. Summary statistics of genome annotation in Dendroctonus valens genome. Table S7. List of gene families that are unique in Dendroctonus valens compared to other three Coleoptera species. Table S8. Gene families that are rapidly expanded in Dendroctonus valens revealed by CAFE analysis. Table S9. Gene families that are rapidly contracted in Dendroctonus valens revealed by CAFE analysis. Table S10. List of genes that are positively selected in Dendroctonus valens revealed by codeml analysis. Table S11. Gene ontology enrichment result of positively selected genes in Dendroctonus valens. Table S12. Sampling site information for genome resequencing of geographical populations. Table S13. Summary statistics of genome resequencing data in different populations. Table S14. List of genes that undergo selective sweep in the China population compared to CAMT population. [file 12915_2022_1388_MOESM1_ESM.zip › Table S7.docx]

| **Table** **S7** List of gene families that are unique in *Dendroctonus valens* compared to other three Coleoptera species | | | | | | |
| --- | --- | --- | --- | --- | --- | --- |
| **TreeFam ID** | **Gene ID** | **Identity** | **Aligned length** | **Score** | **E-value** | **Description** |
| TF105233 | evm.model.scaffold_844.8 | 0.31 | 79 | 53.5 | 2.00E-08 | Kinesin-like protein KIF22 OS=Danio rerio GN=kif22 PE=2 SV=1 |
| TF106265 | evm.model.scaffold_836.1 | 0.44 | 127 | 106 | 5.00E-26 | Transformer-2 protein homolog beta OS=Rattus norvegicus GN=Tra2b PE=1 SV=1 |
| TF300294 | evm.model.scaffold_260.8 | 0.3 | 105 | 45.8 | 6.00E-06 | Proteasome assembly chaperone 3 OS=Mus musculus GN=Psmg3 PE=1 SV=1 |
| TF313141 | evm.model.scaffold_28.14 | 0.55 | 60 | 72.8 | 1.00E-17 | Protein BOLA2 OS=Arabidopsis thaliana GN=BOLA2 PE=1 SV=1 |
| TF313141 | evm.model.scaffold_71.49 | 0.55 | 60 | 72.8 | 1.00E-17 | Protein BOLA2 OS=Arabidopsis thaliana GN=BOLA2 PE=1 SV=1 |
| TF313408 | evm.model.scaffold_172.1 | 0.4 | 187 | 137 | 5.00E-37 | JmjC domain-containing protein 8 OS=Rattus norvegicus GN=Jmjd8 PE=2 SV=2 |
| TF313933 | evm.model.scaffold_842.66 | 0.44 | 96 | 90.5 | 7.00E-23 | Cysteine-rich DPF motif domain-containing protein 1 OS=Gallus gallus GN=CDPF1 PE=2 SV=1 |
| TF315885 | evm.model.scaffold_10.62 | 0.32 | 61 | 49.3 | 7.00E-06 | Insulinoma-associated protein 1b OS=Danio rerio GN=insm1b PE=2 SV=1 |
| TF316006 | evm.model.scaffold_774.13 | 0.54 | 177 | 172 | 4.00E-43 | Putative protein tag-278 OS=Caenorhabditis elegans GN=tag-278 PE=4 SV=1 |
| TF316733 | evm.model.scaffold_447.46.1 | 0.49 | 291 | 252 | 2.00E-73 | hypothetical protein TcasGA2_TC004274 [Tribolium castaneum] |
| TF318038 | evm.model.scaffold_94.133 | 0.28 | 74 | 47 | 8.00E-06 | Galactoside 2-alpha-L-fucosyltransferase 3 OS=Mus musculus GN=Sec1 PE=2 SV=1 |
| TF318600 | evm.model.scaffold_916.20 | 0.4 | 95 | 78.2 | 6.00E-15 | Cysteine proteinase 6 OS=Dictyostelium discoideum GN=cprF PE=2 SV=1 |
| TF319691 | evm.model.scaffold_824.8 | 0.49 | 59 | 72.8 | 1.00E-15 | Protein glass OS=Drosophila virilis GN=gl PE=3 SV=1 |
| TF320810 | evm.model.scaffold_617.1 | 0.67 | 296 | 452 | 1.00E-155 | Serine/threonine-protein kinase prk-2 OS=Caenorhabditis elegans GN=prk-2 PE=3 SV=2 |
| TF321616 | evm.model.scaffold_29.18.1 | 0.78 | 337 | 353 | 7.00E-112 | hypothetical protein D910_07042 [Dendroctonus ponderosae] |
| TF322436 | evm.model.scaffold_776.1_evm.model.scaffold_776.2 | 0.33 | 678 | 316 | 2.00E-90 | Agrin OS=Gallus gallus GN=AGRN PE=1 SV=3 |
| TF322869 | evm.model.scaffold_116.38 | 0.48 | 52 | 57.8 | 1.00E-11 | BBSome-interacting protein 1 OS=Homo sapiens GN=BBIP1 PE=1 SV=2 |
| TF324711 | evm.model.scaffold_673.42 | 0.41 | 104 | 78.2 | 8.00E-19 | Ribonuclease P protein subunit p14 OS=Pongo abelii GN=RPP14 PE=2 SV=3 |
| TF324725 | evm.model.scaffold_773.30 | 0.39 | 438 | 321 | 3.00E-96 | AT-rich interactive domain-containing protein 5B OS=Bos taurus GN=ARID5B PE=3 SV=1 |
| TF326574 | evm.model.scaffold_776.245 | 0.48 | 95 | 74.7 | 6.00E-15 | myosuppressin [Tribolium castaneum] |
| TF326706 | evm.model.scaffold_1.99 | 0.4 | 344 | 205 | 6.00E-58 | uncharacterized LOC662455; K19347 SUN domain-containing protein 1/2 (A) |
| TF326877 | evm.model.scaffold_483.5 | 0.30 | 227 | 100 | 8.00E-20 | PREDICTED: transcriptional regulator ATRX homolog [Tribolium castaneum] |
| TF326912 | evm.model.scaffold_86.13 | 0.46 | 67 | 68.2 | 8.00E-13 | PREDICTED: uncharacterized protein LOC103314868 [Tribolium castaneum] |
| TF327334 | evm.model.scaffold_404.2 | 0.96 | 188 | 242 | 1.00E-76 | hypothetical protein YQE_05157, partial [Dendroctonus ponderosae] |
| TF327334 | evm.model.scaffold_67.27 | 0.96 | 188 | 242 | 1.00E-76 | hypothetical protein YQE_05157, partial [Dendroctonus ponderosae] |
| TF327334 | evm.model.scaffold_806.2 | 0.96 | 188 | 242 | 1.00E-76 | hypothetical protein YQE_05157, partial [Dendroctonus ponderosae] |
| TF327392 | evm.model.scaffold_752.28 | 0.4 | 187 | 142 | 1.00E-38 | PREDICTED: uncharacterized protein LOC658581 [Tribolium castaneum] |
| TF327598 | evm.model.scaffold_842.15 | 0.60 | 101 | 146 | 5.00E-37 | PREDICTED: uncharacterized protein LOC101737721 [Bombyx mori] |
| TF327814 | evm.model.scaffold_195.45 | 0.37 | 53 | 49.7 | 2.00E-07 | PR domain zinc finger protein 16 OS=Mus musculus GN=Prdm16 PE=1 SV=1 |
| TF328580 | evm.model.scaffold_913.23 | 0.5 | 65 | 69.3 | 8.00E-14 | E3 ubiquitin-protein ligase RNF180 OS=Homo sapiens GN=RNF180 PE=2 SV=2 |
| TF328613 | evm.model.scaffold_97.27 | 0.48 | 89 | 76.3 | 2.00E-18 | SOSS complex subunit C OS=Homo sapiens GN=INIP PE=1 SV=1 |
| TF329295 | evm.model.scaffold_387.3 | 0.5 | 106 | 109 | 6.00E-26 | Galectin-3-binding protein A OS=Danio rerio GN=lgals3bpa PE=2 SV=1 |
| TF329520 | evm.model.scaffold_195.9 | NA | NA | NA | NA | No blast hit |
| TF329698 | evm.model.scaffold_109.9 | 0.3 | 637 | 52.8 | 6.00E-05 | Flagellar attachment zone protein 1 OS=Trypanosoma brucei brucei (strain 927/4 GUTat10.1) GN=FAZ1 PE=4 SV=1 |
| TF330455 | evm.model.scaffold_57.111 | 0.51 | 58 | 69.3 | 1.00E-14 | Trypsin-1 OS=Anopheles gambiae GN=TRYP1 PE=2 SV=3 |
| TF330455 | evm.model.scaffold_814.7 | 0.41 | 245 | 167 | 1.00E-49 | Chymotrypsin BI OS=Litopenaeus vannamei PE=1 SV=1 |
| TF331065 | evm.model.scaffold_215.150 | 0.32 | 182 | 95.9 | 4.00E-23 | Arginine esterase OS=Canis familiaris PE=1 SV=1 |
| TF332134 | evm.model.scaffold_400.105 | 0.82 | 824 | 1389 | 0 | hypothetical protein D910_02964 [Dendroctonus ponderosae] |
| TF332703 | evm.model.scaffold_1.45.1 | NA | NA | NA | NA | No blast hit |
| TF332751 | evm.model.scaffold_85.57 | 0.5 | 327 | 334 | 1.00E-96 | C-myc promoter-binding protein OS=Homo sapiens GN=DENND4A PE=1 SV=2 |
| TF332943 | evm.model.scaffold_37.116 | 0.3 | 154 | 51.6 | 9.00E-08 | Biogenesis of lysosome-related organelles complex 1 subunit 5 OS=Drosophila melanogaster GN=muted PE=1 SV=1 |
| TF333013 | evm.model.scaffold_390.41 | 0.46 | 47 | 52 | 9.00E-09 | Mitotic-spindle organizing protein 2B OS=Xenopus tropicalis GN=mzt2b PE=2 SV=1 |
| TF333633 | evm.model.scaffold_85.77 | 0.41 | 915 | 653 | 0 | Fat-like cadherin-related tumor suppressor homolog OS=Drosophila melanogaster GN=kug PE=2 SV=3 |
| TF333805 | evm.model.scaffold_59.230 | 0.77 | 56 | 87 | 1.00E-19 | hypothetical protein YQE_00300, partial [Dendroctonus ponderosae] |
| TF336561 | evm.model.scaffold_35.61 | 0.34 | 749 | 326 | 1.00E-90 | hypothetical protein TcasGA2_TC007806 [Tribolium castaneum] |
| TF337913 | evm.model.scaffold_195.60 | 0.6 | 53 | 82 | 6.00E-19 | Longitudinals lacking protein, isoforms A/B/D/L OS=Drosophila melanogaster GN=lola PE=1 SV=1 |
| TF338163 | evm.model.scaffold_608.85 | 0.37 | 75 | 50.1 | 2.00E-08 | Uncharacterized protein NCBP2-AS2 homolog OS=Ixodes scapularis GN=ISCW010018 PE=4 SV=1 |
| TF338674 | evm.model.scaffold_162.21 | 0.44 | 61 | 48.5 | 6.00E-06 | TRIM4; tripartite motif containing 4; K11998 tripartite motif-containing protein 4 [EC:2.3.2.27] (A) |
| TF338735 | evm.model.scaffold_62.167 | 0.46 | 92 | 81.3 | 1.00E-19 | Probable phospholipid hydroperoxide glutathione peroxidase OS=Helianthus annuus GN=GPXHA-2 PE=2 SV=1 |
| TF339848 | evm.model.scaffold_195.51 | 0.36 | 76 | 55.8 | 1.00E-07 | Longitudinals lacking protein, isoforms A/B/D/L OS=Drosophila melanogaster GN=lola PE=1 SV=1 |
| TF342569 | evm.model.scaffold_390.33.2 | 0.46 | 84 | 90.9 | 6.00E-19 | E3 ubiquitin-protein ligase RNF8 OS=Mus musculus GN=Rnf8 PE=1 SV=1 |
| TF342682 | evm.model.scaffold_104.52 | 0.22 | 608 | 87.4 | 3.00E-15 | tRNA nuclease WapA OS=Bacillus subtilis (strain 168) GN=wapA PE=1 SV=2 |
| TF342862 | evm.model.scaffold_423.2 | 0.31 | 92 | 43.9 | 4.00E-05 | Nuclear anchorage protein 1 OS=Caenorhabditis elegans GN=anc-1 PE=1 SV=3 |
| TF343041 | evm.model.scaffold_474.13 | 0.38 | 392 | 241 | 1.00E-68 | Cilia- and flagella-associated protein 57 OS=Mus musculus GN=Cfap57 PE=2 SV=3 |
| TF343331 | evm.model.scaffold_51.120 | 0.84 | 87 | 144 | 1.00E-38 | PREDICTED: probable G-protein coupled receptor Mth-like 3 [Tribolium castaneum] |
| TF343455 | evm.model.scaffold_100.20 | 0.54 | 137 | 164 | 2.00E-44 | ALK tyrosine kinase receptor; K05119 anaplastic lymphoma kinase [EC:2.7.10.1] (A) |
| TF343529 | evm.model.scaffold_890.94 | 0.22 | 245 | 62 | 1.00E-08 | TcGr87, gr57; gustatory receptor candidate 57; K08471 gustatory receptor (A) |
| TF343614 | evm.model.scaffold_215.39 | 0.88 | 248 | 461 | 8.00E-158 | hypothetical protein YQE_08547, partial [Dendroctonus ponderosae] |
| TF343944 | evm.model.scaffold_10.82 | 0.26 | 424 | 128 | 4.00E-31 | putative protein MSS51 homolog, mitochondrial; K17656 mitochondrial splicing suppressor protein 51 (A) |
| TF344078 | evm.model.scaffold_149.2 | 0.89 | 83 | 155 | 7.00E-46 | hypothetical protein YQE_05541, partial [Dendroctonus ponderosae] |
| TF344078 | evm.model.scaffold_55.164 | 0.32 | 111 | 48.1 | 3.00E-06 | nfd102, GRMZM2G145968, HMGe; nucleosome/chromatin assembly factor D; K11296 high mobility group protein B3 (A) |
| TF344182 | evm.model.scaffold_62.77 | 0.49 | 85 | 79.7 | 4.00E-20 | Molybdopterin synthase sulfur carrier subunit OS=Anopheles gambiae GN=Mocs2 PE=3 SV=1 |
| TF344183 | evm.model.scaffold_467.29 | 0.27 | 250 | 52 | 1.00E-05 | Histone-lysine N-methyltransferase 2C OS=Mus musculus GN=Kmt2c PE=1 SV=2 |
| TF344183 | evm.model.scaffold_467.30 | 0.93 | 176 | 340 | 2.00E-104 | hypothetical protein D910_06998 [Dendroctonus ponderosae] |
| TF344183 | evm.model.scaffold_467.31 | 0.46 | 154 | 148 | 5.00E-40 | Histone-lysine N-methyltransferase 2D OS=Homo sapiens GN=KMT2D PE=1 SV=2 |
| TF344183 | evm.model.scaffold_467.32 | 0.48 | 43 | 48.9 | 6.00E-05 | Histone-lysine N-methyltransferase 2C OS=Mus musculus GN=Kmt2c PE=1 SV=2 |
| TF344183 | evm.model.scaffold_524.49 | 0.31 | 971 | 432 | 1.00E-121 | Histone-lysine N-methyltransferase 2C OS=Homo sapiens GN=KMT2C PE=1 SV=3 |
| TF350043 | evm.model.scaffold_55.157 | 0.45 | 177 | 100 | 2.00E-21 | hypothetical protein TcasGA2_TC005600 [Tribolium castaneum] |
| TF350345 | evm.model.scaffold_52.17 | 0.31 | 121 | 60.1 | 2.00E-09 | Potassium channel subfamily K member 4 OS=Homo sapiens GN=KCNK4 PE=1 SV=2 |
| TF350806 | evm.model.scaffold_53.126 | 0.85 | 163 | 286 | 1.00E-93 | hypothetical protein YQE_11471, partial [Dendroctonus ponderosae] |
| TF350820 | evm.model.scaffold_102.84 | 0.52 | 89 | 100 | 2.00E-20 | hypothetical protein YQE_07110, partial [Dendroctonus ponderosae] |
| TF350820 | evm.model.scaffold_656.44 | 0.72 | 644 | 946 | 0 | hypothetical protein YQE_04020, partial [Dendroctonus ponderosae] |
| TF350860 | evm.model.scaffold_195.42 | 0.4 | 57 | 51.6 | 2.00E-07 | Zinc finger protein 711 OS=Danio rerio GN=znf711 PE=1 SV=1 |
| TF350935 | evm.model.scaffold_490.7 | 0.38 | 67 | 51.6 | 8.00E-08 | Zinc finger protein 69 OS=Homo sapiens GN=ZNF69 PE=1 SV=2 |
| TF351725 | evm.model.scaffold_71.31 | 0.41 | 55 | 51.6 | 2.00E-06 | Cysteine and glycine-rich protein 1 OS=Coturnix coturnix japonica GN=CSRP1 PE=2 SV=2 |
| TF352005 | evm.model.scaffold_745.4 | 0.54 | 507 | 346 | 1.00E-104 | Protein dachsous OS=Drosophila melanogaster GN=ds PE=1 SV=3 |
| TF352176 | evm.model.scaffold_1.21 | 0.54 | 136 | 154 | 3.00E-44 | Polypeptide N-acetylgalactosaminyltransferase 5 OS=Drosophila melanogaster GN=pgant5 PE=2 SV=2 |
| TF352368 | evm.model.scaffold_774.10 | 0.26 | 509 | 178 | 8.00E-47 | Putative UDP-glucuronosyltransferase ugt-47 OS=Caenorhabditis elegans GN=ugt-47 PE=1 SV=2 |
| TF352368 | evm.model.scaffold_774.11 | 0.28 | 494 | 196 | 2.00E-54 | Putative UDP-glucuronosyltransferase ugt-47 OS=Caenorhabditis elegans GN=ugt-47 PE=1 SV=2 |
| TF352374 | evm.model.scaffold_842.4 | 0.87 | 109 | 194 | 4.00E-59 | ATP synthase subunit beta, mitochondrial (Fragment) OS=Drosophila virilis GN=ATPsyn-beta PE=3 SV=1 |
| TF352387 | evm.model.scaffold_51.52 | 0.99 | 116 | 236 | 2.00E-70 | hypothetical protein YQE_02070, partial [Dendroctonus ponderosae] |
| TF354245 | evm.model.scaffold_3.58 | 0.48 | 91 | 103 | 2.00E-25 | Putative ATP-dependent RNA helicase DHX33 OS=Mus musculus GN=Dhx33 PE=1 SV=1 |
| TF354341 | evm.model.scaffold_94.200 | 0.3 | 119 | 49.7 | 7.00E-07 | ERAD-associated E3 ubiquitin-protein ligase HRD1A OS=Arabidopsis thaliana GN=HRD1A PE=2 SV=1 |
