# Supplementary material for: Chromosome-level genome assembly and population genomic analyses provide insights into adaptive evolution of the red turpentine beetle, Dendroctonus valens
Source: BMC Biol. 2022 Aug 24;20:190. doi: 10.1186/s12915-022-01388-y (PMC9400205; doi:10.1186/s12915-022-01388-y)
Supplement: Supplementary file 1 — Additional file 1: Table S1. Summary statistics of genome sequencing data of Dendroctonus valens. Table S2. Summary statistics of genome assembly of Dendroctonus valens. Table S3. BUSCO evaluation result for genome assembly of Dendroctonus valens. Table S4. Summary statistics of transposable elements in Dendroctonus valens genome. Table S5. Summary of gene families manually curated in Dendroctonus valens genome. Table S6. Summary statistics of genome annotation in Dendroctonus valens genome. Table S7. List of gene families that are unique in Dendroctonus valens compared to other three Coleoptera species. Table S8. Gene families that are rapidly expanded in Dendroctonus valens revealed by CAFE analysis. Table S9. Gene families that are rapidly contracted in Dendroctonus valens revealed by CAFE analysis. Table S10. List of genes that are positively selected in Dendroctonus valens revealed by codeml analysis. Table S11. Gene ontology enrichment result of positively selected genes in Dendroctonus valens. Table S12. Sampling site information for genome resequencing of geographical populations. Table S13. Summary statistics of genome resequencing data in different populations. Table S14. List of genes that undergo selective sweep in the China population compared to CAMT population. [file 12915_2022_1388_MOESM1_ESM.zip › Table S8.docx]

| **Table S8** List of gene families that are rapidly expanded in *Dendroctonus valens* revealed by CAFÉ analysis | | | | | | |
| --- | --- | --- | --- | --- | --- | --- |
| **TreeFam ID** | **Gene ID** | **Identity** | **Aligned length** | **Score** | **E-value** | **Description** |
| TF101165 | evm.model.scaffold_42.15 | 0.77 | 224 | 367 | 1.00E-116 | Dynein heavy chain, cytoplasmic OS=Drosophila melanogaster GN=Dhc64C PE=2 SV=2 |
| TF101165 | evm.model.scaffold_42.16 | 0.81 | 903 | 1540 | 0 | Dynein heavy chain, cytoplasmic OS=Drosophila melanogaster GN=Dhc64C PE=2 SV=2 |
| TF101165 | evm.model.scaffold_42.17 | 0.79 | 118 | 195 | 6.00E-58 | Dynein heavy chain, cytoplasmic OS=Drosophila melanogaster GN=Dhc64C PE=2 SV=2 |
| TF101165 | evm.model.scaffold_42.18 | 0.86 | 1261 | 2277 | 0 | Dynein heavy chain, cytoplasmic OS=Drosophila melanogaster GN=Dhc64C PE=2 SV=2 |
| TF101165 | evm.model.scaffold_42.19 | 0.83 | 298 | 528 | 1.00E-171 | Dynein heavy chain, cytoplasmic OS=Drosophila melanogaster GN=Dhc64C PE=2 SV=2 |
| TF101165 | evm.model.scaffold_42.20 | 0.77 | 1154 | 1877 | 0 | Dynein heavy chain, cytoplasmic OS=Drosophila melanogaster GN=Dhc64C PE=2 SV=2 |
| TF101165 | evm.model.scaffold_42.21 | 0.59 | 176 | 224 | 2.00E-65 | Dynein heavy chain, cytoplasmic OS=Drosophila melanogaster GN=Dhc64C PE=2 SV=2 |
| TF101217 | evm.model.scaffold_17.12 | 0.23 | 542 | 57.4 | 6.00E-05 | PREDICTED: myosin-10-like [Orussus abietinus] |
| TF101217 | evm.model.scaffold_24.205 | 0.33 | 487 | 185 | 3.00E-46 | golgin subfamily B member 1-like; K08838 serine/threonine-protein kinase 24/25/MST4 [EC:2.7.11.1] (A) |
| TF101217 | evm.model.scaffold_247.1 | 0.33 | 285 | 140 | 1.00E-32 | DNA repair protein RAD50 OS=Drosophila melanogaster GN=rad50 PE=2 SV=4 |
| TF101217 | evm.model.scaffold_247.2 | 0.27 | 903 | 281 | 3.00E-78 | DNA repair protein RAD50 OS=Mus musculus GN=Rad50 PE=1 SV=1 |
| TF101217 | evm.model.scaffold_251.29 | 0.37 | 366 | 241 | 1.00E-67 | DNA repair protein RAD50 OS=Drosophila melanogaster GN=rad50 PE=2 SV=4 |
| TF101217 | evm.model.scaffold_251.30 | 0.31 | 721 | 306 | 3.00E-89 | DNA repair protein RAD50 OS=Homo sapiens GN=RAD50 PE=1 SV=1 |
| TF101217 | evm.model.scaffold_315.23 | 0.34 | 239 | 101 | 2.00E-19 | Protein lava lamp OS=Drosophila melanogaster GN=lva PE=1 SV=2 |
| TF101217 | evm.model.scaffold_471.160 | 0.33 | 272 | 84.3 | 7.00E-15 | Protein lava lamp OS=Drosophila melanogaster GN=lva PE=1 SV=2 |
| TF300834 | evm.model.scaffold_26.46 | 0.29 | 371 | 151 | 3.00E-41 | Malate dehydrogenase, mitochondrial OS=Sus scrofa GN=MDH2 PE=1 SV=2 |
| TF300834 | evm.model.scaffold_26.50 | 0.24 | 283 | 95.1 | 3.00E-20 | Malate dehydrogenase, mitochondrial OS=Macaca fascicularis GN=MDH2 PE=2 SV=1 |
| TF300834 | evm.model.scaffold_28.8 | 0.67 | 335 | 456 | 1.00E-161 | Malate dehydrogenase, mitochondrial OS=Pongo abelii GN=MDH2 PE=2 SV=1 |
| TF300834 | evm.model.scaffold_31.268 | 0.41 | 293 | 235 | 2.00E-74 | Malate dehydrogenase, mitochondrial OS=Pongo abelii GN=MDH2 PE=2 SV=1 |
| TF300834 | evm.model.scaffold_47.21 | 0.27 | 323 | 126 | 6.00E-32 | Malate dehydrogenase, mitochondrial OS=Homo sapiens GN=MDH2 PE=1 SV=3 |
| TF300834 | evm.model.scaffold_47.27 | 0.27 | 323 | 126 | 6.00E-32 | Malate dehydrogenase, mitochondrial OS=Homo sapiens GN=MDH2 PE=1 SV=3 |
| TF300834 | evm.model.scaffold_71.57 | 0.64 | 314 | 414 | 1.00E-144 | Malate dehydrogenase, mitochondrial OS=Pongo abelii GN=MDH2 PE=2 SV=1 |
| TF300834 | evm.model.scaffold_842.60 | 0.49 | 336 | 317 | 1.00E-106 | Malate dehydrogenase, mitochondrial OS=Pongo abelii GN=MDH2 PE=2 SV=1 |
| TF300839 | evm.model.scaffold_653.43 | 0.57 | 509 | 602 | 0 | Alanine aminotransferase 2 OS=Xenopus laevis GN=gpt2 PE=2 SV=1 |
| TF300839 | evm.model.scaffold_9.73 | 0.56 | 526 | 602 | 0 | Alanine aminotransferase 2 OS=Xenopus laevis GN=gpt2 PE=2 SV=1 |
| TF300839 | evm.model.scaffold_9.87 | 0.64 | 349 | 461 | 1.00E-159 | Alanine aminotransferase 2-like OS=Danio rerio GN=gpt2l PE=2 SV=2 |
| TF300839 | evm.model.scaffold_9.88 | 0.58 | 105 | 142 | 3.00E-40 | Alanine aminotransferase 2 OS=Mus musculus GN=Gpt2 PE=1 SV=1 |
| TF300912 | evm.model.scaffold_14.21 | 0.58 | 147 | 179 | 1.00E-56 | Calmodulin OS=Euglena gracilis PE=1 SV=2 |
| TF300912 | evm.model.scaffold_14.33 | 0.58 | 147 | 179 | 2.00E-56 | Calmodulin OS=Euglena gracilis PE=1 SV=2 |
| TF300912 | evm.model.scaffold_291.6 | 0.75 | 136 | 215 | 2.00E-68 | Calmodulin OS=Electrophorus electricus GN=calm PE=2 SV=2 |
| TF300912 | evm.model.scaffold_310.10 | 0.43 | 138 | 108 | 2.00E-29 | Calmodulin OS=Renilla reniformis PE=1 SV=2 |
| TF300912 | evm.model.scaffold_32.36 | 0.98 | 83 | 170 | 7.00E-55 | Calmodulin OS=Strongylocentrotus intermedius PE=2 SV=3 |
| TF300912 | evm.model.scaffold_32.61 | 0.98 | 83 | 170 | 7.00E-55 | Calmodulin OS=Strongylocentrotus intermedius PE=2 SV=3 |
| TF300912 | evm.model.scaffold_60.1 | 0.75 | 136 | 215 | 1.00E-69 | Calmodulin OS=Electrophorus electricus GN=calm PE=2 SV=2 |
| TF313535 | evm.model.scaffold_11.61 | 0.32 | 475 | 263 | 1.00E-80 | Putative inorganic phosphate cotransporter OS=Drosophila ananassae GN=Picot PE=3 SV=1 |
| TF313535 | evm.model.scaffold_112.7 | 0.49 | 455 | 449 | 1.00E-151 | Vesicular glutamate transporter 2 OS=Homo sapiens GN=SLC17A6 PE=2 SV=1 |
| TF313535 | evm.model.scaffold_13.38 | 0.26 | 404 | 143 | 2.00E-36 | Sodium-dependent phosphate transport protein 1 OS=Oryctolagus cuniculus GN=SLC17A1 PE=2 SV=1 |
| TF313535 | evm.model.scaffold_133.1.1 | 0.41 | 411 | 307 | 1.00E-91 | Putative inorganic phosphate cotransporter OS=Drosophila ananassae GN=Picot PE=3 SV=1 |
| TF313535 | evm.model.scaffold_133.2 | 0.36 | 457 | 314 | 1.00E-101 | Putative inorganic phosphate cotransporter OS=Drosophila ananassae GN=Picot PE=3 SV=1 |
| TF313535 | evm.model.scaffold_133.3 | 0.34 | 483 | 314 | 1.00E-100 | Putative inorganic phosphate cotransporter OS=Drosophila melanogaster GN=Picot PE=1 SV=1 |
| TF313535 | evm.model.scaffold_133.5 | 0.3 | 459 | 233 | 2.00E-69 | Sialin OS=Ovis aries GN=SLC17A5 PE=2 SV=1 |
| TF313535 | evm.model.scaffold_15.17 | 0.32 | 494 | 267 | 2.00E-82 | Sialin OS=Homo sapiens GN=SLC17A5 PE=1 SV=2 |
| TF313535 | evm.model.scaffold_214.15.1 | 0.41 | 459 | 344 | 1.00E-112 | Putative inorganic phosphate cotransporter OS=Drosophila ananassae GN=Picot PE=3 SV=1 |
| TF313535 | evm.model.scaffold_214.16 | 0.31 | 468 | 279 | 4.00E-87 | Putative inorganic phosphate cotransporter OS=Drosophila melanogaster GN=Picot PE=1 SV=1 |
| TF313535 | evm.model.scaffold_214.18 | 0.31 | 517 | 262 | 3.00E-80 | Putative inorganic phosphate cotransporter OS=Drosophila melanogaster GN=Picot PE=1 SV=1 |
| TF313535 | evm.model.scaffold_214.19 | 0.32 | 495 | 271 | 5.00E-84 | Putative inorganic phosphate cotransporter OS=Drosophila melanogaster GN=Picot PE=1 SV=1 |
| TF313535 | evm.model.scaffold_214.20 | 0.37 | 134 | 98.6 | 2.00E-21 | Putative inorganic phosphate cotransporter OS=Drosophila ananassae GN=Picot PE=3 SV=1 |
| TF313535 | evm.model.scaffold_214.21 | 0.34 | 483 | 315 | 1.00E-101 | Putative inorganic phosphate cotransporter OS=Drosophila melanogaster GN=Picot PE=1 SV=1 |
| TF313535 | evm.model.scaffold_214.23 | 0.3 | 459 | 233 | 2.00E-69 | Sialin OS=Ovis aries GN=SLC17A5 PE=2 SV=1 |
| TF313535 | evm.model.scaffold_214.24 | 0.32 | 399 | 204 | 2.00E-59 | Sialin OS=Ovis aries GN=SLC17A5 PE=2 SV=1 |
| TF313535 | evm.model.scaffold_25.9 | 0.3 | 459 | 220 | 4.00E-62 | Sialin OS=Ovis aries GN=SLC17A5 PE=2 SV=1 |
| TF313535 | evm.model.scaffold_3.33 | 0.31 | 446 | 216 | 9.00E-64 | Putative inorganic phosphate cotransporter OS=Drosophila ananassae GN=Picot PE=3 SV=1 |
| TF313535 | evm.model.scaffold_3.34 | 0.29 | 480 | 224 | 5.00E-66 | Putative inorganic phosphate cotransporter OS=Drosophila melanogaster GN=Picot PE=1 SV=1 |
| TF313535 | evm.model.scaffold_3.35 | 0.31 | 475 | 258 | 2.00E-79 | Putative inorganic phosphate cotransporter OS=Drosophila ananassae GN=Picot PE=3 SV=1 |
| TF313535 | evm.model.scaffold_324.109 | 0.47 | 180 | 142 | 3.00E-36 | hypothetical protein D910_03214 [Dendroctonus ponderosae] |
| TF313535 | evm.model.scaffold_33.6 | 0.3 | 192 | 84.3 | 2.00E-16 | Putative inorganic phosphate cotransporter OS=Drosophila melanogaster GN=Picot PE=1 SV=1 |
| TF313535 | evm.model.scaffold_463.158 | 0.34 | 466 | 278 | 1.00E-86 | Sialin OS=Ovis aries GN=SLC17A5 PE=2 SV=1 |
| TF313535 | evm.model.scaffold_47.6 | 0.28 | 468 | 216 | 2.00E-63 | Putative inorganic phosphate cotransporter OS=Drosophila ananassae GN=Picot PE=3 SV=1 |
| TF313535 | evm.model.scaffold_509.46 | 0.4 | 269 | 188 | 2.00E-52 | Sialin OS=Ovis aries GN=SLC17A5 PE=2 SV=1 |
| TF313535 | evm.model.scaffold_524.11 | 0.26 | 404 | 143 | 2.00E-36 | Sodium-dependent phosphate transport protein 1 OS=Oryctolagus cuniculus GN=SLC17A1 PE=2 SV=1 |
| TF313535 | evm.model.scaffold_55.129 | 0.37 | 460 | 323 | 1.00E-104 | Sialin OS=Homo sapiens GN=SLC17A5 PE=1 SV=2 |
| TF313535 | evm.model.scaffold_566.20 | 0.38 | 488 | 330 | 1.00E-107 | Sialin OS=Homo sapiens GN=SLC17A5 PE=1 SV=2 |
| TF313535 | evm.model.scaffold_566.21 | 0.43 | 445 | 390 | 1.00E-129 | Vesicular glutamate transporter 1 OS=Xenopus laevis GN=slc17a7 PE=2 SV=1 |
| TF313535 | evm.model.scaffold_675.12 | 0.32 | 474 | 235 | 1.00E-69 | Vesicular glutamate transporter 2 OS=Rattus norvegicus GN=Slc17a6 PE=1 SV=1 |
| TF313535 | evm.model.scaffold_675.13 | 0.34 | 467 | 283 | 8.00E-89 | Putative inorganic phosphate cotransporter OS=Drosophila ananassae GN=Picot PE=3 SV=1 |
| TF313535 | evm.model.scaffold_73.18 | 0.35 | 458 | 280 | 8.00E-88 | Putative inorganic phosphate cotransporter OS=Drosophila ananassae GN=Picot PE=3 SV=1 |
| TF313535 | evm.model.scaffold_73.19.1 | 0.36 | 460 | 318 | 1.00E-103 | Putative inorganic phosphate cotransporter OS=Drosophila ananassae GN=Picot PE=3 SV=1 |
| TF313535 | evm.model.scaffold_73.20 | 0.35 | 482 | 313 | 1.00E-100 | Putative inorganic phosphate cotransporter OS=Drosophila melanogaster GN=Picot PE=1 SV=1 |
| TF313535 | evm.model.scaffold_751.5 | 0.43 | 413 | 368 | 1.00E-122 | Vesicular glutamate transporter 2.2 OS=Danio rerio GN=slc17a6a PE=2 SV=1 |
| TF313535 | evm.model.scaffold_751.6 | 0.37 | 488 | 293 | 9.00E-93 | Sialin OS=Homo sapiens GN=SLC17A5 PE=1 SV=2 |
| TF313535 | evm.model.scaffold_818.31 | 0.32 | 297 | 146 | 6.00E-39 | Sialin OS=Ovis aries GN=SLC17A5 PE=2 SV=1 |
| TF313535 | evm.model.scaffold_818.32 | 0.98 | 61 | 124 | 1.00E-31 | unknown [Dendroctonus ponderosae] |
| TF313535 | evm.model.scaffold_85.115 | 0.62 | 492 | 614 | 0 | Putative inorganic phosphate cotransporter OS=Drosophila melanogaster GN=Picot PE=1 SV=1 |
| TF313535 | evm.model.scaffold_890.146 | 0.67 | 470 | 642 | 0 | Putative inorganic phosphate cotransporter OS=Drosophila melanogaster GN=Picot PE=1 SV=1 |
| TF313535 | evm.model.scaffold_898.20 | 0.42 | 461 | 358 | 1.00E-118 | Sialin OS=Homo sapiens GN=SLC17A5 PE=1 SV=2 |
| TF313535 | evm.model.scaffold_9.1 | 0.37 | 371 | 250 | 1.00E-77 | Sialin OS=Homo sapiens GN=SLC17A5 PE=1 SV=2 |
| TF313535 | evm.model.scaffold_920.1 | 0.39 | 504 | 333 | 1.00E-105 | Vesicular glutamate transporter 3 OS=Homo sapiens GN=SLC17A8 PE=1 SV=1 |
| TF313535 | evm.model.scaffold_93.47 | 0.78 | 46 | 80.9 | 2.00E-17 | sialin; K12301 MFS transporter, ACS family, solute carrier family 17 (sodium-dependent inorganic phosphate cotransporter), member 5 (A) |
| TF313535 | evm.model.scaffold_93.83 | 0.36 | 476 | 306 | 7.00E-98 | Sialin OS=Homo sapiens GN=SLC17A5 PE=1 SV=2 |
| TF313535 | evm.model.scaffold_99.164 | 0.26 | 382 | 159 | 6.00E-42 | Sialin OS=Homo sapiens GN=SLC17A5 PE=1 SV=2 |
| TF313573 | evm.model.scaffold_67.37 | 0.36 | 112 | 62.8 | 1.00E-09 | Transcription initiation factor TFIID subunit 1 OS=Homo sapiens GN=TAF1 PE=1 SV=2 |
| TF313573 | evm.model.scaffold_703.36 | 0.6 | 1672 | 1907 | 0 | Transcription initiation factor TFIID subunit 1 OS=Drosophila melanogaster GN=Taf1 PE=1 SV=3 |
| TF313573 | evm.model.scaffold_81.108 | 0.47 | 762 | 596 | 0 | Transcription initiation factor TFIID subunit 1 OS=Drosophila melanogaster GN=Taf1 PE=1 SV=3 |
| TF313573 | evm.model.scaffold_81.109 | 0.72 | 110 | 163 | 5.00E-46 | Transcription initiation factor TFIID subunit 1 OS=Drosophila melanogaster GN=Taf1 PE=1 SV=3 |
| TF313573 | evm.model.scaffold_81.110 | 0.48 | 198 | 157 | 2.00E-43 | Transcription initiation factor TFIID subunit 1 OS=Drosophila melanogaster GN=Taf1 PE=1 SV=3 |
| TF313836 | evm.model.scaffold_502.20 | 0.5 | 204 | 228 | 8.00E-70 | Choline O-acetyltransferase OS=Drosophila melanogaster GN=Cha PE=1 SV=3 |
| TF313836 | evm.model.scaffold_502.21 | 0.49 | 169 | 168 | 5.00E-48 | Choline O-acetyltransferase OS=Drosophila melanogaster GN=Cha PE=1 SV=3 |
| TF313836 | evm.model.scaffold_502.22 | 0.53 | 143 | 146 | 3.00E-40 | Choline O-acetyltransferase OS=Drosophila melanogaster GN=Cha PE=1 SV=3 |
| TF313836 | evm.model.scaffold_561.67 | 0.5 | 497 | 521 | 1.00E-178 | Carnitine O-palmitoyltransferase 1, muscle isoform OS=Homo sapiens GN=CPT1B PE=1 SV=2 |
| TF313836 | evm.model.scaffold_62.13 | 0.49 | 560 | 565 | 0 | Carnitine O-palmitoyltransferase 1, muscle isoform OS=Homo sapiens GN=CPT1B PE=1 SV=2 |
| TF314604 | evm.model.scaffold_109.47 | 0.48 | 1094 | 1004 | 0 | Cohesin subunit SA-1 OS=Homo sapiens GN=STAG1 PE=1 SV=3 |
| TF314604 | evm.model.scaffold_632.10 | 0.49 | 175 | 174 | 6.00E-49 | Cohesin subunit SA-1 OS=Homo sapiens GN=STAG1 PE=1 SV=3 |
| TF314604 | evm.model.scaffold_632.7 | 0.6 | 83 | 120 | 8.00E-32 | Cohesin subunit SA-2 OS=Mus musculus GN=Stag2 PE=1 SV=3 |
| TF314604 | evm.model.scaffold_632.8 | 0.67 | 116 | 167 | 4.00E-48 | Cohesin subunit SA-2 OS=Homo sapiens GN=STAG2 PE=1 SV=3 |
| TF314604 | evm.model.scaffold_632.9 | 0.41 | 360 | 273 | 2.00E-81 | Cohesin subunit SA-1 OS=Homo sapiens GN=STAG1 PE=1 SV=3 |
| TF314604 | evm.model.scaffold_724.3 | 0.64 | 370 | 478 | 1.00E-157 | Cohesin subunit SA-1 OS=Homo sapiens GN=STAG1 PE=1 SV=3 |
| TF314604 | evm.model.scaffold_724.4 | 0.39 | 248 | 179 | 7.00E-50 | Cohesin subunit SA-1 OS=Homo sapiens GN=STAG1 PE=1 SV=3 |
| TF314604 | evm.model.scaffold_724.5 | 0.4 | 276 | 194 | 5.00E-55 | Cohesin subunit SA-2 OS=Homo sapiens GN=STAG2 PE=1 SV=3 |
| TF314604 | evm.model.scaffold_818.17 | 0.22 | 1006 | 148 | 4.00E-34 | cohesin subunit SA-2-like; K06671 cohesin complex subunit SA-1/2 (A) |
| TF314604 | evm.model.scaffold_818.30 | 0.23 | 736 | 136 | 1.00E-30 | cohesin subunit SA-2-like; K06671 cohesin complex subunit SA-1/2 (A) |
| TF315192 | evm.model.scaffold_11.85.1 | 0.45 | 780 | 689 | 0 | Endothelin-converting enzyme 1 OS=Mus musculus GN=Ece1 PE=1 SV=1 |
| TF315192 | evm.model.scaffold_37.414 | 0.39 | 623 | 444 | 1.00E-140 | Membrane metallo-endopeptidase-like 1 OS=Mus musculus GN=Mmel1 PE=1 SV=1 |
| TF315192 | evm.model.scaffold_400.192 | 0.22 | 735 | 147 | 2.00E-35 | Endothelin-converting enzyme 1 OS=Rattus norvegicus GN=Ece1 PE=1 SV=2 |
| TF315192 | evm.model.scaffold_400.193 | 0.2 | 714 | 81.3 | 2.00E-14 | Endothelin-converting enzyme 2 OS=Mus musculus GN=Ece2 PE=2 SV=2 |
| TF315192 | evm.model.scaffold_400.194 | 0.21 | 726 | 125 | 2.00E-28 | Endothelin-converting enzyme 1 OS=Mus musculus GN=Ece1 PE=1 SV=1 |
| TF315192 | evm.model.scaffold_400.195 | 0.22 | 719 | 143 | 3.00E-34 | Endothelin-converting enzyme 2 OS=Bos taurus GN=ECE2 PE=1 SV=2 |
| TF315192 | evm.model.scaffold_400.196 | 0.25 | 726 | 159 | 2.00E-39 | Neprilysin-11 OS=Caenorhabditis elegans GN=nep-11 PE=1 SV=2 |
| TF315192 | evm.model.scaffold_400.197 | 0.25 | 738 | 160 | 1.00E-39 | Neprilysin-11 OS=Caenorhabditis elegans GN=nep-11 PE=1 SV=2 |
| TF315192 | evm.model.scaffold_400.198 | 0.22 | 530 | 56.2 | 9.00E-07 | Neprilysin-1 OS=Caenorhabditis elegans GN=nep-1 PE=1 SV=3 |
| TF315192 | evm.model.scaffold_400.199  _evm.model.scaffold_400.200 | 0.2 | 365 | 60.5 | 3.00E-08 | Kell blood group glycoprotein OS=Homo sapiens GN=KEL PE=1 SV=2 |
| TF315192 | evm.model.scaffold_447.1 | 0.22 | 263 | 50.8 | 4.00E-05 | MMEL1; membrane metallo-endopeptidase-like 1; K08635 membrane metallo-endopeptidase-like 1 (A) |
| TF315192 | evm.model.scaffold_447.2 | NA | NA | NA | NA | No blast hit |
| TF315192 | evm.model.scaffold_447.3 | 0.24 | 183 | 58.2 | 2.00E-07 | Kell blood group glycoprotein OS=Homo sapiens GN=KEL PE=1 SV=2 |
| TF315192 | evm.model.scaffold_509.44 | 0.24 | 148 | 80.1 | 8.00E-17 | Neprilysin OS=Rattus norvegicus GN=Mme PE=1 SV=2 |
| TF315192 | evm.model.scaffold_509.45 | 0.27 | 423 | 172 | 6.00E-46 | Phosphate-regulating neutral endopeptidase OS=Homo sapiens GN=PHEX PE=1 SV=1 |
| TF315192 | evm.model.scaffold_544.16 | 0.46 | 145 | 136 | 6.00E-37 | Neprilysin OS=Oryctolagus cuniculus GN=MME PE=1 SV=2 |
| TF315192 | evm.model.scaffold_544.17 | 0.37 | 720 | 515 | 1.00E-171 | Membrane metallo-endopeptidase-like 1 OS=Mus musculus GN=Mmel1 PE=1 SV=1 |
| TF315192 | evm.model.scaffold_765.1 | 0.26 | 126 | 47.8 | 2.00E-05 | Membrane metallo-endopeptidase-like 1 OS=Mus musculus GN=Mmel1 PE=1 SV=1 |
| TF315192 | evm.model.scaffold_79.10 | 0.35 | 108 | 65.1 | 2.00E-09 | Endothelin-converting enzyme 1 OS=Mus musculus GN=Ece1 PE=1 SV=1 |
| TF315192 | evm.model.scaffold_89.65 | 0.43 | 695 | 611 | 0 | Membrane metallo-endopeptidase-like 1 OS=Rattus norvegicus GN=Mmel1 PE=1 SV=1 |
| TF315192 | evm.model.scaffold_890.181 | 0.34 | 355 | 240 | 3.00E-70 | Neprilysin-11 OS=Caenorhabditis elegans GN=nep-11 PE=1 SV=2 |
| TF315192 | evm.model.scaffold_890.182 | 0.26 | 204 | 74.7 | 1.00E-13 | Neprilysin-1 OS=Trittame loki PE=1 SV=1 |
| TF315192 | evm.model.scaffold_9.44 | 0.23 | 699 | 185 | 4.00E-48 | Endothelin-converting enzyme 1 OS=Rattus norvegicus GN=Ece1 PE=1 SV=2 |
| TF315192 | evm.model.scaffold_916.54 | 0.21 | 624 | 64.3 | 5.00E-07 | hypothetical protein FF38_14337 [Lucilia cuprina] |
| TF315192 | evm.model.scaffold_94.51 | 0.24 | 647 | 196 | 4.00E-52 | Neprilysin-11 OS=Caenorhabditis elegans GN=nep-11 PE=1 SV=2 |
| TF315192 | evm.model.scaffold_96.115 | 0.29 | 762 | 315 | 1.00E-94 | Neprilysin OS=Rattus norvegicus GN=Mme PE=1 SV=2 |
| TF316846 | evm.model.scaffold_116.53 | 0.34 | 970 | 642 | 0 | Down syndrome cell adhesion molecule-like protein Dscam2 OS=Drosophila melanogaster GN=Dscam2 PE=2 SV=3 |
| TF316846 | evm.model.scaffold_116.54 | 0.36 | 96 | 55.1 | 2.00E-08 | Down syndrome cell adhesion molecule-like protein 1 OS=Homo sapiens GN=DSCAML1 PE=1 SV=2 |
| TF316846 | evm.model.scaffold_116.55 | 0.35 | 71 | 47.8 | 2.00E-06 | Down syndrome cell adhesion molecule homolog OS=Mus musculus GN=Dscam PE=1 SV=1 |
| TF316846 | evm.model.scaffold_116.56 | 0.41 | 85 | 60.8 | 2.00E-10 | Down syndrome cell adhesion molecule-like protein 1 OS=Homo sapiens GN=DSCAML1 PE=1 SV=2 |
| TF316846 | evm.model.scaffold_116.57 | 0.35 | 96 | 59.3 | 2.00E-10 | Down syndrome cell adhesion molecule-like protein 1 OS=Homo sapiens GN=DSCAML1 PE=1 SV=2 |
| TF316846 | evm.model.scaffold_116.58 | 0.3 | 168 | 66.6 | 7.00E-12 | Myopalladin OS=Mus musculus GN=Mypn PE=2 SV=2 |
| TF316846 | evm.model.scaffold_116.59 | 0.44 | 835 | 717 | 0 | Vacuolar protein sorting-associated protein 54 OS=Drosophila melanogaster GN=scat PE=1 SV=1 |
| TF316846 | evm.model.scaffold_359.24 | 0.41 | 1401 | 1058 | 0 | Down syndrome cell adhesion molecule-like protein Dscam2 OS=Drosophila melanogaster GN=Dscam2 PE=2 SV=3 |
| TF316846 | evm.model.scaffold_359.25 | 0.28 | 257 | 96.7 | 9.00E-20 | Down syndrome cell adhesion molecule-like protein Dscam2 OS=Drosophila melanogaster GN=Dscam2 PE=2 SV=3 |
| TF316846 | evm.model.scaffold_37.139 | 0.37 | 1096 | 696 | 0 | Down syndrome cell adhesion molecule-like protein Dscam2 OS=Drosophila melanogaster GN=Dscam2 PE=2 SV=3 |
| TF316846 | evm.model.scaffold_37.140 | 0.33 | 574 | 310 | 1.00E-89 | Down syndrome cell adhesion molecule-like protein Dscam2 OS=Drosophila melanogaster GN=Dscam2 PE=2 SV=3 |
| TF316846 | evm.model.scaffold_9.45 | 0.55 | 1887 | 2037 | 0 | Down syndrome cell adhesion molecule-like protein Dscam2 OS=Drosophila melanogaster GN=Dscam2 PE=2 SV=3 |
| TF316871 | evm.model.scaffold_46.23 | 0.25 | 671 | 238 | 4.00E-66 | Otoferlin OS=Danio rerio GN=otof PE=3 SV=1 |
| TF316871 | evm.model.scaffold_46.24 | 0.41 | 148 | 112 | 6.00E-28 | Otoferlin OS=Mus musculus GN=Otof PE=1 SV=1 |
| TF316871 | evm.model.scaffold_46.25 | 0.61 | 99 | 129 | 1.00E-34 | Otoferlin OS=Danio rerio GN=otof PE=3 SV=1 |
| TF316871 | evm.model.scaffold_46.26 | 0.49 | 135 | 130 | 9.00E-34 | Otoferlin OS=Danio rerio GN=otof PE=3 SV=1 |
| TF316871 | evm.model.scaffold_739.15 | 0.51 | 2016 | 2043 | 0 | Otoferlin OS=Danio rerio GN=otof PE=3 SV=1 |
| TF316871 | evm.model.scaffold_913.2 | 0.42 | 157 | 134 | 7.00E-36 | Otoferlin OS=Homo sapiens GN=OTOF PE=1 SV=3 |
| TF316871 | evm.model.scaffold_913.3 | 0.27 | 657 | 299 | 2.00E-86 | Otoferlin OS=Homo sapiens GN=OTOF PE=1 SV=3 |
| TF316871 | evm.model.scaffold_913.4 | 0.41 | 134 | 96.3 | 7.00E-23 | Fer-1-like protein 6 OS=Homo sapiens GN=FER1L6 PE=2 SV=2 |
| TF316871 | evm.model.scaffold_913.5 | 0.57 | 284 | 332 | 1.00E-101 | Otoferlin OS=Danio rerio GN=otof PE=3 SV=1 |
| TF317925 | evm.model.scaffold_103.31 | 0.35 | 454 | 338 | 1.00E-110 | Protein croquemort OS=Drosophila melanogaster GN=crq PE=1 SV=2 |
| TF317925 | evm.model.scaffold_107.1 | 0.34 | 168 | 117 | 2.00E-29 | Protein croquemort OS=Drosophila melanogaster GN=crq PE=1 SV=2 |
| TF317925 | evm.model.scaffold_107.2 | 0.36 | 219 | 176 | 2.00E-51 | Protein croquemort OS=Drosophila melanogaster GN=crq PE=1 SV=2 |
| TF317925 | evm.model.scaffold_11.68.1 | 0.31 | 422 | 221 | 3.00E-64 | Scavenger receptor class B member 1 OS=Bos taurus GN=SCARB1 PE=2 SV=1 |
| TF317925 | evm.model.scaffold_11.73 | 0.41 | 92 | 81.3 | 2.00E-17 | Scavenger receptor class B member 1 OS=Cricetulus griseus GN=SCARB1 PE=2 SV=1 |
| TF317925 | evm.model.scaffold_153.1 | 0.27 | 180 | 68.2 | 1.00E-12 | Scavenger receptor class B member 1 OS=Homo sapiens GN=SCARB1 PE=1 SV=1 |
| TF317925 | evm.model.scaffold_153.2 | 0.43 | 74 | 68.2 | 4.00E-13 | Scavenger receptor class B member 1 OS=Cricetulus griseus GN=SCARB1 PE=2 SV=1 |
| TF317925 | evm.model.scaffold_283.18 | 0.31 | 423 | 197 | 3.00E-56 | Sensory neuron membrane protein 2 OS=Heliothis virescens GN=snmp2 PE=2 SV=1 |
| TF317925 | evm.model.scaffold_283.19 | 0.32 | 546 | 284 | 2.00E-88 | Sensory neuron membrane protein 2 OS=Drosophila melanogaster GN=Snmp2 PE=2 SV=1 |
| TF317925 | evm.model.scaffold_285.12 | 0.24 | 449 | 131 | 5.00E-32 | Lysosome membrane protein 2 OS=Rattus norvegicus GN=Scarb2 PE=1 SV=2 |
| TF317925 | evm.model.scaffold_315.2 | 0.36 | 372 | 285 | 1.00E-90 | Protein croquemort OS=Drosophila melanogaster GN=crq PE=1 SV=2 |
| TF317925 | evm.model.scaffold_428.173 | 0.33 | 435 | 251 | 9.00E-77 | Scavenger receptor class B member 1 OS=Sus scrofa GN=SCARB1 PE=2 SV=1 |
| TF317925 | evm.model.scaffold_428.176 | 0.25 | 162 | 58.2 | 3.00E-09 | Sensory neuron membrane protein 2 OS=Heliothis virescens GN=snmp2 PE=2 SV=1 |
| TF317925 | evm.model.scaffold_428.177 | 0.31 | 378 | 209 | 3.00E-60 | Scavenger receptor class B member 1 OS=Mus musculus GN=Scarb1 PE=1 SV=1 |
| TF317925 | evm.model.scaffold_53.5 | 0.33 | 460 | 270 | 5.00E-82 | Scavenger receptor class B member 1 OS=Sus scrofa GN=SCARB1 PE=2 SV=1 |
| TF317925 | evm.model.scaffold_53.6 | 0.24 | 406 | 107 | 1.00E-23 | Scavenger receptor class B member 1 OS=Homo sapiens GN=SCARB1 PE=1 SV=1 |
| TF317925 | evm.model.scaffold_53.7.1 | 0.31 | 338 | 194 | 8.00E-55 | Scavenger receptor class B member 1 OS=Mus musculus GN=Scarb1 PE=1 SV=1 |
| TF317925 | evm.model.scaffold_58.1 | 0.58 | 407 | 492 | 1.00E-171 | Sensory neuron membrane protein 1 OS=Tribolium castaneum GN=SNMP01 PE=3 SV=1 |
| TF317925 | evm.model.scaffold_58.2 | 0.44 | 294 | 256 | 2.00E-79 | Sensory neuron membrane protein 1 OS=Tribolium castaneum GN=SNMP01 PE=3 SV=1 |
| TF317925 | evm.model.scaffold_58.3 | 0.5 | 82 | 96.7 | 4.00E-23 | Sensory neuron membrane protein 1 OS=Tribolium castaneum GN=SNMP01 PE=3 SV=1 |
| TF317925 | evm.model.scaffold_655.1 | 0.29 | 147 | 83.6 | 1.00E-17 | Scavenger receptor class B member 1 OS=Bos taurus GN=SCARB1 PE=2 SV=1 |
| TF317925 | evm.model.scaffold_85.179 | 0.32 | 392 | 241 | 5.00E-74 | Protein croquemort OS=Drosophila melanogaster GN=crq PE=1 SV=2 |
| TF317925 | evm.model.scaffold_85.180 | 0.31 | 382 | 229 | 7.00E-70 | Protein croquemort OS=Drosophila melanogaster GN=crq PE=1 SV=2 |
| TF317925 | evm.model.scaffold_85.319 | 0.29 | 474 | 192 | 2.00E-53 | Scavenger receptor class B member 1 OS=Homo sapiens GN=SCARB1 PE=1 SV=1 |
| TF317925 | evm.model.scaffold_89.59 | 0.31 | 408 | 233 | 9.00E-69 | Scavenger receptor class B member 1 OS=Bos taurus GN=SCARB1 PE=2 SV=1 |
| TF317925 | evm.model.scaffold_94.203 | 0.29 | 474 | 192 | 2.00E-53 | Scavenger receptor class B member 1 OS=Homo sapiens GN=SCARB1 PE=1 SV=1 |
| TF317925 | evm.model.scaffold_96.14 | 0.43 | 480 | 407 | 1.00E-136 | Sensory neuron membrane protein 1 OS=Tribolium castaneum GN=SNMP01 PE=3 SV=1 |
| TF317925 | evm.model.scaffold_96.15 | 0.62 | 325 | 443 | 1.00E-153 | Sensory neuron membrane protein 1 OS=Tribolium castaneum GN=SNMP01 PE=3 SV=1 |
| TF318348 | evm.model.scaffold_122.5 | 0.25 | 477 | 127 | 1.00E-30 | Spermine oxidase OS=Mus musculus GN=Smox PE=1 SV=1 |
| TF318348 | evm.model.scaffold_122.6 | 0.32 | 550 | 213 | 2.00E-61 | Spermine oxidase OS=Mus musculus GN=Smox PE=1 SV=1 |
| TF318348 | evm.model.scaffold_14.23 | 0.29 | 512 | 145 | 3.00E-37 | Peroxisomal N(1)-acetyl-spermine/spermidine oxidase OS=Mus musculus GN=Paox PE=1 SV=3 |
| TF318348 | evm.model.scaffold_14.35 | 0.25 | 558 | 142 | 1.00E-35 | Probable polyamine oxidase 5 OS=Arabidopsis thaliana GN=PAO5 PE=2 SV=1 |
| TF318348 | evm.model.scaffold_268.2 | 0.31 | 547 | 209 | 4.00E-57 | Spermine oxidase OS=Mus musculus GN=Smox PE=1 SV=1 |
| TF318348 | evm.model.scaffold_470.1 | 0.25 | 455 | 106 | 8.00E-23 | Peroxisomal N(1)-acetyl-spermine/spermidine oxidase OS=Mus musculus GN=Paox PE=1 SV=3 |
| TF318348 | evm.model.scaffold_515.1 | 0.31 | 476 | 227 | 2.00E-67 | Spermine oxidase OS=Homo sapiens GN=SMOX PE=1 SV=1 |
| TF318348 | evm.model.scaffold_59.146 | 0.3 | 545 | 202 | 3.00E-57 | Spermine oxidase OS=Mus musculus GN=Smox PE=1 SV=1 |
| TF318348 | evm.model.scaffold_604.2 | 0.33 | 530 | 256 | 2.00E-77 | Spermine oxidase OS=Homo sapiens GN=SMOX PE=1 SV=1 |
| TF318348 | evm.model.scaffold_94.194 | 0.35 | 525 | 278 | 1.00E-85 | Spermine oxidase OS=Mus musculus GN=Smox PE=1 SV=1 |
| TF323897 | evm.model.scaffold_890.132 | 0.27 | 177 | 71.6 | 1.00E-12 | Cilia- and flagella-associated protein 61 OS=Homo sapiens GN=CFAP61 PE=2 SV=3 |
| TF323897 | evm.model.scaffold_890.133 | 0.29 | 118 | 57.8 | 1.00E-07 | hypothetical protein; K06756 neuronal cell adhesion molecule (A) |
| TF323897 | evm.model.scaffold_890.134 | 0.96 | 74 | 149 | 4.00E-39 | hypothetical protein D910_07211, partial [Dendroctonus ponderosae] |
| TF323897 | evm.model.scaffold_890.135 | 0.93 | 74 | 149 | 1.00E-39 | hypothetical protein D910_07210 [Dendroctonus ponderosae] |
| TF323897 | evm.model.scaffold_890.136 | 0.31 | 198 | 119 | 2.00E-29 | Cilia- and flagella-associated protein 61 OS=Mus musculus GN=Cfap61 PE=2 SV=2 |
| TF323897 | evm.model.scaffold_890.137 | 0.37 | 170 | 112 | 7.00E-28 | Cilia- and flagella-associated protein 61 OS=Homo sapiens GN=CFAP61 PE=2 SV=3 |
| TF324243 | evm.model.scaffold_662.2 | 0.45 | 103 | 88.2 | 2.00E-20 | Exocyst complex component 7 OS=Drosophila melanogaster GN=exo70 PE=1 SV=2 |
| TF324243 | evm.model.scaffold_662.3 | 0.39 | 601 | 424 | 1.00E-139 | Exocyst complex component 7 OS=Drosophila melanogaster GN=exo70 PE=1 SV=2 |
| TF324243 | evm.model.scaffold_731.3 | 0.53 | 66 | 70.1 | 1.00E-14 | Exocyst complex component 7 OS=Drosophila melanogaster GN=exo70 PE=1 SV=2 |
| TF324243 | evm.model.scaffold_731.4 | 0.37 | 620 | 392 | 1.00E-127 | Exocyst complex component 7 OS=Drosophila melanogaster GN=exo70 PE=1 SV=2 |
| TF324243 | evm.model.scaffold_74.12 | 0.41 | 696 | 524 | 1.00E-177 | Exocyst complex component 7 OS=Drosophila melanogaster GN=exo70 PE=1 SV=2 |
| TF324243 | evm.model.scaffold_74.4 | 0.37 | 620 | 392 | 1.00E-127 | Exocyst complex component 7 OS=Drosophila melanogaster GN=exo70 PE=1 SV=2 |
| TF324243 | evm.model.scaffold_74.5 | 0.53 | 66 | 70.1 | 1.00E-14 | Exocyst complex component 7 OS=Drosophila melanogaster GN=exo70 PE=1 SV=2 |
| TF324981 | evm.model.scaffold_28.3 | 0.31 | 183 | 95.9 | 3.00E-20 | Apolipophorins OS=Locusta migratoria PE=1 SV=2 |
| TF324981 | evm.model.scaffold_28.4 | 0.3 | 128 | 49.3 | 1.00E-05 | Apolipophorins OS=Manduca sexta PE=2 SV=1 |
| TF324981 | evm.model.scaffold_28.5 | 0.32 | 132 | 63.2 | 5.00E-11 | Apolipophorins OS=Manduca sexta PE=2 SV=1 |
| TF324981 | evm.model.scaffold_28.6 | 0.32 | 120 | 65.5 | 3.00E-10 | Apolipophorins OS=Manduca sexta PE=2 SV=1 |
| TF324981 | evm.model.scaffold_28.7 | 0.34 | 525 | 313 | 4.00E-89 | Apolipophorins OS=Manduca sexta PE=2 SV=1 |
| TF324981 | evm.model.scaffold_71.59 | 0.29 | 3483 | 1311 | 0 | Apolipophorins OS=Locusta migratoria PE=1 SV=2 |
| TF324981 | evm.model.scaffold_776.302 | 0.25 | 1081 | 291 | 1.00E-77 | Apolipophorins OS=Locusta migratoria PE=1 SV=2 |
| TF325169 | evm.model.scaffold_115.18 | 0.31 | 133 | 47.4 | 5.00E-05 | Nephrin OS=Homo sapiens GN=NPHS1 PE=1 SV=1 |
| TF325169 | evm.model.scaffold_117.12 | 0.24 | 493 | 96.7 | 4.00E-19 | Protein turtle homolog A OS=Mus musculus GN=Igsf9 PE=1 SV=2 |
| TF325169 | evm.model.scaffold_156.10 | 0.31 | 174 | 63.9 | 2.00E-09 | Nephrin OS=Rattus norvegicus GN=Nphs1 PE=1 SV=2 |
| TF325169 | evm.model.scaffold_359.72 | 0.23 | 425 | 98.6 | 9.00E-20 | Nephrin OS=Mus musculus GN=Nphs1 PE=1 SV=2 |
| TF325169 | evm.model.scaffold_447.58 | 0.27 | 115 | 58.9 | 7.00E-09 | GM14610 gene product from transcript GM14610-RA; K17233 dual oxidase maturation factor 1 (A) |
| TF325169 | evm.model.scaffold_447.62 | 0.70 | 135 | 198 | 7.00E-55 | hypothetical protein TcasGA2_TC008643 [Tribolium castaneum] |
| TF325169 | evm.model.scaffold_447.63 | 0.28 | 202 | 62.4 | 2.00E-09 | Nephrin OS=Homo sapiens GN=NPHS1 PE=1 SV=1 |
| TF325169 | evm.model.scaffold_609.1 | 0.25 | 261 | 75.9 | 9.00E-13 | Nephrin OS=Rattus norvegicus GN=Nphs1 PE=1 SV=2 |
| TF325169 | evm.model.scaffold_609.3 | 0.99 | 226 | 456 | 6.00E-154 | hypothetical protein D910_02981, partial [Dendroctonus ponderosae] |
| TF325169 | evm.model.scaffold_609.4 | 0.22 | 488 | 93.6 | 3.00E-18 | Nephrin OS=Rattus norvegicus GN=Nphs1 PE=1 SV=2 |
| TF325169 | evm.model.scaffold_609.8 | 0.23 | 408 | 89.4 | 1.00E-16 | Nephrin OS=Rattus norvegicus GN=Nphs1 PE=1 SV=2 |
| TF325169 | evm.model.scaffold_62.174 | 0.28 | 260 | 79.7 | 3.00E-14 | Nephrin OS=Homo sapiens GN=NPHS1 PE=1 SV=1 |
| TF325169 | evm.model.scaffold_62.31 | 0.37 | 82 | 54.7 | 3.00E-07 | hypothetical protein Phum_PHUM109400 [Pediculus humanus corporis] |
| TF325169 | evm.model.scaffold_62.32 | 0.26 | 236 | 63.2 | 4.00E-09 | Neural cell adhesion molecule 2 OS=Homo sapiens GN=NCAM2 PE=1 SV=2 |
| TF325169 | evm.model.scaffold_62.33 | 0.26 | 182 | 61.2 | 1.00E-09 | Nephrin OS=Mus musculus GN=Nphs1 PE=1 SV=2 |
| TF325169 | evm.model.scaffold_62.35 | 0.48 | 110 | 103 | 2.00E-24 | GM14610 gene product from transcript GM14610-RA; K17233 dual oxidase maturation factor 1 (A) |
| TF325169 | evm.model.scaffold_90.21 | 0.22 | 488 | 93.6 | 4.00E-18 | Nephrin OS=Rattus norvegicus GN=Nphs1 PE=1 SV=2 |
| TF325169 | evm.model.scaffold_90.23 | 0.25 | 376 | 80.1 | 3.00E-14 | Titin OS=Homo sapiens GN=TTN PE=1 SV=4 |
| TF325169 | evm.model.scaffold_90.24 | 0.48 | 115 | 109 | 8.00E-26 | GM14610 gene product from transcript GM14610-RA; K17233 dual oxidase maturation factor 1 (A) |
| TF325406 | evm.model.scaffold_259.3 | 0.78 | 259 | 423 | 3.00E-146 | hypothetical protein YQE_12279, partial [Dendroctonus ponderosae] |
| TF325406 | evm.model.scaffold_26.96 | 0.68 | 167 | 229 | 2.00E-71 | hypothetical protein YQE_12279, partial [Dendroctonus ponderosae] |
| TF325406 | evm.model.scaffold_260.2 | 1.00 | 292 | 597 | 0 | hypothetical protein YQE_12279, partial [Dendroctonus ponderosae] |
| TF325406 | evm.model.scaffold_277.1 | 0.94 | 99 | 187 | 4.00E-56 | hypothetical protein YQE_12279, partial [Dendroctonus ponderosae] |
| TF325406 | evm.model.scaffold_277.2 | 0.96 | 142 | 273 | 5.00E-89 | hypothetical protein YQE_12279, partial [Dendroctonus ponderosae] |
| TF325406 | evm.model.scaffold_649.1 | 0.91 | 88 | 149 | 2.00E-41 | hypothetical protein YQE_12279, partial [Dendroctonus ponderosae] |
| TF325406 | evm.model.scaffold_874.4 | 0.78 | 259 | 423 | 3.00E-146 | hypothetical protein YQE_12279, partial [Dendroctonus ponderosae] |
| TF325406 | evm.model.scaffold_874.6 | 0.96 | 142 | 273 | 5.00E-89 | hypothetical protein YQE_12279, partial [Dendroctonus ponderosae] |
| TF325406 | evm.model.scaffold_874.7 | 0.94 | 99 | 187 | 4.00E-56 | hypothetical protein YQE_12279, partial [Dendroctonus ponderosae] |
| TF325833 | evm.model.scaffold_31.56 | 0.28 | 128 | 52 | 4.00E-06 | Sodium channel protein Nach OS=Drosophila ananassae GN=Nach PE=3 SV=1 |
| TF325833 | evm.model.scaffold_322.96 | 0.53 | 43 | 53.1 | 2.00E-08 | sodium channel protein Nach; K03440 amiloride-sensitive sodium channel, other (A) |
| TF325833 | evm.model.scaffold_322.97 | 0.28 | 189 | 62.8 | 2.00E-10 | Pickpocket protein 11 OS=Drosophila melanogaster GN=ppk11 PE=2 SV=2 |
| TF325833 | evm.model.scaffold_588.20 | 0.21 | 344 | 50.4 | 1.00E-05 | Sodium channel protein Nach OS=Drosophila ananassae GN=Nach PE=3 SV=1 |
| TF325833 | evm.model.scaffold_788.7 | 0.22 | 490 | 66.6 | 2.00E-10 | Sodium channel protein Nach OS=Drosophila melanogaster GN=Nach PE=2 SV=2 |
| TF325833 | evm.model.scaffold_85.131 | 0.23 | 486 | 112 | 4.00E-25 | Sodium channel protein Nach OS=Drosophila melanogaster GN=Nach PE=2 SV=2 |
| TF325833 | evm.model.scaffold_85.8.1 | 0.22 | 490 | 66.2 | 3.00E-10 | Sodium channel protein Nach OS=Drosophila melanogaster GN=Nach PE=2 SV=2 |
| TF325857 | evm.model.scaffold_322.89 | 0.21 | 364 | 93.6 | 2.00E-19 | Sodium channel protein Nach OS=Drosophila melanogaster GN=Nach PE=2 SV=2 |
| TF325857 | evm.model.scaffold_322.90 | 0.21 | 491 | 119 | 9.00E-28 | Sodium channel protein Nach OS=Drosophila ananassae GN=Nach PE=3 SV=1 |
| TF325857 | evm.model.scaffold_322.91_evm.model.scaffold_322.92 | 0.27 | 406 | 127 | 6.00E-31 | Sodium channel protein Nach OS=Drosophila ananassae GN=Nach PE=3 SV=1 |
| TF325857 | evm.model.scaffold_322.93 | 0.28 | 164 | 58.5 | 2.00E-09 | Sodium channel protein Nach OS=Drosophila ananassae GN=Nach PE=3 SV=1 |
| TF325857 | evm.model.scaffold_322.98.3 | 0.24 | 506 | 164 | 2.00E-43 | Sodium channel protein Nach OS=Drosophila ananassae GN=Nach PE=3 SV=1 |
| TF325857 | evm.model.scaffold_324.29 | 0.22 | 529 | 87.4 | 5.00E-17 | Pickpocket protein 28 OS=Drosophila melanogaster GN=ppk28 PE=1 SV=2 |
| TF325857 | evm.model.scaffold_48.15 | 0.27 | 465 | 165 | 8.00E-44 | Sodium channel protein Nach OS=Drosophila ananassae GN=Nach PE=3 SV=1 |
| TF326009 | evm.model.scaffold_85.244 | 0.51 | 149 | 162 | 2.00E-47 | hypothetical protein YQE_01580, partial [Dendroctonus ponderosae] |
| TF326009 | evm.model.scaffold_85.245 | 0.76 | 203 | 303 | 2.00E-101 | hypothetical protein D910_02255 [Dendroctonus ponderosae] |
| TF326009 | evm.model.scaffold_85.246 | 0.41 | 197 | 171 | 3.00E-49 | hypothetical protein TcasGA2_TC000551 [Tribolium castaneum] |
| TF326009 | evm.model.scaffold_85.248 | 0.86 | 322 | 562 | 0 | hypothetical protein YQE_01578, partial [Dendroctonus ponderosae] |
| TF326009 | evm.model.scaffold_85.249 | 0.28 | 226 | 117 | 9.00E-28 | PREDICTED: uncharacterized protein LOC101745712 [Bombyx mori] |
| TF326009 | evm.model.scaffold_85.250 | 0.34 | 321 | 166 | 2.00E-44 | hypothetical protein YQE_01578, partial [Dendroctonus ponderosae] |
| TF326009 | evm.model.scaffold_85.253 | 0.44 | 123 | 127 | 8.00E-34 | hypothetical protein TcasGA2_TC001015 [Tribolium castaneum] |
| TF326009 | evm.model.scaffold_908.3 | 0.93 | 197 | 382 | 1.00E-132 | hypothetical protein YQE_02198, partial [Dendroctonus ponderosae] |
| TF326444 | evm.model.scaffold_46.8 | 0.52 | 93 | 101 | 8.00E-25 | Ecdysone-induced protein 78C OS=Drosophila melanogaster GN=Eip78C PE=2 SV=4 |
| TF326444 | evm.model.scaffold_603.3 | 0.69 | 39 | 68.2 | 3.00E-11 | Ecdysone-induced protein 78C OS=Drosophila melanogaster GN=Eip78C PE=2 SV=4 |
| TF326444 | evm.model.scaffold_603.4 | 0.53 | 253 | 285 | 3.00E-88 | Ecdysone-induced protein 78C OS=Drosophila melanogaster GN=Eip78C PE=2 SV=4 |
| TF326444 | evm.model.scaffold_739.1 | 0.94 | 37 | 83.2 | 2.00E-17 | Ecdysone-induced protein 78C OS=Drosophila melanogaster GN=Eip78C PE=2 SV=4 |
| TF326825 | evm.model.scaffold_215.2 | 0.23 | 289 | 54.7 | 3.00E-06 | Neuropilin-2 OS=Rattus norvegicus GN=Nrp2 PE=2 SV=1 |
| TF326825 | evm.model.scaffold_22.33 | 0.42 | 63 | 54.7 | 3.00E-05 | low-density lipoprotein receptor-related protein 2; K06233 low density lipoprotein-related protein 2 (A) |
| TF326825 | evm.model.scaffold_422.1 | 0.46 | 50 | 47 | 9.00E-06 | Low-density lipoprotein receptor-related protein 2 OS=Homo sapiens GN=LRP2 PE=1 SV=3 |
| TF326825 | evm.model.scaffold_422.3 | 0.91 | 789 | 1523 | 0 | hypothetical protein D910_02838 [Dendroctonus ponderosae] |
| TF326825 | evm.model.scaffold_47.32 | 0.24 | 389 | 70.1 | 6.00E-11 | Dorsal-ventral patterning protein tolloid OS=Drosophila melanogaster GN=tld PE=2 SV=2 |
| TF326825 | evm.model.scaffold_622.2 | 0.98 | 856 | 1684 | 0 | hypothetical protein D910_02838 [Dendroctonus ponderosae] |
| TF326825 | evm.model.scaffold_622.4 | 0.43 | 57 | 47.8 | 1.00E-05 | Low-density lipoprotein receptor-related protein 2 OS=Homo sapiens GN=LRP2 PE=1 SV=3 |
| TF326825 | evm.model.scaffold_668.1 | 0.24 | 280 | 56.6 | 8.00E-07 | Neuropilin-2 OS=Rattus norvegicus GN=Nrp2 PE=2 SV=1 |
| TF326825 | evm.model.scaffold_896.1 | 0.76 | 297 | 546 | 0 | hypothetical protein D910_06555 [Dendroctonus ponderosae] |
| TF327635 | evm.model.scaffold_7.33 | 0.98 | 173 | 365 | 2.00E-121 | hypothetical protein D910_12371, partial [Dendroctonus ponderosae] |
| TF327635 | evm.model.scaffold_7.38 | 0.86 | 124 | 244 | 4.00E-75 | hypothetical protein D910_12371, partial [Dendroctonus ponderosae] |
| TF327635 | evm.model.scaffold_7.39 | 0.87 | 217 | 419 | 7.00E-139 | hypothetical protein D910_12371, partial [Dendroctonus ponderosae] |
| TF328680 | evm.model.scaffold_29.37 | 0.36 | 872 | 520 | 1.00E-170 | Cilia- and flagella-associated protein 58 OS=Mus musculus GN=Cfap58 PE=2 SV=1 |
| TF328680 | evm.model.scaffold_422.14 | 0.32 | 149 | 72.8 | 3.00E-14 | Cilia- and flagella-associated protein 58 OS=Mus musculus GN=Cfap58 PE=2 SV=1 |
| TF328680 | evm.model.scaffold_653.14 | 0.33 | 106 | 57 | 7.00E-09 | Cilia- and flagella-associated protein 58 OS=Mus musculus GN=Cfap58 PE=2 SV=1 |
| TF329359 | evm.model.scaffold_10.99 | 0.36 | 250 | 166 | 2.00E-48 | Carbonyl reductase [NADPH] 3 OS=Mus musculus GN=Cbr3 PE=2 SV=1 |
| TF329359 | evm.model.scaffold_42.36 | 0.42 | 259 | 204 | 1.00E-63 | Carbonyl reductase [NADPH] 3 OS=Mus musculus GN=Cbr3 PE=2 SV=1 |
| TF329359 | evm.model.scaffold_54.112 | 0.39 | 264 | 190 | 3.00E-58 | Carbonyl reductase [NADPH] 3 OS=Mus musculus GN=Cbr3 PE=2 SV=1 |
| TF329359 | evm.model.scaffold_54.95 | 0.41 | 268 | 214 | 7.00E-68 | Carbonyl reductase [NADPH] 3 OS=Mus musculus GN=Cbr3 PE=2 SV=1 |
| TF329359 | evm.model.scaffold_573.27 | 0.41 | 275 | 224 | 4.00E-71 | Carbonyl reductase [NADPH] 3 OS=Mus musculus GN=Cbr3 PE=2 SV=1 |
| TF332348 | evm.model.scaffold_24.222 | 0.43 | 55 | 57.4 | 2.00E-07 | Rap1 Myb domain containing protein; K11113 telomeric repeat-binding factor 2-interacting protein 1 (A) |
| TF332348 | evm.model.scaffold_51.146 | 0.4 | 60 | 48.9 | 2.00E-05 | Telomeric repeat-binding factor 2-interacting protein 1 OS=Danio rerio GN=terf2ip PE=2 SV=2 |
| TF332348 | evm.model.scaffold_780.4 | 0.43 | 55 | 56.6 | 3.00E-07 | Rap1 Myb domain containing protein; K11113 telomeric repeat-binding factor 2-interacting protein 1 (A) |
| TF332348 | evm.model.scaffold_780.7 | 0.43 | 55 | 57.4 | 2.00E-07 | Rap1 Myb domain containing protein; K11113 telomeric repeat-binding factor 2-interacting protein 1 (A) |
| TF332360 | evm.model.scaffold_656.10 | 0.29 | 315 | 137 | 3.00E-32 | Pleckstrin homology domain-containing family G member 4B OS=Homo sapiens GN=PLEKHG4B PE=2 SV=4 |
| TF332360 | evm.model.scaffold_656.11 | 0.93 | 214 | 422 | 9.00E-148 | hypothetical protein D910_10448 [Dendroctonus ponderosae] |
| TF332360 | evm.model.scaffold_844.1 | 0.36 | 368 | 232 | 3.00E-65 | Intersectin-1 OS=Homo sapiens GN=ITSN1 PE=1 SV=3 |
| TF332360 | evm.model.scaffold_882.53 | 0.29 | 268 | 127 | 9.00E-29 | Pleckstrin homology domain-containing family G member 4B OS=Homo sapiens GN=PLEKHG4B PE=2 SV=4 |
| TF332360 | evm.model.scaffold_882.54 | 0.93 | 214 | 421 | 5.00E-147 | hypothetical protein D910_10448 [Dendroctonus ponderosae] |
| TF350862 | evm.model.scaffold_10.91.2 | 0.67 | 464 | 606 | 0 | hypothetical protein D910_10454 [Dendroctonus ponderosae] |
| TF350862 | evm.model.scaffold_3.21 | 0.74 | 378 | 535 | 0 | hypothetical protein D910_06080 [Dendroctonus ponderosae] |
| TF350862 | evm.model.scaffold_55.30 | 0.92 | 352 | 677 | 0 | hypothetical protein YQE_12769, partial [Dendroctonus ponderosae] |
| TF350862 | evm.model.scaffold_766.4 | 0.86 | 128 | 223 | 2.00E-67 | hypothetical protein YQE_11094, partial [Dendroctonus ponderosae] |
| TF350862 | evm.model.scaffold_94.29 | 0.8 | 128 | 213 | 1.00E-62 | hypothetical protein D910_04644 [Dendroctonus ponderosae] |
| TF351124 | evm.model.scaffold_24.124.1 | 0.3 | 475 | 168 | 3.00E-42 | Insulin-like growth factor-binding protein complex acid labile subunit OS=Papio hamadryas GN=IGFALS PE=2 SV=1 |
| TF351124 | evm.model.scaffold_32.8 | 0.25 | 1039 | 203 | 2.00E-51 | Chaoptin OS=Drosophila melanogaster GN=chp PE=1 SV=2 |
| TF351124 | evm.model.scaffold_324.12 | 0.32 | 413 | 182 | 8.00E-50 | Leucine-rich repeat-containing protein 15 OS=Mus musculus GN=Lrrc15 PE=2 SV=1 |
| TF351124 | evm.model.scaffold_37.109 | 0.29 | 302 | 92.8 | 2.00E-18 | Carboxypeptidase N subunit 2 OS=Homo sapiens GN=CPN2 PE=1 SV=3 |
| TF351124 | evm.model.scaffold_38.67 | 0.26 | 455 | 100 | 4.00E-20 | Leucine-rich repeats and immunoglobulin-like domains protein 3 OS=Mus musculus GN=Lrig3 PE=1 SV=1 |
| TF351124 | evm.model.scaffold_383.39 | 0.27 | 482 | 119 | 3.00E-27 | Carboxypeptidase N subunit 2 OS=Mus musculus GN=Cpn2 PE=1 SV=2 |
| TF351124 | evm.model.scaffold_383.44 | 0.27 | 518 | 124 | 5.00E-29 | Leucine-rich repeat-containing protein 15 OS=Mus musculus GN=Lrrc15 PE=2 SV=1 |
| TF351124 | evm.model.scaffold_573.7 | 0.26 | 592 | 153 | 6.00E-38 | Insulin-like growth factor-binding protein complex acid labile subunit OS=Papio hamadryas GN=IGFALS PE=2 SV=1 |
| TF351124 | evm.model.scaffold_61.20 | 0.24 | 374 | 95.5 | 3.00E-20 | Leucine-rich repeat-containing protein 15 OS=Mus musculus GN=Lrrc15 PE=2 SV=1 |
| TF351124 | evm.model.scaffold_64.14 | 0.25 | 308 | 73.6 | 9.00E-13 | Decorin OS=Rattus norvegicus GN=Dcn PE=1 SV=1 |
| TF351124 | evm.model.scaffold_64.15 | 0.28 | 253 | 79.3 | 2.00E-14 | Carboxypeptidase N subunit 2 OS=Homo sapiens GN=CPN2 PE=1 SV=3 |
| TF351124 | evm.model.scaffold_64.16 | 0.28 | 253 | 81.6 | 6.00E-15 | Carboxypeptidase N subunit 2 OS=Homo sapiens GN=CPN2 PE=1 SV=3 |
| TF351124 | evm.model.scaffold_64.17 | 0.31 | 212 | 79.3 | 4.00E-14 | Chaoptin (Fragment) OS=Tribolium castaneum GN=CHP PE=2 SV=1 |
| TF351124 | evm.model.scaffold_64.18 | 0.27 | 297 | 79.7 | 8.00E-15 | Chaoptin (Fragment) OS=Tribolium castaneum GN=CHP PE=2 SV=1 |
| TF351124 | evm.model.scaffold_656.55 | 0.31 | 439 | 176 | 3.00E-47 | Leucine-rich repeat-containing protein 15 OS=Mus musculus GN=Lrrc15 PE=2 SV=1 |
| TF351124 | evm.model.scaffold_68.8.1 | 0.47 | 1304 | 1161 | 0 | Chaoptin OS=Drosophila melanogaster GN=chp PE=1 SV=2 |
| TF351124 | evm.model.scaffold_75.6 | 0.34 | 163 | 79 | 2.00E-15 | Leucine-rich repeat-containing protein 70 OS=Homo sapiens GN=LRRC70 PE=2 SV=1 |
| TF351124 | evm.model.scaffold_776.200 | 0.27 | 617 | 119 | 1.00E-26 | Insulin-like growth factor-binding protein complex acid labile subunit OS=Papio hamadryas GN=IGFALS PE=2 SV=1 |
| TF351124 | evm.model.scaffold_829.9 | 0.28 | 322 | 97.4 | 4.00E-21 | Chondroadherin OS=Rattus norvegicus GN=Chad PE=2 SV=1 |
| TF351124 | evm.model.scaffold_890.169 | 0.35 | 280 | 129 | 7.00E-31 | Leucine-rich repeat neuronal protein 2 OS=Homo sapiens GN=LRRN2 PE=2 SV=2 |
| TF351124 | evm.model.scaffold_95.18 | 0.3 | 340 | 128 | 2.00E-30 | Insulin-like growth factor-binding protein complex acid labile subunit OS=Rattus norvegicus GN=Igfals PE=1 SV=1 |
| TF351124 | evm.model.scaffold_99.55 | 0.27 | 451 | 108 | 3.00E-23 | Insulin-like growth factor-binding protein complex acid labile subunit OS=Mus musculus GN=Igfals PE=2 SV=1 |
| TF352550 | evm.model.scaffold_749.7 | 0.44 | 36 | 43.1 | 6.00E-06 | Leech-derived tryptase inhibitor C OS=Hirudo medicinalis PE=1 SV=1 |
| TF352550 | evm.model.scaffold_85.3 | 0.48 | 47 | 47 | 3.00E-07 | Double-headed protease inhibitor, submandibular gland OS=Vulpes vulpes PE=1 SV=1 |
| TF352550 | evm.model.scaffold_85.5 | 0.48 | 47 | 47 | 3.00E-07 | Double-headed protease inhibitor, submandibular gland OS=Vulpes vulpes PE=1 SV=1 |
| TF352550 | evm.model.scaffold_85.7 | 0.48 | 47 | 45.1 | 2.00E-06 | Double-headed protease inhibitor, submandibular gland OS=Vulpes vulpes PE=1 SV=1 |
| TF352881 | evm.model.scaffold_32.29 | 0.43 | 122 | 99.8 | 3.00E-25 | Heat shock protein 67B3 OS=Drosophila melanogaster GN=Hsp67Bc PE=2 SV=2 |
| TF352881 | evm.model.scaffold_32.68 | 0.48 | 108 | 98.6 | 9.00E-25 | Heat shock protein 67B3 OS=Drosophila melanogaster GN=Hsp67Bc PE=2 SV=2 |
| TF352881 | evm.model.scaffold_390.100 | 0.41 | 116 | 85.5 | 9.00E-21 | Protein lethal(2)essential for life OS=Drosophila melanogaster GN=l(2)efl PE=1 SV=1 |
| TF352881 | evm.model.scaffold_390.101 | 0.5 | 155 | 141 | 1.00E-41 | Protein lethal(2)essential for life OS=Drosophila melanogaster GN=l(2)efl PE=1 SV=1 |
| TF352881 | evm.model.scaffold_390.102 | 0.38 | 158 | 107 | 1.00E-28 | Heat shock protein 23 OS=Drosophila melanogaster GN=Hsp23 PE=2 SV=2 |
| TF352881 | evm.model.scaffold_390.103 | 0.47 | 187 | 151 | 1.00E-45 | Protein lethal(2)essential for life OS=Drosophila melanogaster GN=l(2)efl PE=1 SV=1 |
| TF352881 | evm.model.scaffold_390.104 | 0.45 | 120 | 98.6 | 2.00E-25 | Alpha-crystallin B chain OS=Gallus gallus GN=CRYAB PE=2 SV=2 |
| TF352881 | evm.model.scaffold_390.98 | 0.46 | 184 | 152 | 7.00E-46 | Protein lethal(2)essential for life OS=Drosophila melanogaster GN=l(2)efl PE=1 SV=1 |
| TF352881 | evm.model.scaffold_390.99 | 0.38 | 168 | 99.8 | 3.00E-25 | Heat shock protein 26 OS=Drosophila melanogaster GN=Hsp26 PE=1 SV=2 |
| TF352881 | evm.model.scaffold_447.34.1 | 0.52 | 201 | 199 | 9.00E-64 | Protein lethal(2)essential for life OS=Drosophila melanogaster GN=l(2)efl PE=1 SV=1 |
| TF352881 | evm.model.scaffold_561.88 | 0.49 | 101 | 100 | 6.00E-26 | Alpha-crystallin B chain OS=Gallus gallus GN=CRYAB PE=2 SV=2 |
| TF352881 | evm.model.scaffold_561.89 | 0.47 | 187 | 151 | 1.00E-45 | Protein lethal(2)essential for life OS=Drosophila melanogaster GN=l(2)efl PE=1 SV=1 |
| TF352881 | evm.model.scaffold_561.90 | 0.39 | 161 | 108 | 1.00E-28 | Heat shock protein 23 OS=Drosophila melanogaster GN=Hsp23 PE=2 SV=2 |
| TF352881 | evm.model.scaffold_561.91 | 0.5 | 155 | 140 | 1.00E-41 | Protein lethal(2)essential for life OS=Drosophila melanogaster GN=l(2)efl PE=1 SV=1 |
| TF352881 | evm.model.scaffold_561.92 | 0.41 | 153 | 110 | 6.00E-30 | Protein lethal(2)essential for life OS=Drosophila melanogaster GN=l(2)efl PE=1 SV=1 |
| TF352881 | evm.model.scaffold_561.93.1 | 0.4 | 137 | 99 | 6.00E-25 | Heat shock protein 67B3 OS=Drosophila melanogaster GN=Hsp67Bc PE=2 SV=2 |
| TF352881 | evm.model.scaffold_561.94 | 0.46 | 184 | 152 | 7.00E-46 | Protein lethal(2)essential for life OS=Drosophila melanogaster GN=l(2)efl PE=1 SV=1 |
| TF353639 | evm.model.scaffold_115.6 | 0.31 | 280 | 152 | 7.00E-43 | N-acetylneuraminate lyase OS=Pongo abelii GN=NPL PE=2 SV=1 |
| TF353639 | evm.model.scaffold_40.41 | 0.3 | 282 | 139 | 3.00E-36 | N-acetylneuraminate lyase OS=Homo sapiens GN=NPL PE=1 SV=1 |
| TF353639 | evm.model.scaffold_447.77 | 0.31 | 280 | 152 | 7.00E-43 | N-acetylneuraminate lyase OS=Pongo abelii GN=NPL PE=2 SV=1 |
| TF353639 | evm.model.scaffold_615.29 | 0.33 | 280 | 172 | 2.00E-50 | N-acetylneuraminate lyase OS=Mus musculus GN=Npl PE=1 SV=1 |
| TF353639 | evm.model.scaffold_62.161 | 0.28 | 241 | 112 | 6.00E-28 | N-acetylneuraminate lyase OS=Danio rerio GN=npl PE=2 SV=1 |
