# Supplementary material for: Chromosome-level genome assembly and population genomic analyses provide insights into adaptive evolution of the red turpentine beetle, Dendroctonus valens
Source: BMC Biol. 2022 Aug 24;20:190. doi: 10.1186/s12915-022-01388-y (PMC9400205; doi:10.1186/s12915-022-01388-y)
Supplement: Supplementary file 1 — Additional file 1: Table S1. Summary statistics of genome sequencing data of Dendroctonus valens. Table S2. Summary statistics of genome assembly of Dendroctonus valens. Table S3. BUSCO evaluation result for genome assembly of Dendroctonus valens. Table S4. Summary statistics of transposable elements in Dendroctonus valens genome. Table S5. Summary of gene families manually curated in Dendroctonus valens genome. Table S6. Summary statistics of genome annotation in Dendroctonus valens genome. Table S7. List of gene families that are unique in Dendroctonus valens compared to other three Coleoptera species. Table S8. Gene families that are rapidly expanded in Dendroctonus valens revealed by CAFE analysis. Table S9. Gene families that are rapidly contracted in Dendroctonus valens revealed by CAFE analysis. Table S10. List of genes that are positively selected in Dendroctonus valens revealed by codeml analysis. Table S11. Gene ontology enrichment result of positively selected genes in Dendroctonus valens. Table S12. Sampling site information for genome resequencing of geographical populations. Table S13. Summary statistics of genome resequencing data in different populations. Table S14. List of genes that undergo selective sweep in the China population compared to CAMT population. [file 12915_2022_1388_MOESM1_ESM.zip › Table S9.docx]

| **Table S9** Gene families that are rapidly contracted in *Dendroctonus valens* revealed by CAFE analysis | | | | | | |
| --- | --- | --- | --- | --- | --- | --- |
| **TreeFam ID** | **Gene ID** | **Identity** | **Aligned length** | **Score** | **E-value** | **Description** |
| TF105191 | evm.model.scaffold_14.14.1 | 0.29 | 1768 | 617 | 0 | ATP-binding cassette sub-family A member 3 OS=Homo sapiens GN=ABCA3 PE=1 SV=2 |
| TF105191 | evm.model.scaffold_832.67.2 | 0.38 | 1780 | 1198 | 0 | ATP-binding cassette sub-family A member 3 OS=Homo sapiens GN=ABCA3 PE=1 SV=2 |
| TF313309 | evm.model.scaffold_14.64 | 0.32 | 794 | 437 | 1.00E-140 | Inactive dipeptidyl peptidase 10 OS=Homo sapiens GN=DPP10 PE=1 SV=2 |
| TF313309 | evm.model.scaffold_14.65 | 0.32 | 708 | 390 | 1.00E-123 | Inactive dipeptidyl peptidase 10 OS=Homo sapiens GN=DPP10 PE=1 SV=2 |
| TF313309 | evm.model.scaffold_427.14.6 | 0.31 | 827 | 404 | 1.00E-127 | Inactive dipeptidyl peptidase 10 OS=Homo sapiens GN=DPP10 PE=1 SV=2 |
| TF313309 | evm.model.scaffold_427.15 | 0.43 | 731 | 578 | 0 | Venom dipeptidyl peptidase 4 OS=Vespula vulgaris PE=1 SV=1 |
| TF313309 | evm.model.scaffold_54.74 | 0.33 | 846 | 468 | 1.00E-151 | Inactive dipeptidyl peptidase 10 OS=Mus musculus GN=Dpp10 PE=1 SV=1 |
| TF313792 | evm.model.scaffold_197.5 | 0.39 | 248 | 193 | 2.00E-52 | Monocarboxylate transporter 12 OS=Mus musculus GN=Slc16a12 PE=2 SV=1 |
| TF313792 | evm.model.scaffold_20.28 | 0.28 | 243 | 106 | 6.00E-23 | Monocarboxylate transporter 3 OS=Gallus gallus GN=SLC16A3 PE=2 SV=2 |
| TF313792 | evm.model.scaffold_20.29 | 0.25 | 549 | 187 | 6.00E-51 | Monocarboxylate transporter 5 OS=Mus musculus GN=Slc16a4 PE=2 SV=1 |
| TF313792 | evm.model.scaffold_215.30 | 0.28 | 172 | 97.8 | 5.00E-22 | Monocarboxylate transporter 14 OS=Homo sapiens GN=SLC16A14 PE=2 SV=1 |
| TF313792 | evm.model.scaffold_215.86 | 0.35 | 219 | 156 | 8.00E-42 | Monocarboxylate transporter 12 OS=Xenopus tropicalis GN=slc16a12 PE=2 SV=1 |
| TF313792 | evm.model.scaffold_30.36.1 | 0.4 | 443 | 322 | 1.00E-103 | Monocarboxylate transporter 10 OS=Danio rerio GN=slc16a10 PE=2 SV=1 |
| TF313792 | evm.model.scaffold_359.20 | 0.23 | 267 | 63.2 | 1.00E-09 | Monocarboxylate transporter 7 OS=Mus musculus GN=Slc16a6 PE=1 SV=1 |
| TF313792 | evm.model.scaffold_359.21 | 0.22 | 458 | 79 | 3.00E-14 | Monocarboxylate transporter 7 OS=Mus musculus GN=Slc16a6 PE=1 SV=1 |
| TF313792 | evm.model.scaffold_37.344 | 0.34 | 206 | 139 | 4.00E-34 | Monocarboxylate transporter 12 OS=Homo sapiens GN=SLC16A12 PE=1 SV=2 |
| TF313792 | evm.model.scaffold_467.28 | 0.24 | 254 | 59.7 | 5.00E-08 | Monocarboxylate transporter 8 OS=Homo sapiens GN=SLC16A2 PE=1 SV=2 |
| TF313792 | evm.model.scaffold_50.17 | 0.23 | 173 | 53.1 | 4.00E-06 | hypothetical protein; K08190 MFS transporter, MCP family, solute carrier family 16 (monocarboxylic acid transporters), member 14 (A) |
| TF313792 | evm.model.scaffold_524.31 | 0.37 | 211 | 169 | 4.00E-44 | Monocarboxylate transporter 12 OS=Xenopus tropicalis GN=slc16a12 PE=2 SV=1 |
| TF313792 | evm.model.scaffold_524.50 | 0.24 | 394 | 107 | 2.00E-23 | Monocarboxylate transporter 12 OS=Homo sapiens GN=SLC16A12 PE=1 SV=2 |
| TF313792 | evm.model.scaffold_59.147 | 0.3 | 156 | 71.6 | 5.00E-12 | Monocarboxylate transporter 3 OS=Homo sapiens GN=SLC16A8 PE=2 SV=1 |
| TF313792 | evm.model.scaffold_597.1 | 0.31 | 169 | 73.6 | 1.00E-13 | Monocarboxylate transporter 6 OS=Homo sapiens GN=SLC16A5 PE=2 SV=1 |
| TF313792 | evm.model.scaffold_597.2 | 0.35 | 215 | 160 | 1.00E-43 | Monocarboxylate transporter 12 OS=Xenopus tropicalis GN=slc16a12 PE=2 SV=1 |
| TF313792 | evm.model.scaffold_597.3 | 0.36 | 213 | 156 | 3.00E-40 | Monocarboxylate transporter 12 OS=Xenopus tropicalis GN=slc16a12 PE=2 SV=1 |
| TF313792 | evm.model.scaffold_898.32 | 0.27 | 576 | 217 | 9.00E-63 | Monocarboxylate transporter 5 OS=Mus musculus GN=Slc16a4 PE=2 SV=1 |
| TF313911 | evm.model.scaffold_104.19 | 0.34 | 594 | 312 | 6.00E-96 | Glucose dehydrogenase [FAD, quinone] OS=Drosophila pseudoobscura pseudoobscura GN=Gld PE=3 SV=4 |
| TF313911 | evm.model.scaffold_14.75 | 0.4 | 534 | 384 | 1.00E-125 | Glucose dehydrogenase [FAD, quinone] OS=Drosophila melanogaster GN=Gld PE=3 SV=3 |
| TF313911 | evm.model.scaffold_309.10 | 0.33 | 614 | 324 | 1.00E-101 | Glucose dehydrogenase [FAD, quinone] OS=Drosophila pseudoobscura pseudoobscura GN=Gld PE=3 SV=4 |
| TF313911 | evm.model.scaffold_309.11 | 0.39 | 566 | 386 | 1.00E-125 | Glucose dehydrogenase [FAD, quinone] OS=Drosophila pseudoobscura pseudoobscura GN=Gld PE=3 SV=4 |
| TF313911 | evm.model.scaffold_309.12 | 0.4 | 582 | 401 | 1.00E-130 | Glucose dehydrogenase [FAD, quinone] OS=Drosophila pseudoobscura pseudoobscura GN=Gld PE=3 SV=4 |
| TF313911 | evm.model.scaffold_309.6 | 0.43 | 608 | 506 | 1.00E-157 | Glucose dehydrogenase [FAD, quinone] OS=Drosophila pseudoobscura pseudoobscura GN=Gld PE=3 SV=4 |
| TF313911 | evm.model.scaffold_309.8 | 0.35 | 500 | 290 | 4.00E-86 | Glucose dehydrogenase [FAD, quinone] OS=Drosophila pseudoobscura pseudoobscura GN=Gld PE=3 SV=4 |
| TF313911 | evm.model.scaffold_309.9 | 0.51 | 92 | 109 | 5.00E-28 | Glucose dehydrogenase [FAD, quinone] OS=Drosophila melanogaster GN=Gld PE=3 SV=3 |
| TF313911 | evm.model.scaffold_32.22 | 0.35 | 570 | 311 | 1.00E-96 | Glucose dehydrogenase [FAD, quinone] OS=Drosophila pseudoobscura pseudoobscura GN=Gld PE=3 SV=4 |
| TF313911 | evm.model.scaffold_359.73 | 0.45 | 612 | 545 | 0 | Glucose dehydrogenase [FAD, quinone] OS=Drosophila melanogaster GN=Gld PE=3 SV=3 |
| TF313911 | evm.model.scaffold_428.147 | 0.47 | 604 | 585 | 0 | Glucose dehydrogenase [FAD, quinone] OS=Drosophila melanogaster GN=Gld PE=3 SV=3 |
| TF313911 | evm.model.scaffold_471.19 | 0.34 | 563 | 294 | 1.00E-90 | Glucose dehydrogenase [FAD, quinone] OS=Drosophila melanogaster GN=Gld PE=3 SV=3 |
| TF313911 | evm.model.scaffold_471.35 | 0.33 | 556 | 286 | 6.00E-88 | Glucose dehydrogenase [FAD, quinone] OS=Drosophila melanogaster GN=Gld PE=3 SV=3 |
| TF313911 | evm.model.scaffold_53.66 | 0.65 | 605 | 824 | 0 | Glucose dehydrogenase [FAD, quinone] OS=Drosophila pseudoobscura pseudoobscura GN=Gld PE=3 SV=4 |
| TF313911 | evm.model.scaffold_561.20 | 0.41 | 582 | 403 | 1.00E-131 | Glucose dehydrogenase [FAD, quinone] OS=Drosophila pseudoobscura pseudoobscura GN=Gld PE=3 SV=4 |
| TF313911 | evm.model.scaffold_561.21 | 0.37 | 595 | 389 | 1.00E-120 | Glucose dehydrogenase [FAD, quinone] OS=Drosophila pseudoobscura pseudoobscura GN=Gld PE=3 SV=4 |
| TF313911 | evm.model.scaffold_561.24 | 0.42 | 596 | 478 | 1.00E-153 | Glucose dehydrogenase [FAD, quinone] OS=Drosophila pseudoobscura pseudoobscura GN=Gld PE=3 SV=4 |
| TF313911 | evm.model.scaffold_561.25 | 0.45 | 572 | 496 | 1.00E-162 | Glucose dehydrogenase [FAD, quinone] OS=Drosophila melanogaster GN=Gld PE=3 SV=3 |
| TF313911 | evm.model.scaffold_601.11 | 0.48 | 43 | 46.6 | 1.00E-06 | Oxygen-dependent choline dehydrogenase OS=Serratia proteamaculans (strain 568) GN=betA PE=3 SV=1 |
| TF313911 | evm.model.scaffold_700.61 | 0.38 | 128 | 89.7 | 4.00E-18 | Glucose dehydrogenase [FAD, quinone] OS=Drosophila melanogaster GN=Gld PE=3 SV=3 |
| TF313911 | evm.model.scaffold_773.10 | 0.38 | 315 | 209 | 1.00E-60 | Glucose dehydrogenase [FAD, quinone] OS=Drosophila melanogaster GN=Gld PE=3 SV=3 |
| TF313911 | evm.model.scaffold_90.64 | 0.39 | 503 | 352 | 1.00E-114 | Glucose dehydrogenase [FAD, quinone] OS=Drosophila pseudoobscura pseudoobscura GN=Gld PE=3 SV=4 |
| TF313950 | evm.model.scaffold_513.37 | 0.22 | 449 | 128 | 3.00E-31 | Equilibrative nucleoside transporter 3 OS=Homo sapiens GN=SLC29A3 PE=1 SV=3 |
| TF313950 | evm.model.scaffold_554.7 | 0.32 | 153 | 90.1 | 2.00E-18 | Equilibrative nucleoside transporter 1 OS=Mus musculus GN=Slc29a1 PE=1 SV=3 |
| TF313950 | evm.model.scaffold_554.8 | 0.66 | 45 | 72 | 3.00E-14 | NADPH-dependent diflavin oxidoreductase 1; K15014 solute carrier family 29 (equilibrative nucleoside transporter), member 1/2/3 (A) |
| TF313950 | evm.model.scaffold_853.4 | 0.34 | 442 | 253 | 9.00E-78 | Equilibrative nucleoside transporter 3 OS=Bos taurus GN=SLC29A3 PE=2 SV=1 |
| TF313965 | evm.model.scaffold_116.16 | 0.41 | 763 | 622 | 0 | Gamma-aminobutyric acid type B receptor subunit 2 OS=Homo sapiens GN=GABBR2 PE=1 SV=1 |
| TF313965 | evm.model.scaffold_26.35 | 0.31 | 757 | 377 | 1.00E-115 | Gamma-aminobutyric acid type B receptor subunit 2 OS=Homo sapiens GN=GABBR2 PE=1 SV=1 |
| TF313965 | evm.model.scaffold_99.205.1 | 0.48 | 790 | 789 | 0 | Gamma-aminobutyric acid type B receptor subunit 1 OS=Mus musculus GN=Gabbr1 PE=1 SV=1 |
| TF314498 | evm.model.scaffold_31.283 | 0.27 | 330 | 95.9 | 2.00E-19 | 4F2 cell-surface antigen heavy chain OS=Homo sapiens GN=SLC3A2 PE=1 SV=3 |
| TF314498 | evm.model.scaffold_37.82 | 0.57 | 1213 | 1426 | 0 | Isoleucine--tRNA ligase, cytoplasmic OS=Homo sapiens GN=IARS PE=1 SV=2 |
| TF314498 | evm.model.scaffold_55.135 | 0.52 | 465 | 468 | 1.00E-158 | Maltase 2 OS=Drosophila virilis GN=Mal-B2 PE=3 SV=2 |
| TF315600 | evm.model.scaffold_285.17.1 | 0.47 | 381 | 350 | 1.00E-118 | Synaptotagmin-7 OS=Homo sapiens GN=SYT7 PE=1 SV=3 |
| TF315600 | evm.model.scaffold_73.33 | 0.61 | 267 | 318 | 1.00E-104 | Synaptotagmin 1 OS=Drosophila melanogaster GN=Syt1 PE=1 SV=3 |
| TF315600 | evm.model.scaffold_73.35 | 0.79 | 373 | 608 | 0 | Synaptotagmin 1 OS=Drosophila melanogaster GN=Syt1 PE=1 SV=3 |
| TF315600 | evm.model.scaffold_96.100 | 0.37 | 300 | 222 | 1.00E-64 | Synaptotagmin-9 OS=Homo sapiens GN=SYT9 PE=2 SV=1 |
| TF315600 | evm.model.scaffold_96.102 | 0.4 | 306 | 216 | 1.00E-63 | Synaptotagmin-12 OS=Homo sapiens GN=SYT12 PE=1 SV=1 |
| TF315605 | evm.model.scaffold_299.5.1 | 0.69 | 523 | 747 | 0 | Acetylcholine receptor subunit beta-like 2 OS=Drosophila melanogaster GN=nAChRbeta2 PE=2 SV=3 |
| TF315605 | evm.model.scaffold_37.52 | 0.72 | 450 | 611 | 0 | Acetylcholine receptor subunit beta-like 1 OS=Drosophila melanogaster GN=nAChRbeta1 PE=2 SV=1 |
| TF315605 | evm.model.scaffold_37.53 | 0.42 | 85 | 78.6 | 1.00E-16 | Neuronal acetylcholine receptor subunit alpha-7 OS=Gallus gallus GN=CHRNA7 PE=1 SV=1 |
| TF315605 | evm.model.scaffold_57.22.2 | 0.73 | 553 | 801 | 0 | Acetylcholine receptor subunit alpha-like 2 OS=Drosophila melanogaster GN=nAChRalpha2 PE=2 SV=1 |
| TF315605 | evm.model.scaffold_776.224 | 0.55 | 70 | 80.5 | 3.00E-17 | Neuronal acetylcholine receptor subunit alpha-7 OS=Macaca mulatta GN=CHRNA7 PE=2 SV=1 |
| TF315605 | evm.model.scaffold_776.225 | 0.78 | 38 | 58.5 | 4.00E-10 | GL19206 gene product from transcript GL19206-RA; K05312 nicotinic acetylcholine receptor, invertebrate (A) |
| TF315605 | evm.model.scaffold_85.254 | 0.72 | 562 | 782 | 0 | Acetylcholine receptor subunit alpha-like 1 OS=Drosophila melanogaster GN=nAChRalpha1 PE=2 SV=2 |
| TF315605 | evm.model.scaffold_908.38 | 0.26 | 360 | 150 | 6.00E-39 | Acetylcholine receptor subunit alpha-type acr-7 OS=Caenorhabditis elegans GN=acr-7 PE=1 SV=2 |
| TF315605 | evm.model.scaffold_99.178 | 0.86 | 350 | 630 | 0 | Acetylcholine receptor subunit alpha-like OS=Manduca sexta GN=ARA1 PE=2 SV=1 |
| TF316735 | evm.model.scaffold_842.26 | 0.39 | 328 | 228 | 4.00E-69 | Flavin-containing monooxygenase FMO GS-OX-like 1 OS=Arabidopsis thaliana GN=At1g12160 PE=2 SV=1 |
| TF316836 | evm.model.scaffold_21.30_evm.model.scaffold_21.31 | 0.53 | 4685 | 4980 | 0 | Dynein heavy chain 8, axonemal OS=Mus musculus GN=Dnah8 PE=1 SV=2 |
| TF316837 | evm.model.scaffold_35.94 | 0.36 | 4122 | 2692 | 0 | Cytoplasmic dynein 2 heavy chain 1 OS=Tripneustes gratilla GN=DYH1B PE=2 SV=2 |
| TF316838 | evm.model.scaffold_407.11.1 | 0.5 | 932 | 986 | 0 | Dynein heavy chain 7, axonemal OS=Homo sapiens GN=DNAH7 PE=1 SV=2 |
| TF316839 | evm.model.scaffold_447.56 | 0.49 | 2151 | 2046 | 0 | Dynein beta chain, ciliary OS=Tripneustes gratilla PE=1 SV=1 |
| TF316840 | evm.model.scaffold_463.114 | 0.54 | 4737 | 5164 | 0 | Dynein heavy chain 5, axonemal OS=Mus musculus GN=Dnah5 PE=2 SV=2 |
| TF316841 | evm.model.scaffold_55.64 | 0.53 | 4162 | 4323 | 0 | Dynein heavy chain 3, axonemal OS=Mus musculus GN=Dnah3 PE=2 SV=2 |
| TF316842 | evm.model.scaffold_59.132 | 0.54 | 1110 | 1255 | 0 | Dynein heavy chain 1, axonemal OS=Homo sapiens GN=DNAH1 PE=2 SV=4 |
| TF316843 | evm.model.scaffold_85.125 | 0.29 | 3365 | 1467 | 0 | Dynein heavy chain 7, axonemal OS=Rattus norvegicus GN=Dnah7 PE=2 SV=2 |
| TF316844 | evm.model.scaffold_85.148_evm.model.scaffold_85.149 | 0.6 | 1022 | 1252 | 0 | Dynein heavy chain 2, axonemal OS=Mus musculus GN=Dnah2 PE=2 SV=1 |
| TF316845 | evm.model.scaffold_916.43 | 0.58 | 3913 | 4781 | 0 | Dynein heavy chain 7, axonemal OS=Homo sapiens GN=DNAH7 PE=1 SV=2 |
| TF320754 | evm.model.scaffold_59.148 | 0.34 | 455 | 239 | 3.00E-71 | Tektin-4 OS=Bos taurus GN=TEKT4 PE=2 SV=1 |
| TF320754 | evm.model.scaffold_62.7 | 0.35 | 392 | 241 | 3.00E-74 | Tektin-2 OS=Rattus norvegicus GN=Tekt2 PE=2 SV=1 |
| TF321302 | evm.model.scaffold_908.54 | 0.32 | 1677 | 872 | 0 | Neurexin-3 OS=Homo sapiens GN=NRXN3 PE=1 SV=4 |
| TF324201 | evm.model.scaffold_102.59.1 | 0.52 | 338 | 323 | 1.00E-109 | Prostaglandin reductase 1 OS=Bos taurus GN=PTGR1 PE=2 SV=1 |
| TF324201 | evm.model.scaffold_102.60 | 0.5 | 334 | 321 | 1.00E-103 | Prostaglandin reductase 1 OS=Rattus norvegicus GN=Ptgr1 PE=2 SV=3 |
| TF324207 | evm.model.scaffold_22.35 | 0.5 | 389 | 389 | 1.00E-133 | Fringe glycosyltransferase OS=Drosophila melanogaster GN=fng PE=1 SV=1 |
| TF325864 | evm.model.scaffold_104.61 | NA | NA | NA | NA | No blast hit |
| TF325864 | evm.model.scaffold_104.63 | NA | NA | NA | NA | No blast hit |
| TF325864 | evm.model.scaffold_104.64 | NA | NA | NA | NA | No blast hit |
| TF325864 | evm.model.scaffold_104.65 | NA | NA | NA | NA | No blast hit |
| TF325864 | evm.model.scaffold_104.66 | NA | NA | NA | NA | No blast hit |
| TF325864 | evm.model.scaffold_104.67 | NA | NA | NA | NA | No blast hit |
| TF325864 | evm.model.scaffold_104.72 | NA | NA | NA | NA | No blast hit |
| TF325864 | evm.model.scaffold_104.73 | NA | NA | NA | NA | No blast hit |
| TF325864 | evm.model.scaffold_104.74 | NA | NA | NA | NA | No blast hit |
| TF325864 | evm.model.scaffold_104.75 | NA | NA | NA | NA | No blast hit |
| TF325864 | evm.model.scaffold_114.4 | 0.26 | 435 | 113 | 6.00E-25 | hypothetical protein; K04507 calcyclin binding protein (A) |
| TF325864 | evm.model.scaffold_14.44 | 0.26 | 296 | 58.2 | 4.00E-07 | hypothetical protein; K04507 calcyclin binding protein (A) |
| TF325864 | evm.model.scaffold_14.45 | NA | NA | NA | NA | No blast hit |
| TF325864 | evm.model.scaffold_324.37 | NA | NA | NA | NA | No blast hit |
| TF325864 | evm.model.scaffold_35.48 | 0.23 | 352 | 72.8 | 8.00E-12 | hypothetical protein; K04507 calcyclin binding protein (A) |
| TF325864 | evm.model.scaffold_35.58 | 0.32 | 149 | 56.6 | 5.00E-06 | hypothetical protein; K04507 calcyclin binding protein (A) |
| TF325864 | evm.model.scaffold_35.59 | NA | NA | NA | NA | No blast hit |
| TF325864 | evm.model.scaffold_38.44 | NA | NA | NA | NA | No blast hit |
| TF325864 | evm.model.scaffold_38.45 | 0.25 | 271 | 63.9 | 6.00E-09 | hypothetical protein; K04507 calcyclin binding protein (A) |
| TF325864 | evm.model.scaffold_38.48 | 0.23 | 313 | 61.2 | 4.00E-08 | hypothetical protein; K04507 calcyclin binding protein (A) |
| TF325864 | evm.model.scaffold_400.112 | 0.23 | 227 | 58.5 | 3.00E-07 | hypothetical protein; K04507 calcyclin binding protein (A) |
| TF325864 | evm.model.scaffold_463.85 | 0.26 | 232 | 65.1 | 2.00E-09 | hypothetical protein; K04507 calcyclin binding protein (A) |
| TF325864 | evm.model.scaffold_471.135 | 0.23 | 285 | 56.2 | 2.00E-06 | hypothetical protein; K04507 calcyclin binding protein (A) |
| TF325864 | evm.model.scaffold_483.42 | 0.35 | 97 | 61.6 | 3.00E-08 | hypothetical protein; K04507 calcyclin binding protein (A) |
| TF325864 | evm.model.scaffold_513.10 | 0.25 | 225 | 66.2 | 1.00E-09 | hypothetical protein; K04507 calcyclin binding protein (A) |
| TF325864 | evm.model.scaffold_513.11 | 0.27 | 292 | 71.2 | 1.00E-11 | hypothetical protein; K04507 calcyclin binding protein (A) |
| TF325864 | evm.model.scaffold_55.177 | 0.26 | 198 | 57 | 1.00E-06 | hypothetical protein; K04507 calcyclin binding protein (A) |
| TF325864 | evm.model.scaffold_55.42 | 0.24 | 228 | 60.5 | 8.00E-08 | hypothetical protein; K04507 calcyclin binding protein (A) |
| TF325864 | evm.model.scaffold_55.99 | NA | NA | NA | NA | No blast hit |
| TF325864 | evm.model.scaffold_59.52 | NA | NA | NA | NA | No blast hit |
| TF325864 | evm.model.scaffold_608.22 | 0.23 | 355 | 58.2 | 4.00E-07 | hypothetical protein; K04507 calcyclin binding protein (A) |
| TF325864 | evm.model.scaffold_62.37 | 0.28 | 250 | 79.3 | 6.00E-14 | hypothetical protein; K04507 calcyclin binding protein (A) |
| TF325864 | evm.model.scaffold_647.104 | NA | NA | NA | NA | No blast hit |
| TF325864 | evm.model.scaffold_647.105 | 0.19 | 255 | 52 | 9.00E-05 | hypothetical protein; K04507 calcyclin binding protein (A) |
| TF325864 | evm.model.scaffold_647.106 | NA | NA | NA | NA | No blast hit |
| TF325864 | evm.model.scaffold_653.28 | 0.2 | 196 | 52.8 | 2.00E-05 | hypothetical protein; K04507 calcyclin binding protein (A) |
| TF326549 | evm.model.scaffold_752.35 | 0.29 | 387 | 174 | 2.00E-48 | Gustatory receptor 5a for trehalose OS=Drosophila melanogaster GN=Gr5a PE=1 SV=2 |
| TF326549 | evm.model.scaffold_85.158 | 0.44 | 56 | 55.1 | 4.00E-08 | Gustatory receptor for sugar taste 64e OS=Drosophila melanogaster GN=Gr64e PE=2 SV=2 |
| TF326549 | evm.model.scaffold_85.159 | 0.32 | 378 | 182 | 1.00E-51 | Gustatory receptor for sugar taste 64e OS=Drosophila melanogaster GN=Gr64e PE=2 SV=2 |
| TF326938 | evm.model.scaffold_109.56 | NA | NA | NA | NA | No blast hit |
| TF328339 | evm.model.scaffold_451.3 | 0.36 | 588 | 354 | 1.00E-108 | Transmembrane and TPR repeat-containing protein 3 OS=Homo sapiens GN=TMTC3 PE=1 SV=2 |
| TF328339 | evm.model.scaffold_471.136 | 0.54 | 208 | 223 | 1.00E-66 | Transmembrane and TPR repeat-containing protein CG4341 OS=Drosophila melanogaster GN=CG4341 PE=2 SV=1 |
| TF328339 | evm.model.scaffold_702.2 | 0.59 | 863 | 987 | 0 | Transmembrane and TPR repeat-containing protein CG4050 OS=Drosophila melanogaster GN=CG4050 PE=2 SV=1 |
| TF328339 | evm.model.scaffold_882.18 | 0.42 | 713 | 532 | 1.00E-179 | Transmembrane and TPR repeat-containing protein 4 OS=Mus musculus GN=Tmtc4 PE=2 SV=1 |
| TF351403 | evm.model.scaffold_26.69 | 0.82 | 606 | 1076 | 0 | Soluble guanylate cyclase 88E OS=Drosophila melanogaster GN=Gyc88E PE=1 SV=3 |
| TF351403 | evm.model.scaffold_471.130_evm.model.scaffold_471.131 | 0.51 | 702 | 673 | 0 | Head-specific guanylate cyclase OS=Drosophila melanogaster GN=Gycalpha99B PE=2 SV=2 |
| TF351919 | evm.model.scaffold_739.80 | 0.28 | 167 | 64.3 | 7.00E-11 | Leucine-rich alpha-2-glycoprotein OS=Homo sapiens GN=LRG1 PE=1 SV=2 |
| TF354237 | evm.model.scaffold_544.6 | 0.88 | 332 | 636 | 0 | Actin, cytoplasmic OS=Branchiostoma belcheri PE=2 SV=1 |
| TF354237 | evm.model.scaffold_675.10 | 0.98 | 376 | 781 | 0 | Actin, muscle OS=Manduca sexta PE=2 SV=1 |
| TF354237 | evm.model.scaffold_81.31 | 0.98 | 374 | 772 | 0 | Actin, clone 211 OS=Artemia sp. PE=2 SV=1 |
| TF354287 | evm.model.scaffold_1.8 | 0.24 | 489 | 150 | 7.00E-38 | 4-coumarate--CoA ligase-like 9 OS=Arabidopsis thaliana GN=4CLL9 PE=1 SV=2 |
| TF354287 | evm.model.scaffold_102.90 | 0.4 | 531 | 357 | 1.00E-115 | Probable 4-coumarate--CoA ligase 2 OS=Oryza sativa subsp. japonica GN=4CL2 PE=2 SV=2 |
| TF354287 | evm.model.scaffold_400.88 | 0.27 | 570 | 192 | 2.00E-53 | Luciferin 4-monooxygenase OS=Photuris pennsylvanica PE=2 SV=2 |
| TF354287 | evm.model.scaffold_463.82 | 0.41 | 552 | 414 | 1.00E-138 | Luciferin 4-monooxygenase OS=Luciola cruciata PE=1 SV=1 |
| TF354287 | evm.model.scaffold_647.62 | 0.37 | 551 | 298 | 4.00E-93 | 4-coumarate--CoA ligase 2 OS=Arabidopsis thaliana GN=4CL2 PE=1 SV=2 |
| TF354287 | evm.model.scaffold_647.63 | 0.37 | 534 | 341 | 1.00E-109 | Probable 4-coumarate--CoA ligase 3 OS=Oryza sativa subsp. japonica GN=4CL3 PE=2 SV=1 |
| TF354287 | evm.model.scaffold_832.18 | 0.27 | 522 | 179 | 4.00E-48 | 4-coumarate--CoA ligase 1 OS=Petroselinum crispum GN=4CL1 PE=2 SV=1 |
| TF354287 | evm.model.scaffold_94.157_evm.model.scaffold_94.158 | 0.42 | 498 | 377 | 1.00E-122 | Luciferin 4-monooxygenase OS=Luciola cruciata PE=1 SV=1 |
