# Supplementary figures and images for: Chromosome-level genome assembly and population genomic analyses provide insights into adaptive evolution of the red turpentine beetle, Dendroctonus valens
Source: BMC Biol. 2022 Aug 24;20:190. doi: 10.1186/s12915-022-01388-y (PMC9400205; doi:10.1186/s12915-022-01388-y)

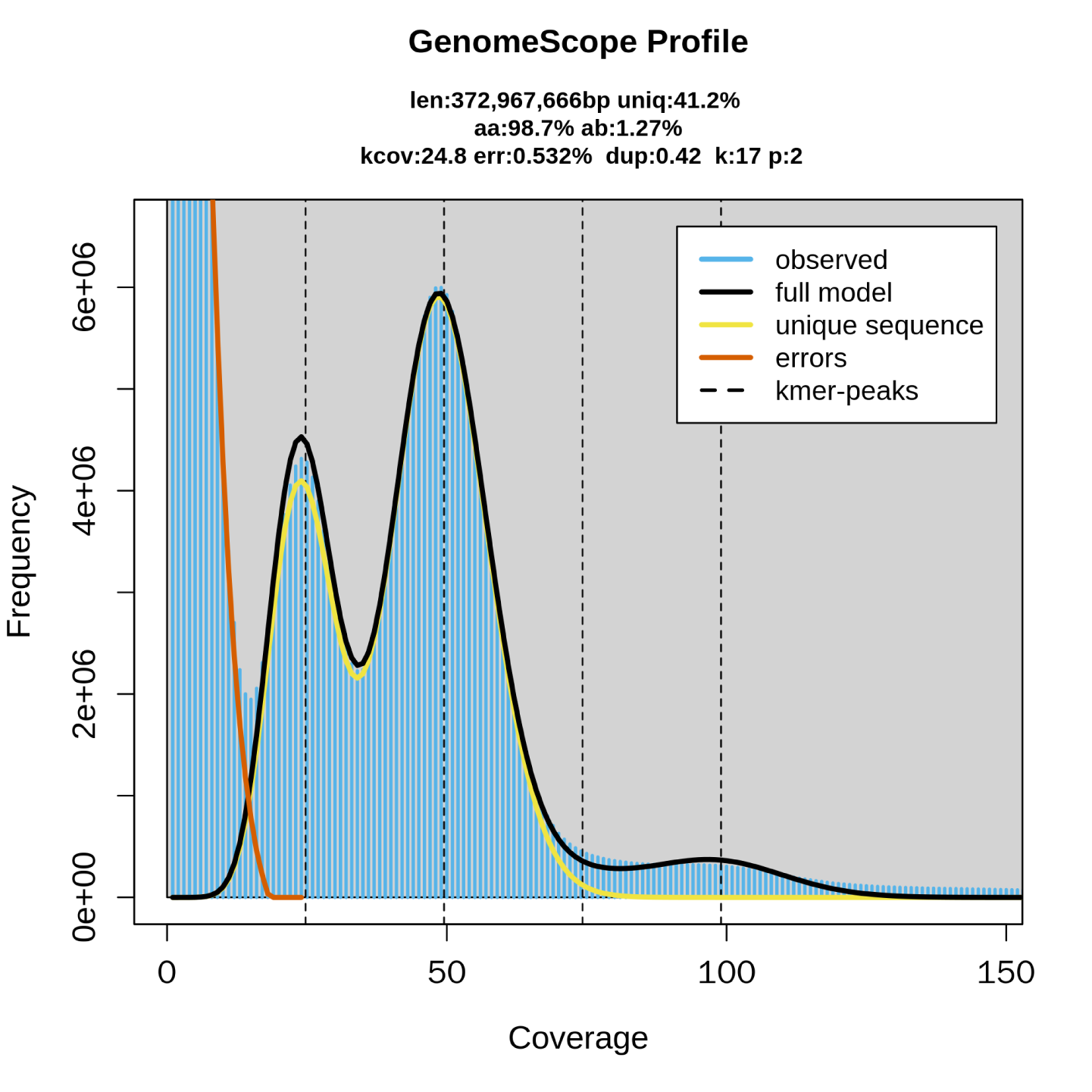

Supplement: Supplementary file 2 — Additional file 2: Figure S1. Genome survey result based on k-mer frequency analysis. K-mer frequency analysis was performed using Jellyfish (k-mer = 17) based on Illumina paired-end sequencing reads of genomic DNA. Genome size, repeat sequence content, and heterozygosity ratio were estimated based on k-mer frequency distribution using GenomeScope 2.0. The estimated genome size was 372.97 Mb. Figure S2. Linkage group contact map informed by Hi-C sequencing data in the red turpentine beetle genome. Fourteen linkage groups were generated after the clustering of contact map. The color bar indicates the frequency of Hi-C interaction intensity from low (yellow) to high (red) in the plot. Figure S3. Venn diagram showing the common and unique gene families across four Coleoptera species. Gene families were assigned by TreeFam database in four Coleoptera species, the red turpentine beetle Dendroctonus valens, the mountain pine beetle Dendroctonus ponderosae, the red flour beetle Tribolium castaneum, and the Asian long-horned beetle Anoplophora glabripennis. Figure S4. Synteny analysis between Dendroctonus valens and two closely related species. Dot plot representation of the syntenic relationship between D. valens and the species in the same genus, Dendroctonus ponderosae. Notably, D. valens linkage groups (LGs) showed strong syntenic relationship with D. ponderosae pseudo-chromosomes. Additionally, many fission and fusion events were observed between D. valens and D. ponderosae. (b) Genome-wide synteny relationship between D. valens and two Coleoptera insects, D. ponderosae and Tribolium castaneum. As shown in the figure, Dpochr1 was formed by the fusion of four complete LGs in D. valens (i.e. LG1, LG4, LG10, and LG11). By contrast, Dpochr9 fused with Dpochr12 to generate LG13 of D. valens. Genome-wide synteny analysis was performed using the MCScan pipeline of JCVI utility libraries. Figure S5. Global representation of sampling sites of different geographical populations [file 12915_2022_1388_MOESM2_ESM.zip › Figure S1.tif]

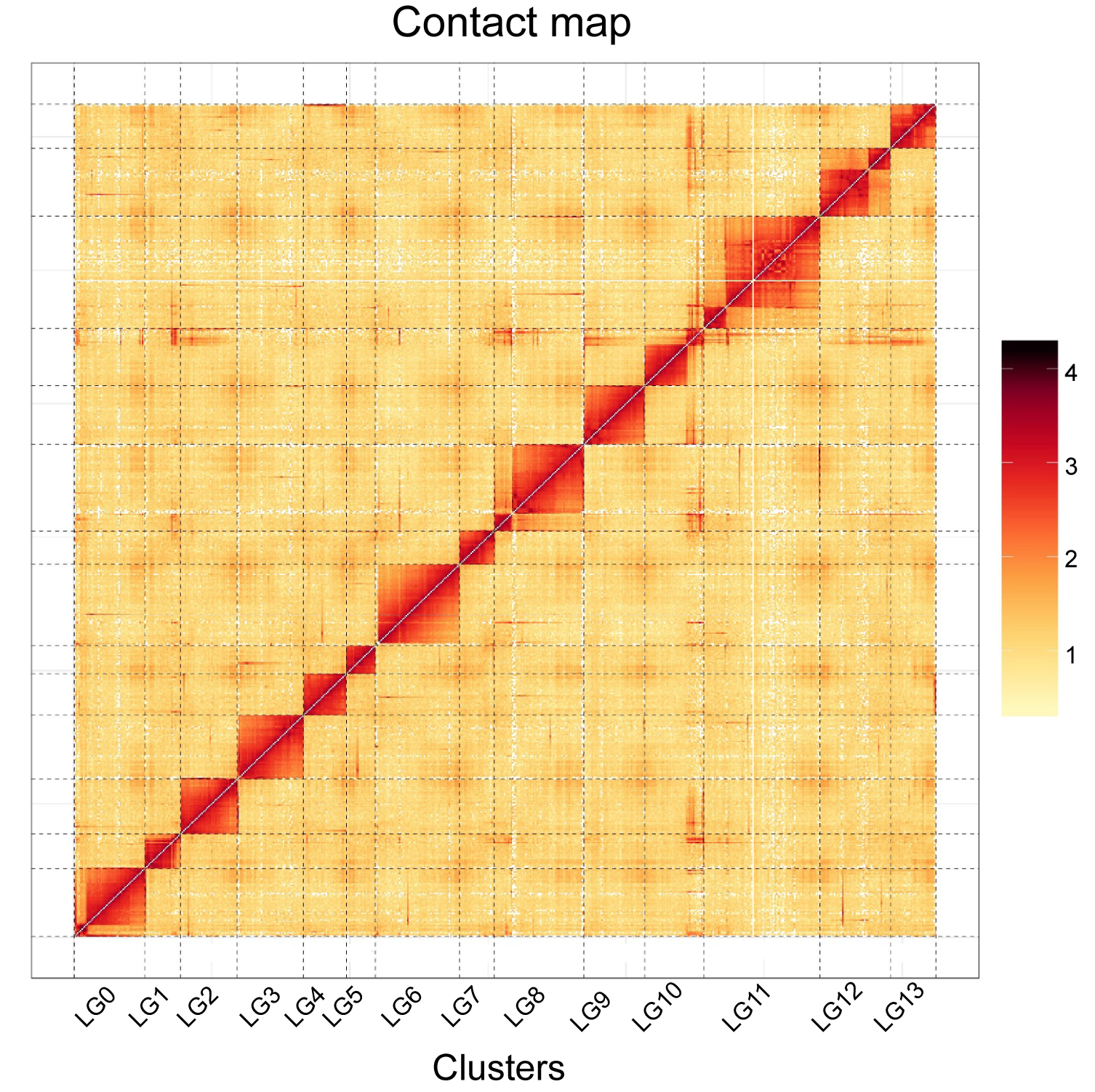

Supplement: Supplementary file 2 — Additional file 2: Figure S1. Genome survey result based on k-mer frequency analysis. K-mer frequency analysis was performed using Jellyfish (k-mer = 17) based on Illumina paired-end sequencing reads of genomic DNA. Genome size, repeat sequence content, and heterozygosity ratio were estimated based on k-mer frequency distribution using GenomeScope 2.0. The estimated genome size was 372.97 Mb. Figure S2. Linkage group contact map informed by Hi-C sequencing data in the red turpentine beetle genome. Fourteen linkage groups were generated after the clustering of contact map. The color bar indicates the frequency of Hi-C interaction intensity from low (yellow) to high (red) in the plot. Figure S3. Venn diagram showing the common and unique gene families across four Coleoptera species. Gene families were assigned by TreeFam database in four Coleoptera species, the red turpentine beetle Dendroctonus valens, the mountain pine beetle Dendroctonus ponderosae, the red flour beetle Tribolium castaneum, and the Asian long-horned beetle Anoplophora glabripennis. Figure S4. Synteny analysis between Dendroctonus valens and two closely related species. Dot plot representation of the syntenic relationship between D. valens and the species in the same genus, Dendroctonus ponderosae. Notably, D. valens linkage groups (LGs) showed strong syntenic relationship with D. ponderosae pseudo-chromosomes. Additionally, many fission and fusion events were observed between D. valens and D. ponderosae. (b) Genome-wide synteny relationship between D. valens and two Coleoptera insects, D. ponderosae and Tribolium castaneum. As shown in the figure, Dpochr1 was formed by the fusion of four complete LGs in D. valens (i.e. LG1, LG4, LG10, and LG11). By contrast, Dpochr9 fused with Dpochr12 to generate LG13 of D. valens. Genome-wide synteny analysis was performed using the MCScan pipeline of JCVI utility libraries. Figure S5. Global representation of sampling sites of different geographical populations [file 12915_2022_1388_MOESM2_ESM.zip › Figure S2.tif]

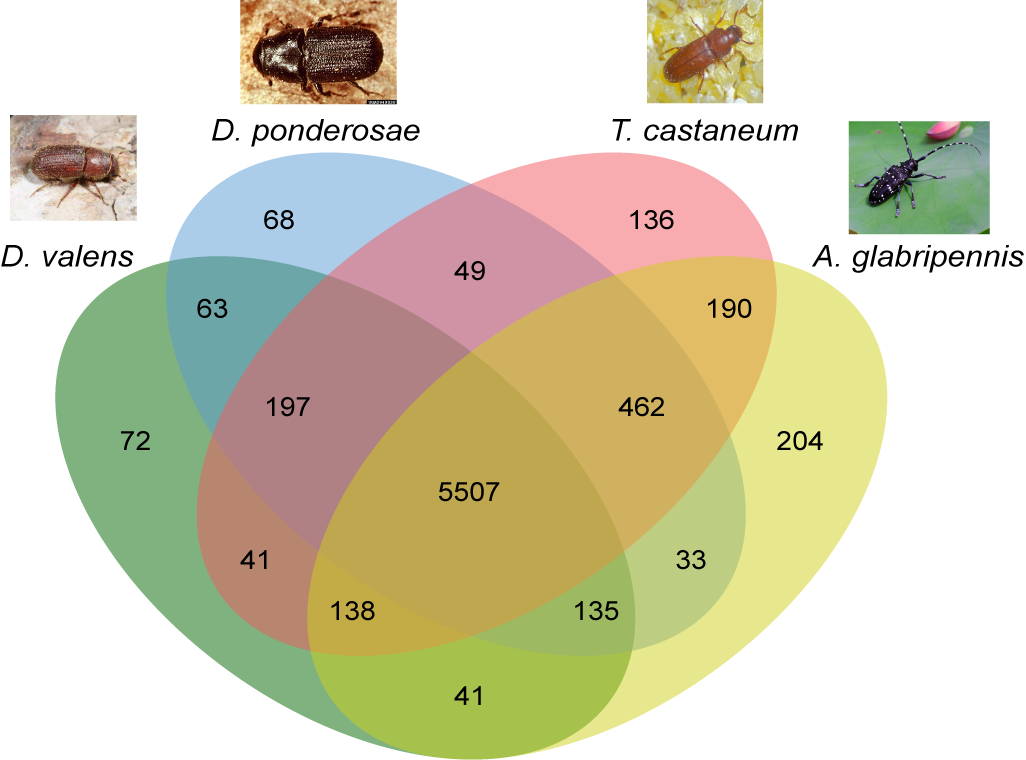

Supplement: Supplementary file 2 — Additional file 2: Figure S1. Genome survey result based on k-mer frequency analysis. K-mer frequency analysis was performed using Jellyfish (k-mer = 17) based on Illumina paired-end sequencing reads of genomic DNA. Genome size, repeat sequence content, and heterozygosity ratio were estimated based on k-mer frequency distribution using GenomeScope 2.0. The estimated genome size was 372.97 Mb. Figure S2. Linkage group contact map informed by Hi-C sequencing data in the red turpentine beetle genome. Fourteen linkage groups were generated after the clustering of contact map. The color bar indicates the frequency of Hi-C interaction intensity from low (yellow) to high (red) in the plot. Figure S3. Venn diagram showing the common and unique gene families across four Coleoptera species. Gene families were assigned by TreeFam database in four Coleoptera species, the red turpentine beetle Dendroctonus valens, the mountain pine beetle Dendroctonus ponderosae, the red flour beetle Tribolium castaneum, and the Asian long-horned beetle Anoplophora glabripennis. Figure S4. Synteny analysis between Dendroctonus valens and two closely related species. Dot plot representation of the syntenic relationship between D. valens and the species in the same genus, Dendroctonus ponderosae. Notably, D. valens linkage groups (LGs) showed strong syntenic relationship with D. ponderosae pseudo-chromosomes. Additionally, many fission and fusion events were observed between D. valens and D. ponderosae. (b) Genome-wide synteny relationship between D. valens and two Coleoptera insects, D. ponderosae and Tribolium castaneum. As shown in the figure, Dpochr1 was formed by the fusion of four complete LGs in D. valens (i.e. LG1, LG4, LG10, and LG11). By contrast, Dpochr9 fused with Dpochr12 to generate LG13 of D. valens. Genome-wide synteny analysis was performed using the MCScan pipeline of JCVI utility libraries. Figure S5. Global representation of sampling sites of different geographical populations [file 12915_2022_1388_MOESM2_ESM.zip › Figure S3.tif]

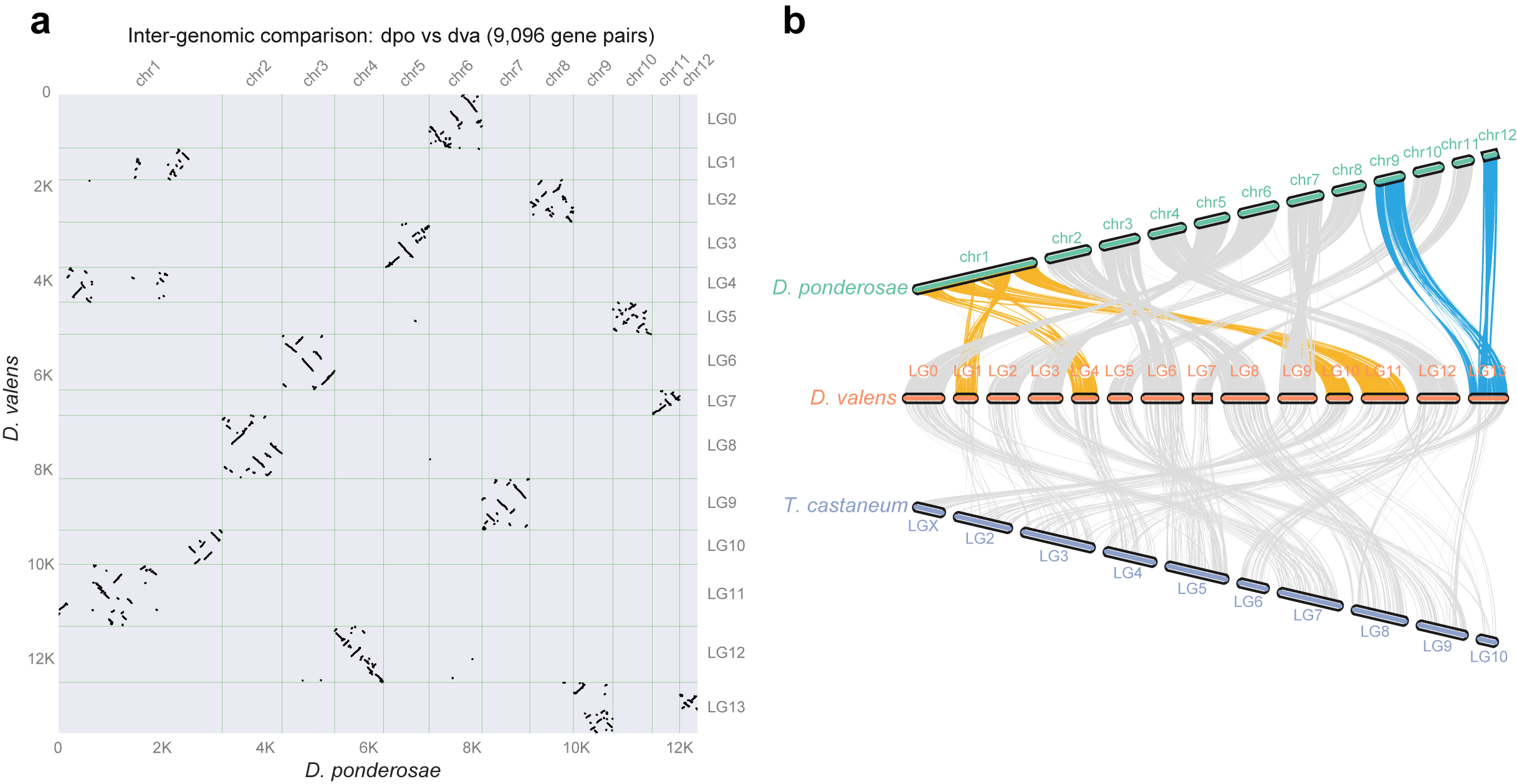

Supplement: Supplementary file 2 — Additional file 2: Figure S1. Genome survey result based on k-mer frequency analysis. K-mer frequency analysis was performed using Jellyfish (k-mer = 17) based on Illumina paired-end sequencing reads of genomic DNA. Genome size, repeat sequence content, and heterozygosity ratio were estimated based on k-mer frequency distribution using GenomeScope 2.0. The estimated genome size was 372.97 Mb. Figure S2. Linkage group contact map informed by Hi-C sequencing data in the red turpentine beetle genome. Fourteen linkage groups were generated after the clustering of contact map. The color bar indicates the frequency of Hi-C interaction intensity from low (yellow) to high (red) in the plot. Figure S3. Venn diagram showing the common and unique gene families across four Coleoptera species. Gene families were assigned by TreeFam database in four Coleoptera species, the red turpentine beetle Dendroctonus valens, the mountain pine beetle Dendroctonus ponderosae, the red flour beetle Tribolium castaneum, and the Asian long-horned beetle Anoplophora glabripennis. Figure S4. Synteny analysis between Dendroctonus valens and two closely related species. Dot plot representation of the syntenic relationship between D. valens and the species in the same genus, Dendroctonus ponderosae. Notably, D. valens linkage groups (LGs) showed strong syntenic relationship with D. ponderosae pseudo-chromosomes. Additionally, many fission and fusion events were observed between D. valens and D. ponderosae. (b) Genome-wide synteny relationship between D. valens and two Coleoptera insects, D. ponderosae and Tribolium castaneum. As shown in the figure, Dpochr1 was formed by the fusion of four complete LGs in D. valens (i.e. LG1, LG4, LG10, and LG11). By contrast, Dpochr9 fused with Dpochr12 to generate LG13 of D. valens. Genome-wide synteny analysis was performed using the MCScan pipeline of JCVI utility libraries. Figure S5. Global representation of sampling sites of different geographical populations [file 12915_2022_1388_MOESM2_ESM.zip › Figure S4.tif]

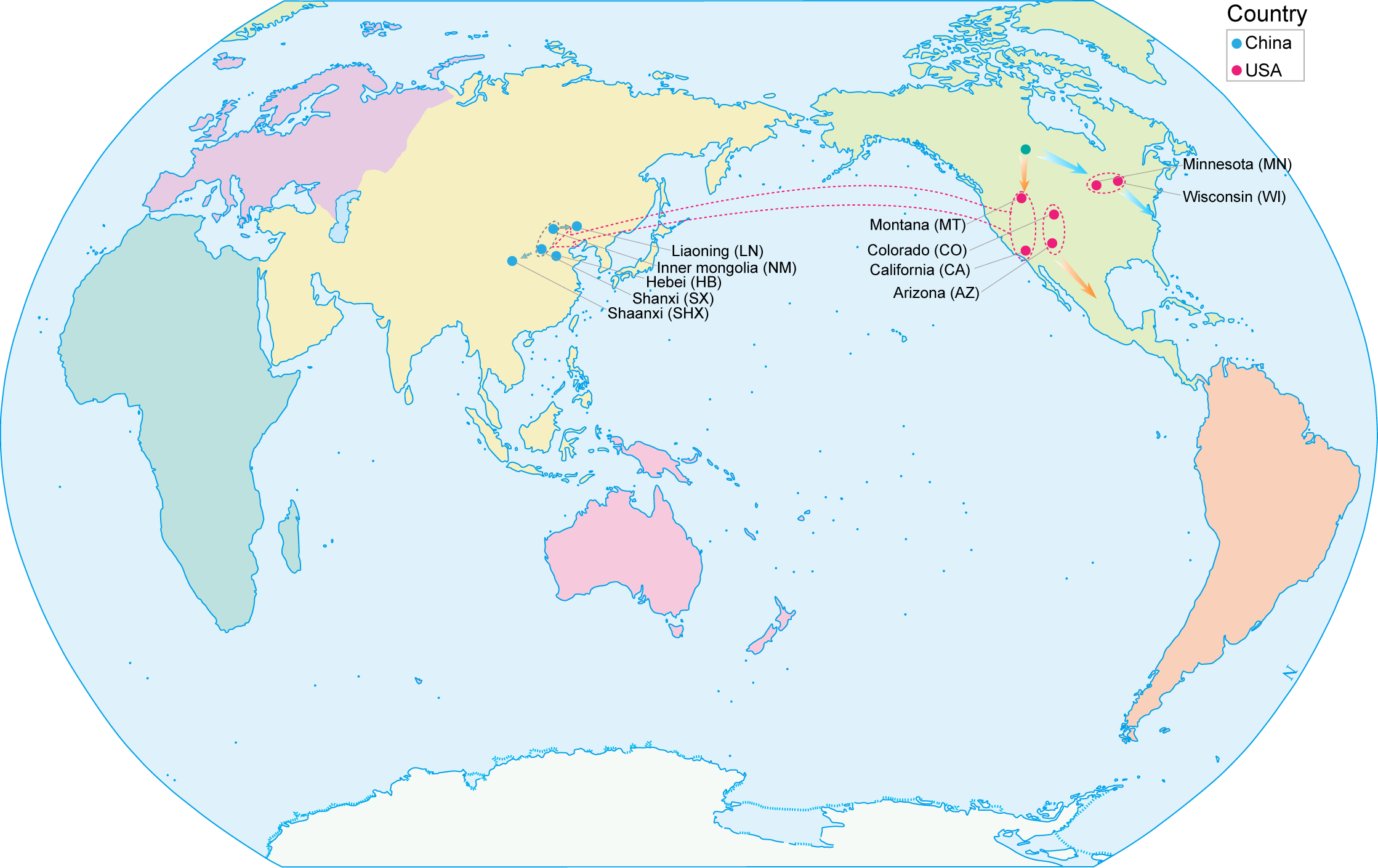

Supplement: Supplementary file 2 — Additional file 2: Figure S1. Genome survey result based on k-mer frequency analysis. K-mer frequency analysis was performed using Jellyfish (k-mer = 17) based on Illumina paired-end sequencing reads of genomic DNA. Genome size, repeat sequence content, and heterozygosity ratio were estimated based on k-mer frequency distribution using GenomeScope 2.0. The estimated genome size was 372.97 Mb. Figure S2. Linkage group contact map informed by Hi-C sequencing data in the red turpentine beetle genome. Fourteen linkage groups were generated after the clustering of contact map. The color bar indicates the frequency of Hi-C interaction intensity from low (yellow) to high (red) in the plot. Figure S3. Venn diagram showing the common and unique gene families across four Coleoptera species. Gene families were assigned by TreeFam database in four Coleoptera species, the red turpentine beetle Dendroctonus valens, the mountain pine beetle Dendroctonus ponderosae, the red flour beetle Tribolium castaneum, and the Asian long-horned beetle Anoplophora glabripennis. Figure S4. Synteny analysis between Dendroctonus valens and two closely related species. Dot plot representation of the syntenic relationship between D. valens and the species in the same genus, Dendroctonus ponderosae. Notably, D. valens linkage groups (LGs) showed strong syntenic relationship with D. ponderosae pseudo-chromosomes. Additionally, many fission and fusion events were observed between D. valens and D. ponderosae. (b) Genome-wide synteny relationship between D. valens and two Coleoptera insects, D. ponderosae and Tribolium castaneum. As shown in the figure, Dpochr1 was formed by the fusion of four complete LGs in D. valens (i.e. LG1, LG4, LG10, and LG11). By contrast, Dpochr9 fused with Dpochr12 to generate LG13 of D. valens. Genome-wide synteny analysis was performed using the MCScan pipeline of JCVI utility libraries. Figure S5. Global representation of sampling sites of different geographical populations [file 12915_2022_1388_MOESM2_ESM.zip › Figure S5.tif]

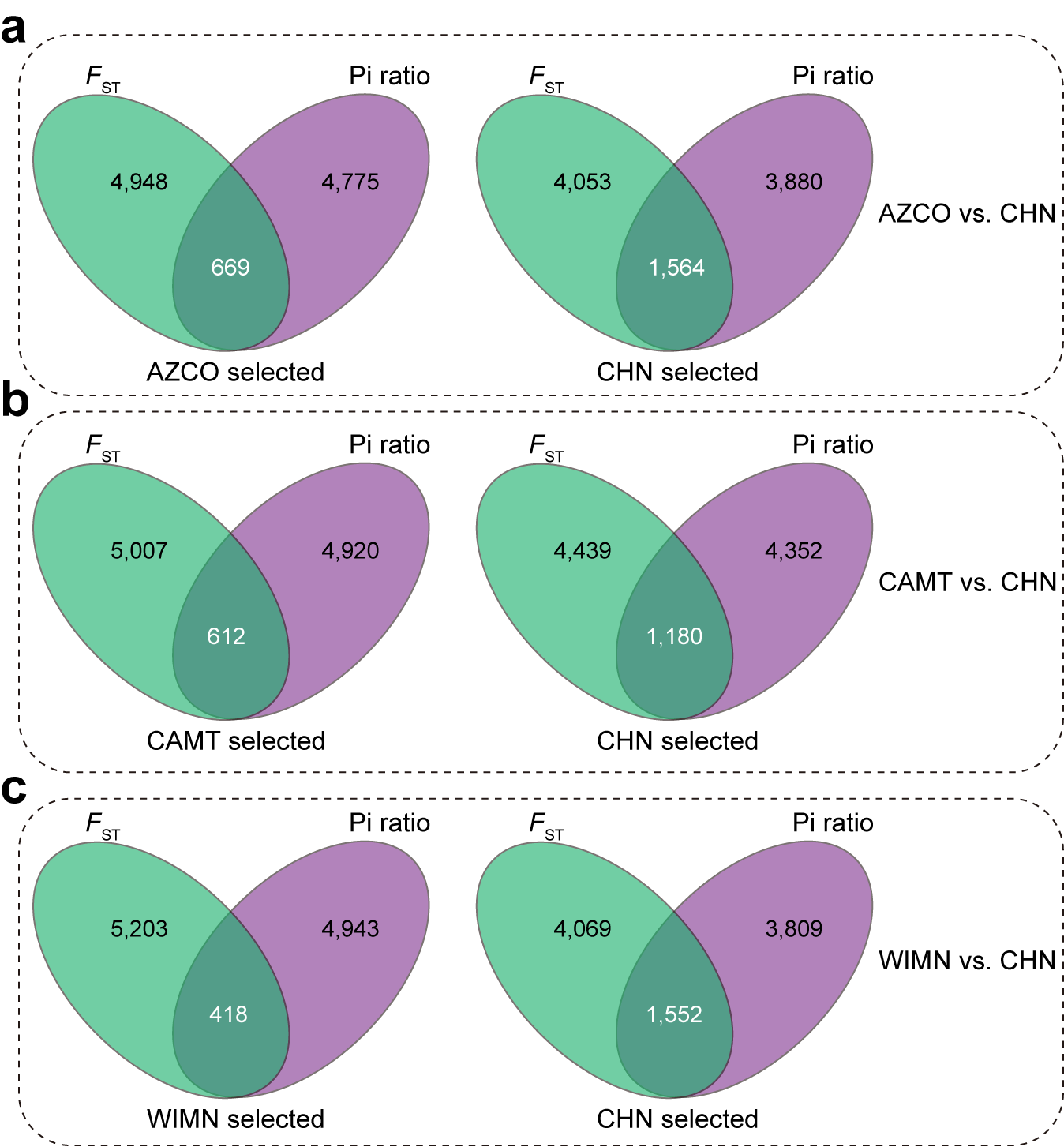

Supplement: Supplementary file 2 — Additional file 2: Figure S1. Genome survey result based on k-mer frequency analysis. K-mer frequency analysis was performed using Jellyfish (k-mer = 17) based on Illumina paired-end sequencing reads of genomic DNA. Genome size, repeat sequence content, and heterozygosity ratio were estimated based on k-mer frequency distribution using GenomeScope 2.0. The estimated genome size was 372.97 Mb. Figure S2. Linkage group contact map informed by Hi-C sequencing data in the red turpentine beetle genome. Fourteen linkage groups were generated after the clustering of contact map. The color bar indicates the frequency of Hi-C interaction intensity from low (yellow) to high (red) in the plot. Figure S3. Venn diagram showing the common and unique gene families across four Coleoptera species. Gene families were assigned by TreeFam database in four Coleoptera species, the red turpentine beetle Dendroctonus valens, the mountain pine beetle Dendroctonus ponderosae, the red flour beetle Tribolium castaneum, and the Asian long-horned beetle Anoplophora glabripennis. Figure S4. Synteny analysis between Dendroctonus valens and two closely related species. Dot plot representation of the syntenic relationship between D. valens and the species in the same genus, Dendroctonus ponderosae. Notably, D. valens linkage groups (LGs) showed strong syntenic relationship with D. ponderosae pseudo-chromosomes. Additionally, many fission and fusion events were observed between D. valens and D. ponderosae. (b) Genome-wide synteny relationship between D. valens and two Coleoptera insects, D. ponderosae and Tribolium castaneum. As shown in the figure, Dpochr1 was formed by the fusion of four complete LGs in D. valens (i.e. LG1, LG4, LG10, and LG11). By contrast, Dpochr9 fused with Dpochr12 to generate LG13 of D. valens. Genome-wide synteny analysis was performed using the MCScan pipeline of JCVI utility libraries. Figure S5. Global representation of sampling sites of different geographical populations [file 12915_2022_1388_MOESM2_ESM.zip › Figure S6.tif]
